# Complete chloroplast genomes of *Achnatherum inebrians* and comparative analyses with related species from Poaceae

**DOI:** 10.1002/2211-5463.13170

**Published:** 2021-05-10

**Authors:** Xuekai Wei, Xiuzhang Li, Taixiang Chen, Zhenjiang Chen, Yuanyuan Jin, Kamran Malik, Chunjie Li

**Affiliations:** ^1^ State Key Laboratory of Grassland Agro‐ecosystems, Key Laboratory of Grassland Livestock Industry Innovation Ministry of Agriculture and Rural Affairs Engineering Research Center of Grassland Industry Ministry of Education Gansu Tech Innovation Centre of Western China Grassland Industry Centre for Grassland Microbiome College of Pastoral Agriculture Science and Technology Lanzhou University China; ^2^ Qinghai Academy of Animal and Veterinary Science Qinghai University Xining China

**Keywords:** *Achnatherum inebrians*, chloroplast genomes, comparative analysis, phylogenetic analysis, Poaceae

## Abstract

This article reports the complete chloroplast genome of *Achnatherum inebrians*, a poisonous herb that is widely distributed in the rangelands of Northern China. The genome is 137 714 bp in total and consists of a large single‐copy (81 758 bp) region and small single‐copy (12 682 bp) region separated by a pair of inverted repeats (21 637 bp). The genome contains 130 genes, including 84 protein‐coding genes, 38 tRNA genes and 8 ribosomal RNA genes, and the guanine + cytosine content is 36.17%. We subsequently performed comparative analysis of complete genomes from *A*. *inebrians* and other Poaceae‐related species from GenBank. Thirty‐eight simple sequence repeats were identified, further demonstrating rapid evolution in Poaceae. Finally, the phylogenetic trees of 37 species of Poaceae and 2 species of Amaranthaceae were constructed by using maximum likelihood and Bayesian inference methods, based on the genes of the complete chloroplast genome. We identified hotspots that can be used as molecular markers and barcodes for phylogenetic analysis, as well as for species identification. Phylogenetic analysis indicated that *A. inebrians* is a member of the genus *Stipa* rather than *Achnatherum*.

## Graphical abstract

The complete genomes from *Achnatherum inebrians* and other Poaceae‐related species from GenBank were subjected to comparative analysis. We not only identified hotspots that can be used as molecular markers and barcodes for phylogenetic analysis and species identification but also identified phylogenetic evidence that *A. inebrians* is a member of the genus *Stipa* sp. rather than *Achnatherum* sp.

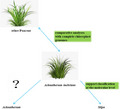

**Abbreviations**
BI: Bayesian inference
GC: guanine + cytosine
IR: inverted repeat
IRa: inverted repeat region a
IRb: inverted repeats region b
LSC: large single copy
ML: maximum likelihood
NCBI: National Center for Biotechnology Information
Pi: nucleotide variation
RSCU: relative synonymous codon usage
SSC: small single copy
SSR: simple sequence repeat

*Achnatherum inebrians* is a common and widespread perennial toxic grass in the semiarid grassland regions of northern China [1]. In earlier classification, *A. inebrians* was named as *Stipa inebrians*, but Geng [2, 3] revised its classification from *Stipa* to *Achnatherum* (Gramineae, Pooideae, Stipeae), which is still used today. Chu and Yang [4] identified *A. inebrians* as the section [sect. *Achnatheropsis* (Tzvel.) Q.G.Chu.comb.nov.] according to the external morphology of the genus *Achnatherum* in 1990. This grass is majorly involved in reverse degradation and loss of biodiversity of overgrazed grasslands, while it serves as a diversity refuge for the soil fungal community [5, 6]. In Northwestern China, almost all the plants of *A. inebrians* are infected by a symptomless fungal endophyte, *Epichloë* (*Epichloë gansuensis* or *Epichloë inebrian*) [7, 8, 9]. *Achnatherum inebrians* is commonly referred to as drunken horse grass because of the presence of two alkaloids produced in *Epichloë* endophyte‐infected *A. inebrians* plants, ergonovine and ergine, which cause toxicity or death to horses and other livestock [10, 11, 12]. The presence of *Epichloë* endophytes in aboveground tissues can regulate the metabolic processes of host grasses, including promoting plant growth and enhancing the tolerance of host plants to various biotic and abiotic stresses, such as heavy metals, low temperature, drought and salinity [13, 14, 15, 16, 17, 18, 19].

Chloroplasts are small photosynthetic machinery and carbon fixation organelles that are present in algae and plant cells. Most chloroplast‐encoded proteins are responsible for photosynthesis and the synthesis of fatty acids and amino acids [20, 21]. Chloroplasts have their own genetic system, consisting of a closed circular structure ranging from 115 to 165 kb in length, a small single‐copy (SSC) region, a large single‐copy (LSC) region and a pair of inverted repeats (IRs) [22, 23, 24, 25]. Compared with nuclear genomes, chloroplast genomes have fewer nucleotide substitutions and rearrangements of genome structures, moderate genome size, and desirable collinear properties among different species, providing an ideal model to decipher genomic evolution and phylogenetic relationships in angiosperms [26, 27]. High‐throughput sequencing technology has stimulated the rapid development of chloroplast genome sequencing [28] and enabled the study of evolutionary dynamics at a more taxonomically complex level (species or lower level) [29].

*Achnatherum* species are poorly studied from a genomic perspective. To date, chloroplast genomes are available for only one representative, *Achnatherum splendens* [30]. This study for the first time reports the complete chloroplast genome sequence of *A. inebrians*, including a description of its general features, IR contraction and expansion, codon usage and analysis of simple sequence repeats (SSRs). In addition, we compared the gene contents, organization, and phylogenetic relationships with other chloroplast genomes in Poaceae, which will help improve the understanding of chloroplast genome characteristics, structural diversity and evolution within Poaceae.

## Materials and methods

### Sample collection and DNA extraction

Fresh *A. inebrians* leaves were collected from alpine grassland in Tianzhu county (37°11′N, 102°47′E), Gansu province, China. For chloroplast genome DNA extraction, the collected fresh pieces were immediately placed in liquid nitrogen and stored at −80°C until chloroplast genome DNA was extracted. The voucher specimen was stored at the Official Herbage and Turfgrass Seed Testing Centre, Ministry of Agriculture, Lanzhou, China. Total genomic DNA was extracted using the hexadecyltrimethyl ammonium bromide method, and the quality of chloroplast genome was measured by NanoDrop 2000 (Thermo Scientific, Wilmington, NC, USA) and agarose gel electrophoresis. The quantified DNA (260/280 value is 1.6–1.8, and the concentration is >20 ng·μL^−1^; the band is about 5K) was used for library construction.

### Library preparation and sequencing and genome assembly

The qualified library was sequenced with Illumina NovaSeq (Wuhan Benagen Tech Solutions Company Limited, Wuhan, China). The raw sequencing data were filtered with low‐quality data to obtain effective data. soapnuke (Version: 2.1.0; Wuhan Benagen Tech Solutions Company Limited, Wuhan, Hubei, China) was used as the filtering software for the project, and the filtering standards were as follows: (a) remove reads with N base content exceeding 5%, (b) remove reads with low mass (Q score ≤ 5) and the number of bases reaches 50%, and (c) remove the adapter sequence contained in reads. The Illumina NovaSeq sequester was used for paired‐end sequencing, and the reads length was 150 bp, which in pieces was done by nucleic acid shear (Covaris M220; USA) apparatus [centrifuge at 3000 ***g*** (relative centrifugal force) for 1 min].

Chloroplast genome assembly was performed using novoplasty software (version 3.2; parameter: *k*‐mer = 39; https://github.com/ndierckx/novoplasty), and the published gene sequence of the target species was selected as the seed sequence (JF698225.1) to splice chloroplast genomes. The joining together with the relative chloroplast genome (NC_029390.1) was blastn (version: blast 2.9.0+; parameter: −*e* value, 1e−5; ftp://ftp.ncbi.nlm.nih.gov/blast/executables/blast+/LATEST/) alignment, which adjusts the order of target sequences based on alignment with related species. If the connected sequence contains gap (including N sequence), then gapcloser (version 1.12; https://github.com/aquaskyline/SOAPdenovo2) was used to further fill the hole to obtain the final stitching result.

### Genome annotation and comparative genome analyses

Chloroplast genome functional annotation includes encoding gene prediction and noncoding RNA annotation (rRNA and tRNA annotations). Gene annotation was performed using CPGAVAS2 [31], and the map of the circular *A. inebrians* chloroplast genome was drawn through the online tool Chloroplot [32].

The distribution of codon usage was detected by using codonw (version 1.4.4; https://sourceforge.net/projects/codonw/) with the relative synonymous codon usage (RSCU) ratio [33]. The codon of *A. inebrians* chloroplast was visually compared among species of 17 Poaceae with r language and tbtools [34].

The *A. inebrians* chloroplast genome was compared with the other five chloroplast genomes using the Shuffle–Lagan model of the mvista program [35]; *Alopecurus japonicus* served as the reference. irscope was used to visualize the boundaries between the IR and SC regions of *A. inebrians*, and the results were compared and analyzed with three other Poaceae species [36]. The four chloroplast genomes of Poaceae were initially compared using mafft [37] and then manually adjusted using bioedit [38]. Variable sites and nucleotide variations (Pi) in the entire chloroplast genome and LSC, IR and SSC regions of four species were calculated using dnasp [39].

### Repeat sequence analyses

The SSRs of *A. inebrians* and three other chloroplast genomes were identified using the online web tool misa (version 2.1) [40]. The parameter sets of the minimum number of repetitions of SSRs for mononucleotides, dinucleotides, trinucleotides, tetranucleotides, pentanucleotides and hexanucleotides were 10, 5, 4, 3, 3 and 3, respectively.

### Phylogenetic analyses

Phylogenetic relationships were reconstructed by using the complete *A. inebrians* chloroplast genome and 36 other Poaceae chloroplast genomes submitted in the National Center for Biotechnology Information (NCBI); *Cyperus rotundus* and *Eleocharis dulcis* were used as outgroups. All species and accession numbers of the chloroplast genomes in NCBI are listed in Table S1. Phylogenetic analysis was conducted on the phylosuite version 1.2.2 platform [41]. The nucleotide sequence of the whole chloroplast genome was aligned in mafft based on default parameters [37]. Ambiguously aligned fragments were removed using gblocks [42], with the following parameter settings: minimum number of sequences for a conserved/flank position (20/20), maximum number of contiguous nonconserved positions (6), minimum length of a block (11) and allowed gap positions (0). ModelFinder [43] was used to select the best‐fit model using Akaike information criterion. Maximum‐likelihood (ML) phylogenies were inferred using iq‐tree [44] under the GTR+R4+F model for 5000 ultrafast [45] bootstraps, approximate Bayes test [46] and the Shimodaira–Hasegawa‐like approximate likelihood‐ratio test [47]. Bayesian inference (BI) phylogenies were inferred using mrbayes 3.2.0 [48] under the GTR+I+G+F model (two parallel runs and 1 000 000 generations), in which the initial 25% of sampled data were discarded as burn‐in. The generated trees were visualized using the online web tool iTOL [49].

## Results

### Chloroplast genome assembly and genome features

**Fig. 1:**
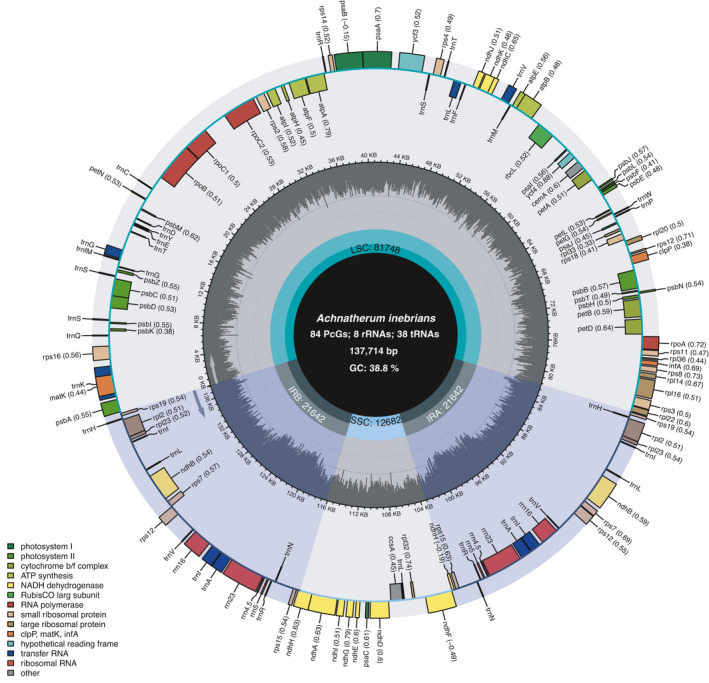
Chloroplast genome map of *Achnatherum* *inebrians*. The center of the figure provides the specific information (length, GC content and number of genes) of the *A. inebrians* chloroplast genome. In the first inner circle, the proportion of the shaded parts represents the GC content of each part. The lengths of the corresponding small single‐copy (SSC), IR (IRa and IRb) and LSC regions are also listed. The gene names and their optional codon usage bias are labeled on the outermost layer. The transcription directions for the inner and outer genes are listed clockwise and anticlockwise, respectively.

The genome size of the complete chloroplast genome of *A. inebrians* was 137 714 bp in length, with chloroplast circular molecules having quadripartite structures composed of IRa (21 637 bp) and IRb (21 637 bp) regions, separated by the LSC (81 758 bp) and SSC (12 682 bp) regions (Table 1; Fig. 1). The guanine + cytosine (GC) content of the complete chloroplast genomes was 38.8%, while LSC, SSC and IR regions showed 36.8%, 33.1% and 44.1% GC contents, respectively.

**Table 1:** Summary of complete chloroplast genomes for *Achnatherum inebrians*, *Achnatherum splendens*, *Stipa hymenoides*, and *Stipa purpurea*.

Item	*Achnatherum inebrians*	*Achnatherum splendens*	*Stipa hymenoides*	*Stipa purpurea*
Total size (bp)	137 714	136 876	137 742	137 370
LSC size (bp)	81 758	80 958	81 709	81 202
SSC size (bp)	12 682	12 640	12 803	12 842
IR size (bp)	21 637	21 639	21 615	21 663
Total GC content (%)	38.8	38.9	38.8	38.8
LSC GC content (%)	36.8	36.7	36.9	36.9
SSC GC content (%)	33.1	33.3	33.6	32.9
IR GC content (%)	44.1	44.2	44.1	44.1
Number of genes	130	130	130	130
Number of protein‐coding genes	84	84	84	84
Number of tRNA genes	38	38	38	38
Number of rRNA genes	8	8	8	8

A total of 130 genes were found in the whole chloroplast genome of *A*. *inebrians*, including 84 protein‐coding genes, 38 tRNA genes, 8 rRNA genes, and 2 pseudogenes (*ycf3* and *ycf4*; Table 1; Fig. 1). The protein‐coding genes include 11 genes for large ribosomal proteins [*rpl32*, *rpl14*, *rpl22*, *rpl33*, *rpl20*, *rpl36*, *rpl23* (×2), *rpl16*, *rpl2* (×2)], 16 for small ribosomal proteins [*rps3*, *rps16*, *rps8*, *rps11*, *rps12* (×2), *rps18*, *rps2*, *rps14*, *rps19* (×2), *rps15* (×2), *rps7* (×2), *rps4*], 5 for photosystem I (*psaJ*, *psaA*, *psaB*, *psaC*, *psaI*), 15 for photosystem II (*psbB*, *psbK*, *psbH*, *psbL*, *psbA*, *psbI*, *psbM*, *psbJ*, *psbT*, *psbC*, *psbZ*, *psbF*, *psbD*, *psbE*, *psbN*) and 6 for ATP synthase (Table 2).

**Table 2:** List of annotated genes in the chloroplast of *Achnatherum* *inebrians*.

Group	Gene group	Gene name
Self‐replication	Ribosomal proteins (LSU)	*rpl32*, *rpl14*, *rpl22*, *rpl33*, *rpl20*, *rpl36*, *rpl23* ^a^ (×2), *rpl16,* ^b^ *rpl2* ^a^, ^b^ (*×2*)
	Ribosomal proteins (SSU)	*rps3*, *rps16*,^b^ *rps8*, *rps11*, *rps12* ^a^, ^b^ (*×2*), *rps18*, *rps2*, *rps14*, *rps19* ^a^ (*×2*), *rps15* ^a^ (*×2*), *rps7* ^a^ (*×2*), *rps4*
	RNA polymerase	*rpoC2*, *rpoC1*, *rpoB*, *rpoA*
	rRNA gene	*rrn23* ^a^ (*×2*), *rrn5* ^a^ (*×2*), *rrn16* ^a^ (*×2*), *rrn4.5* ^a^ (*×2*)
	tRNA genetrnC‐GCA	*trnI‐CAU* ^a^ (*×2*), *trnS‐GGA*, *trnT‐GGU*, *trnC‐GCA*, *trnF‐GAA*, *trnN‐GUU* ^a^ (*×2*), *trnA‐UGC* ^a^, ^b^ (*×2*), *trnP‐UGG,trnL‐CAA* ^a^ (*×2*), *trnI‐GAU* ^a^, ^b^ (*×2*), *trnS‐GCU*, *trnG‐UCC,trnL‐UAG*, *trnR‐UCU*, *trnV‐GAC* ^a^ (*×2*), *trnT‐UGU*, *trnQ‐UUG*, *trnY‐GUA*, *trnR‐ACG* ^a^ (*×2*), *trnE‐UUC*, *trnW‐CCA, trnS‐UGA*, *trnH‐GUG* ^a^ (*×2*), *trnM‐CAU*, *trnK‐UUU*,^b^ *trnD‐GUC*, *trnV‐UAC*,^b^ *trnG‐GCC*, *trnfM‐CAU*, *trnL‐UAA* ^b^
Gene for photosynthesis	Subunits of photosystem I	*psaA*, *psaB*, *psaJ*, *psaI*, *psaC*
	Subunits of photosystem II	*psbB*, *psbK*, *psbH*, *psbL*, *psbA*, *psbI*, *psbM*, *psbJ*, *psbT*, *psbC*, *psbZ*, *psbF*, *psbD*, *psbE*, *psbN*
	Subunits of NADH dehydrogenase	*ndhG*, *ndhB* ^a^, ^b^ (*×2*), *ndhK*, *ndhD*, *ndhA*,^b^ *ndhH* ^a^ (*×2*), *ndhF*, *ndhC*, *ndhI*, *ndhJ*, *ndhE*
	Subunits of cytochrome b/f complex	*petA*, *petG*, *petB*,^b^ *petN*, *petD*,^b^ *petL*
	Subunits for ATP synthase	*atpE*, *atpH*, *atpI*, *atpA*, *atpB*, *atpF* ^b^
	Large subunit RuBisCO	*rbcL*
Other genes	Translational initiation factor	*infA*
	Maturase	*matK*
	Protease	*clpP*
	Envelope membrane protein	*cemA*
	C‐type cytochrome synthesis gene	*ccsA*
	Hypothetical chloroplast reading frames (*ycf*)	*ycf3*,^c^ *ycf4*

^a^ Genes located in the IRs.

^b^ Gene with one intron.

^c^ Gene with two introns.

In the chloroplast genome of *A. inebrians*, eight protein‐coding (*rps19*, *rpl2*, *rpl23*, *ndhB*, *nadH*, *rps7*, *rps12* and *rps15*), four rRNA (*rrn16*, *rrn23*, *rrn4.5* and *rrn5*) and eight tRNA genes (*trnA‐UGC*, *trnH‐GUG*, *trnI‐GAU*, *trnI‐CAU*, *trnL‐CAA*, *trnN‐GUU*, *trnR‐ACG* and *trnV‐GAC*) were duplicated in the IR regions (Fig. 1).

Introns play an important role in gene expression regulation. Many introns have the ability to enhance the high expression of exogenous genes at specific times and locations of plants, thus producing the desired agronomic traits. The chloroplast genome of *A. inebrians* includes 15 intron‐containing genes (Table S2). The pseudogene *ycf3* has two introns, while all other genes contain a single intron. The intron of the *trnK*‐UUU gene is largest (2488 bp), and *matK* is located within its intron. The *nadH* gene is a transspliced gene with a 5′ exon located in an SSC region and two 3′ exons located in IR regions, as previously reported in other chloroplast genomes [50, 51].

Nucleotide sequences of protein‐coding genes usually start with ATG. However, there are some exceptions in the *A. inebrians* chloroplast genome in which the first nucleotide is changed from A to G or C, the second nucleotide is changed from T to C, and the third nucleotide is changed from G to C, such as *rps19*, which starts with GTG, *rps12*, starts with ACT, and *rpl2*, starts with ATA (Table S3). This is similar to the common features of many homologous genes reported in the chloroplast genomes of other plants [52, 53, 54, 55, 56, 57, 58].

### Codon usage

**Fig. 2:**
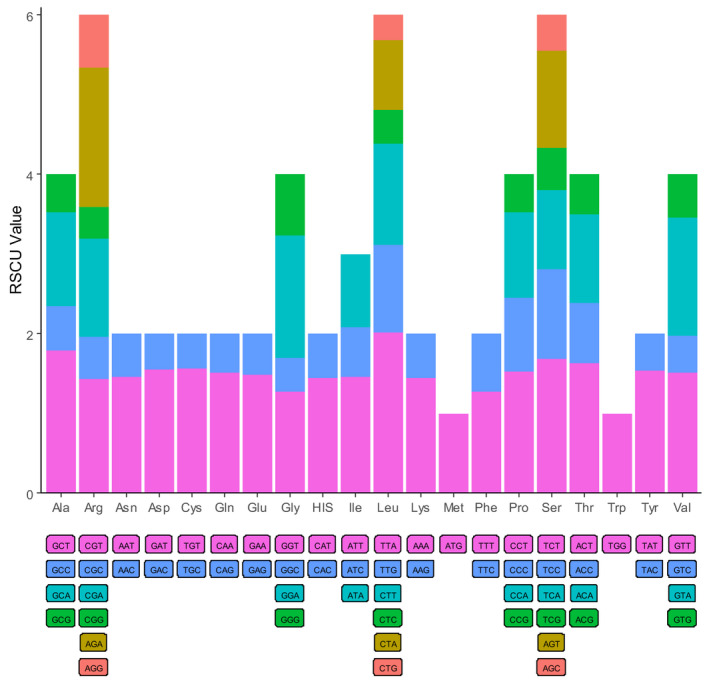
Codon content of 20 amino acids in all protein‐coding genes of the *Achnatherum* *inebrians* chloroplast genome.

**Fig. 3:**
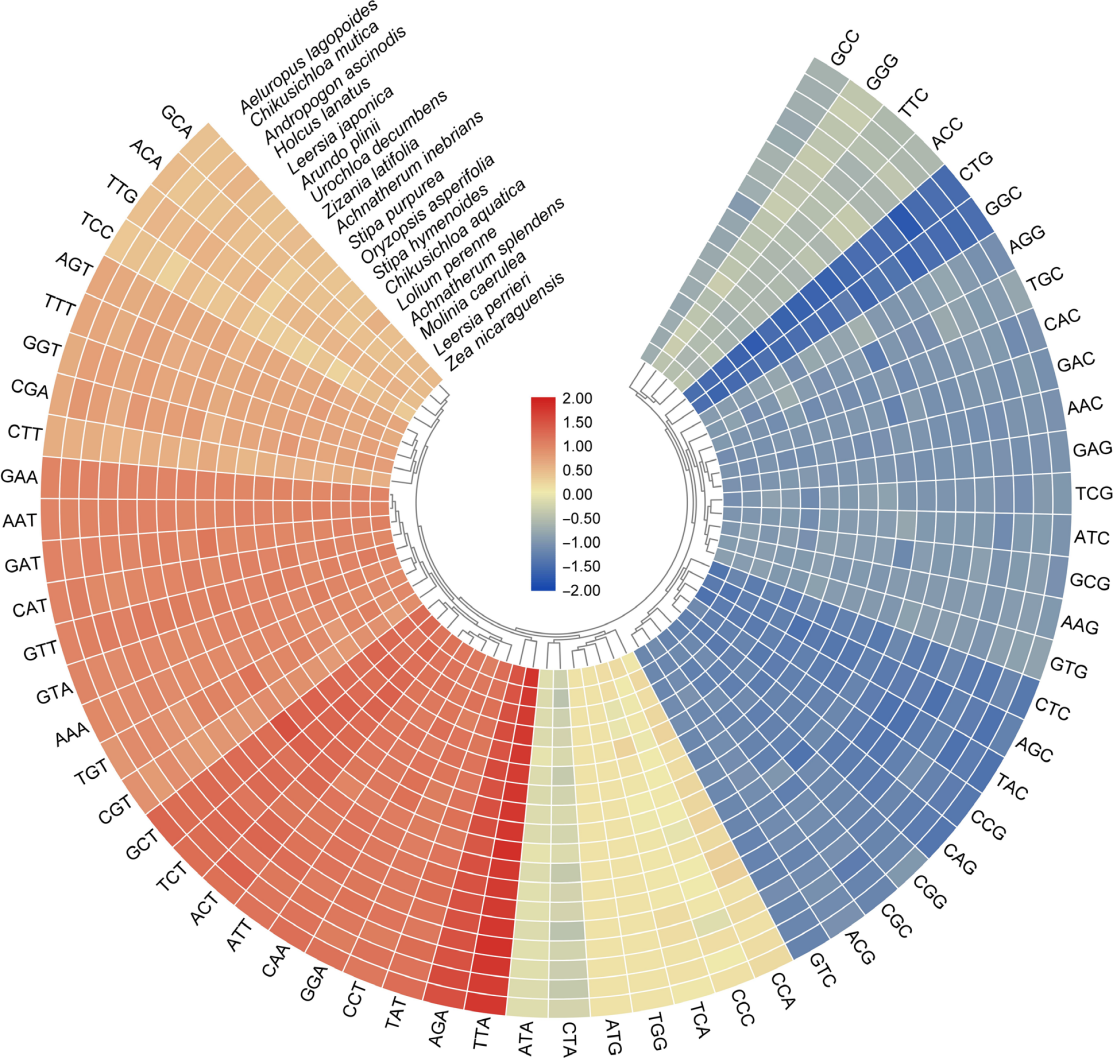
Heatmap analysis for codon distribution of all protein‐coding genes of 18 Poaceae species. Color key: higher red values indicate higher RSCU values, and lower blue values indicate lower RSCU values.

The codon usage frequency and RSCU were analyzed based on the sequences of 84 protein‐coding genes in the *A. inebrians* chloroplast genome (Fig. 2). The highest frequency codon is ATT (leucine), which is the most abundant universal amino acid. The code usage pattern is similar to the reported patterns in other chloroplast genomes, with high A/T content. The codon used in the chloroplast genomes of 18 plants, including *A*. *inebrians*, was compared among all species to better understand the codon preference in Poaceae plants. As shown in Fig. 3, the distributions and the visualization of codon usage in the form of a heatmap of 18 species of Poaceae suggested that approximately one‐third of the codons was not frequently used. These codons are shown in blue, which indicates an RSCU value of less than 1 and weak codon bias. The results showed the codon usage preferences of the most chloroplast genome, among which TTA, AGA, GCT, TCT and ACT are used most frequently (Fig. 3). Approximately two‐thirds of all codons of *A. inebrians* that had high RSCU values showed a high A/T preference in the third codon. This phenomenon is common in the chloroplast genomes of higher plants [59, 60].

### Repeat sequences and SSR analyses

**Fig. 4:**
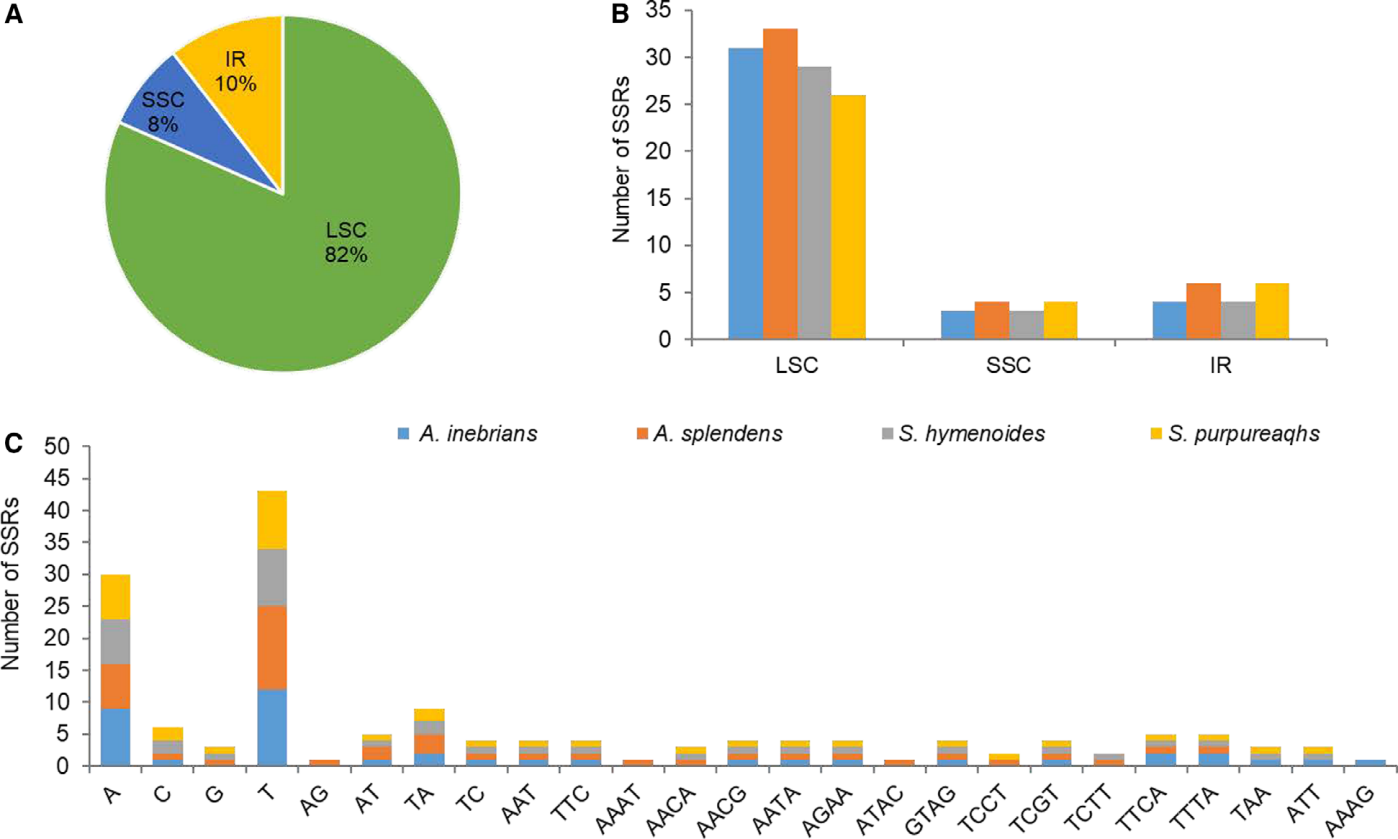
SSR analysis of the four Poaceae chloroplast genomes. (A) Presence of SSRs in the LSC, SSC and IR regions (*A*. *inebrians*). (B) The frequency of SSRs in LSC, IR and SSC regions. (C) The frequency of SSRs of different types.

SSRs, also known as microsatellites, a section of DNA in a genome consisting of the basic units of one to six and repeated many times, are widely distributed in chloroplast genomes. SSRs are often used as molecular markers for studying chloroplast genome evolution and population genetics [61, 62]. We investigated the distribution of SSRs in the *A. inebrians* chloroplast genome and found a total 38 SSRs, of which 31 were in the LSC region (82%), 3 were in the SSC region (8%) and 4 were in IR regions (10%; Fig. 4A). In total, four categories of SSRs, that is, mononucleotide, dinucleotide, trinucleotide and tetranucleotide, were detected. Mononucleotide repetition is most prevalent in each chloroplast genome, followed by dinucleotide, trinucleotide and tetranucleotide repetition. The most dominant SSRs are A/T mononucleotides (18%) from the frequency of the classified repeat types (Table S4). The SSR motifs in the *A. inebrians* and three other chloroplast genomes (*A*. *splendens*, *Stipa* *hymenoides*, *Stipa* *purpurea*) that are closely related to *A. inebrians* were analyzed (Fig. 4B). The study results showed little differences in the distribution pattern and number of SSRs among the four chloroplast genomes except the tetranucleotide repetition AAAG, which was detected in only *A. inebrians* (Fig. 4C).

### Comparative genome analyses

**Fig. 5:**
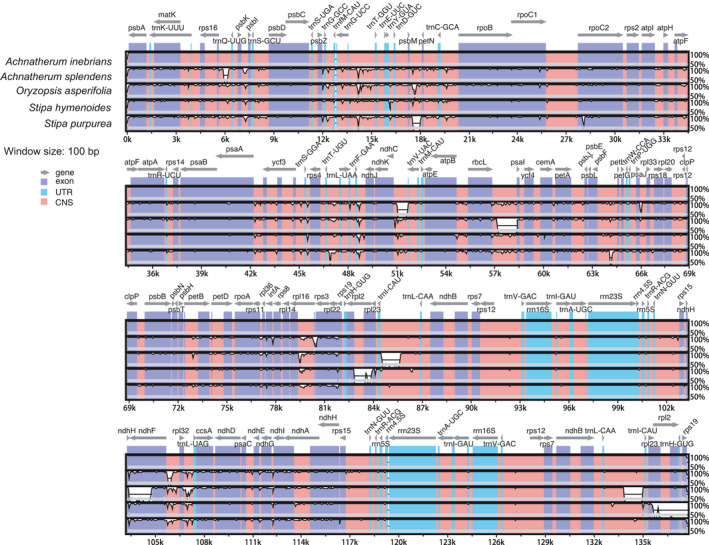
Sequence alignment of five Poaceae genomes in mvista. The *x* axis represents the coordinates in the chloroplast genome. The vertical scale indicates the identity percentage, ranging from 50% to 100%.

In this study, the chloroplast genomes of eight Poaceae were analyzed using the mvista program, with *S. hymenoides* serving as a reference (Fig. 5). These species have considerable similarities in genome composition and size. The coding regions of the eight Poaceae species were almost identical, whereas the noncoding regions were more variable. The highly divergent regions were found among the intergenic spacers, including *matk‐rps16*, *rps16‐trnQ‐UGG*, *trnG‐UGG‐trnT‐GGU*, *psbM‐petN*, *rbcl‐psal*, *ndhF‐rpl32*, *rps2‐rpl23* and *psbE‐petL* in LSC, and *ndhF‐rpl32* and *psaC‐ndhE* in SSC, which might be regarded as potential molecular markers for Poaceae plants. In the whole chloroplast variable region, the *A. inebrians* share high sequence identity with those of *S*. *purpurea* more than *A*. *splendens* and relatively lower identity with those of *Cynosurus* *cristatus* and *A. japonicus*.

**Fig. 6:**
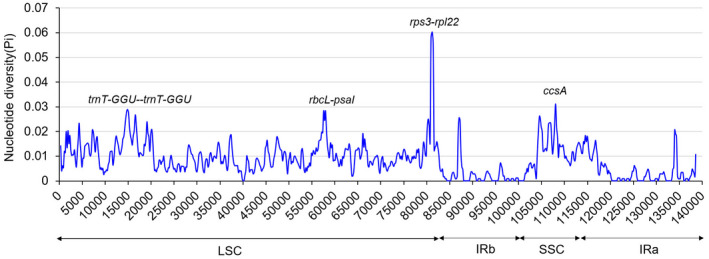
Sliding window analysis of nucleotide variability among the chloroplast genomes of four species (window length: 600 bp; step size: 200 bp).

Pis of four Poaceae were calculated to further demonstrate the differences in the chloroplast genomes of Gramineae at the sequence level. As shown in Fig. 6, the divergence values among *S. purpurea*, *S*. *hymenoides*, *A*. *splendens* and *A*. *inebrians* ranged from 0 to 0.06, with a mean of 0.00837, and the IR regions were more conserved than the LSC and SSC regions. The most divergent region, *rps3‐rpl22*, showed a divergence value of 0.06 in the LSC region, while the *ccsA* gene showed a high Pi (0.031) value in the SSC region. The intergenic regions among *trnT‐GGU‐trnT‐GGU* and *rbcL‐psaI* also showed a relatively high divergence value (>0.025). These regions may undergo rapid nucleotide replacement at the species level. These hotspots can be used as molecular markers and barcodes for phylogenetic analysis and species identification of Poaceae.

**Fig. 7:**
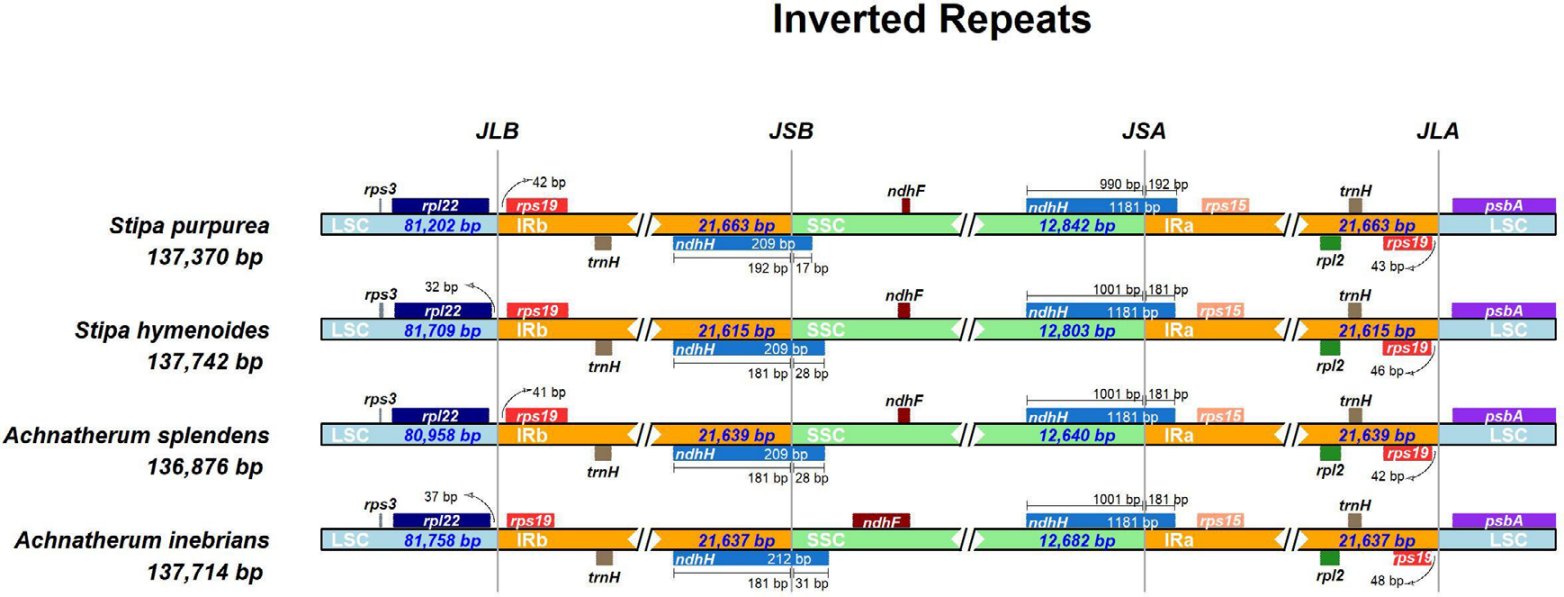
Comparison of the junction positions between the LSC, SSC and IR regions among the chloroplast genomes of four species.

Expansion and contraction at the borders of the IR regions are common evolutionary events that often result in genome size variations in chloroplast genomes. We investigated the position of genes at the junction regions of four chloroplast genomes: *S*. *purpurea*, *S. hymenoides*, *A*. *splendens* and *A*. *inebrians*. In the *A. inebrians* plastome, the boundary of IR–LSC extended into the *rps19* gene; the boundary of IR–SSC extended into the *ndhF* gene, and 48 bp of *ndhF* extended into the IR region a (IRa); and the boundaries of IRs region b (IRb)–LSC and IRa–LSC extend into the *rpl22* and *psbA* genes, respectively. Only 37 bp of *rps22* was duplicated in the LSC region, while 48 bp of *rps19* was duplicated in IRb. Similarly, the *ndhH* gene was located at the junction of SSC–IRa, and *ndhH* is 17, 28, 28 and 31 bp from the SSC and IRb borders in *S. purpurea*, *S. hymenoides*, *A*. *splendens* and *A*. *inebrians*, respectively. The connections between IR and SSC regions often vary in chloroplast genomes of higher plants and have been commonly reported in previous studies [63, 64]. In this study, a detailed comparison of the borders among the IR, LSC and SSC regions of the four Poaceae chloroplast genomes was explored and is presented in Fig. 7. Our results suggest that the IR–LSC boundary might be conserved among the chloroplast genomes of closely related family species.

### Phylogenetic analysis

**Fig. 8:**
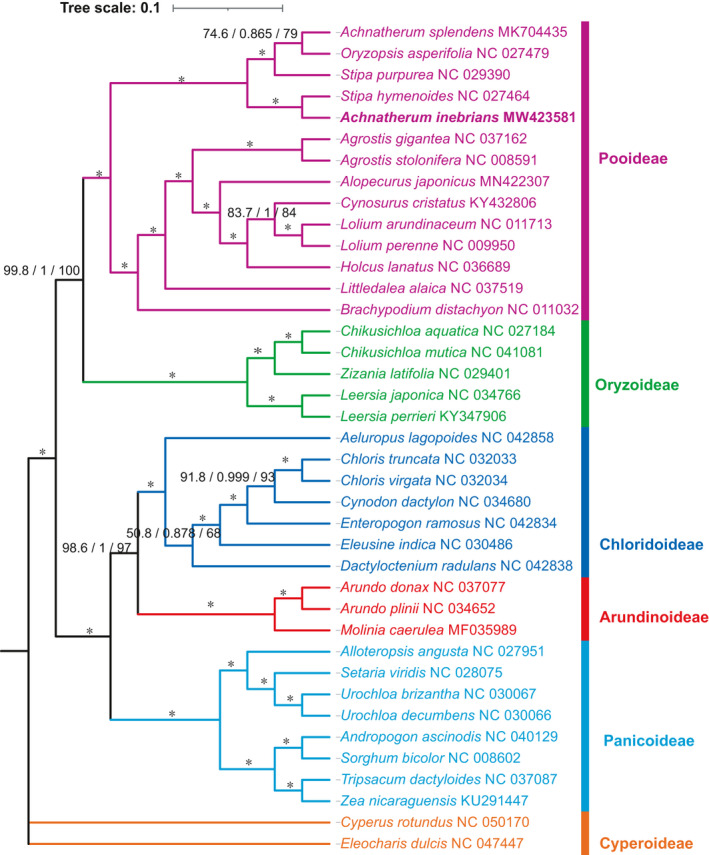
Phylogenetic tree reconstructed from the complete chloroplast genome sequences from 39 species. Statistical support values above the branches correspond to Shimodaira–Hasegawa‐like approximate likelihood‐ratio test (SH‐aLRT) values/approximate Bayes probabilities/ML bootstrap values. Asterisks (*) indicate branches with maximum values of the indices, except where noted.

The phylogenetic tree was constructed based on 37 whole‐chloroplast genomes from the Poaceae family using *C. rotundus* and *E. dulcis* as outgroups (Fig. 8). The phylogenetic trees generated by BI (Fig. S1) and ML methods and their topology were nearly identical. The tree topology from ML analysis is shown in Fig. 8. The relevant data of phylogenetic trees are shown in the supplementary materials (Tables S5 and S6). According to the trees’ topology, the 37 species of Poaceae were divided into five subfamilies: Pooideae, Oryzoideae, Chloridoideae, Arundinoideae and Panicoideae. The ML (bootstraps value = 100) and BI (posterior probability values = 1) topology both supported that *A. inebrians* has a sister relationship to the genus *S. hymenoides*. The position of *A. inebrians* and all other nodes in the topology are supported with posterior probability values of 1.0, except three nodes. Our study provides valuable genetic information for genome‐scale phylogenetic studies in Poaceae plants.

## Discussion

In this study, next‐generation sequencing technology was used to sequence the chloroplast genome of *A. inebrians*, and its genetic information was reported for the first time. The comparative analysis of gene composition and structure revealed that *A. inebrians* has a conserved chloroplast genome like other grassland plants [65, 66].

A total of 130 genes were found in the *A. inebrians* chloroplast genome, including 84 protein‐coding genes, 38 tRNA genes and 8 rRNA genes. The *ycf1*, *ycf2* and *accD* were lost, which is a common trend in many Poaceae plants [67], indicating that genetic degeneration occurred during the process of gene evolution.

A total of 38 SSRs were identified in the *A. inebrians* chloroplast genome. The most dominant SSRs were A/T mononucleotides (18%) from the frequency of classified repeat types. SSRs can be regarded as good markers in plant populations for addressing genetic diversity among closely related taxa. Therefore, improved ability to study interspecies differences can be used in conjunction with SSR markers developed by nuclear genomes to address phylogenetic relationships among closely related species [68].

During the genome evolution process, the sequence marginal region of the IR region was changed [69]. With the expansion and contraction of the IR boundary, some genes entered the IR region and some entered the single‐copy region, resulting in changes in the number of genes among different species. The chloroplast genome size is mainly dependent on the expansion and contraction of IR and SSC boundary regions [70].

The comparative analysis of *A. inebrians* and other species showed that, except for the high conservation of complete chloroplast, there are some significant differences among them. For example, the mvista program and Pi analysis both determined that *rbcl‐psal* and *psbE‐petL* can be used for the development of phylogenetic markers. *A. inebrians* share high sequence identity with those of *S. purpurea* more than *A. splendens* and the same as phylogenetic tree. It is a major finding and will be helpful for researchers in getting more information about genetic resources.

Phylogenetic studies of plants mainly use the chloroplast and nuclear genome to analyze the genome structure and modifications [66, 70]. The Poaceae family not only has an economic importance but also it is one of the major families on which international cooperative molecular phylogenetic studies were conducted [71, 72]. Our results support Poaceae being composed by two big clades: BOP (Bambusoideae, Oryzoideae, and Pooideae) and PACCAD (Panicoideae, Aristidoideae, Chloridoideae, Micrairoideae, Arundinoideae, and Danthonioidea), which is similar to the findings reported in previous research [72, 73]. In this study, for the first time, we reconstructed phylogenetic trees based on the chloroplast genome of 37 Poaceae plants, including *A. inebrians*. In terms of evolutionary relationships, our study results strongly support that *A. inebrians* belongs to the genus *Stipa*.

As for the division and classification of *Achnatherum*, there is an unavoidable relationship between it and *Stipa*. In the past, many scholars did not recognize or use the genus *Achnatherum* and still used *Stipa* in their studies [74, 75, 76, 77]. But at the same time, other scholars used *Achnatherum* in their studies [3, 78, 79, 80]. According to the comparison of the morphological characteristics (Table S7), *A. inebrians* is inclined to the *Achnatherum*, but there are some (awn, fruit, basal disc) morphologically similar to *Stipa*. Our study provides support only for relevant classification at the molecular level and does not fully represent the real classification status. Specific follow‐up studies can make use of mitochondrial genes, nuclear genes and other genetic markers for further classification.

## Conflict of interest

The authors declare no conflict of interest. The funders had no role in the design of the study; in the collection, analyses or interpretation of data; in the writing of the manuscript; or in the decision to publish the results.

## Author contributions

XW, XL and CL designed experiments. XW, ZC and YJ carried out the experiments. XW and ZC analyzed experimental results. XW, TC and KM wrote the manuscript.

## Supporting information


**Fig. S1**. Phylogenetic tree generated by BI.

**Table S1**. All information of species and the accession numbers of their chloroplast genomes in NCBI.

**Table S2**. List of intron‐containing genes in the CP genomes of *Achnatherum* *inebrians*.

**Table S3**. Nucleotide sequences of protein‐coding genes of *Achnatherum* *inebrians* chloroplast genome.

**Table S4**. Frequency of classified repeat types (considering sequence complementary).

**Table S5**. The relevant data of phylogenetic tree generated by BI.

**Table S6**. The relevant data of phylogenetic tree generated by maximum likelihood.

**Table S7**. The morphological characteristics of *Achnatherum* *inebrians*, genus *Achnatherum*, genus *Stipa*.

## Data Availability

The annotated complete chloroplast genome sequences were submitted to the NCBI database with accession number MW423581.
