# Supplementary figures and images for: Complete chloroplast genomes of Achnatherum inebrians and comparative analyses with related species from Poaceae
Source: FEBS Open Bio. 2021 May 10;11(6):1704–18. doi: 10.1002/2211-5463.13170 (PMC8167873; doi:10.1002/2211-5463.13170)

Tree scale: 0.01

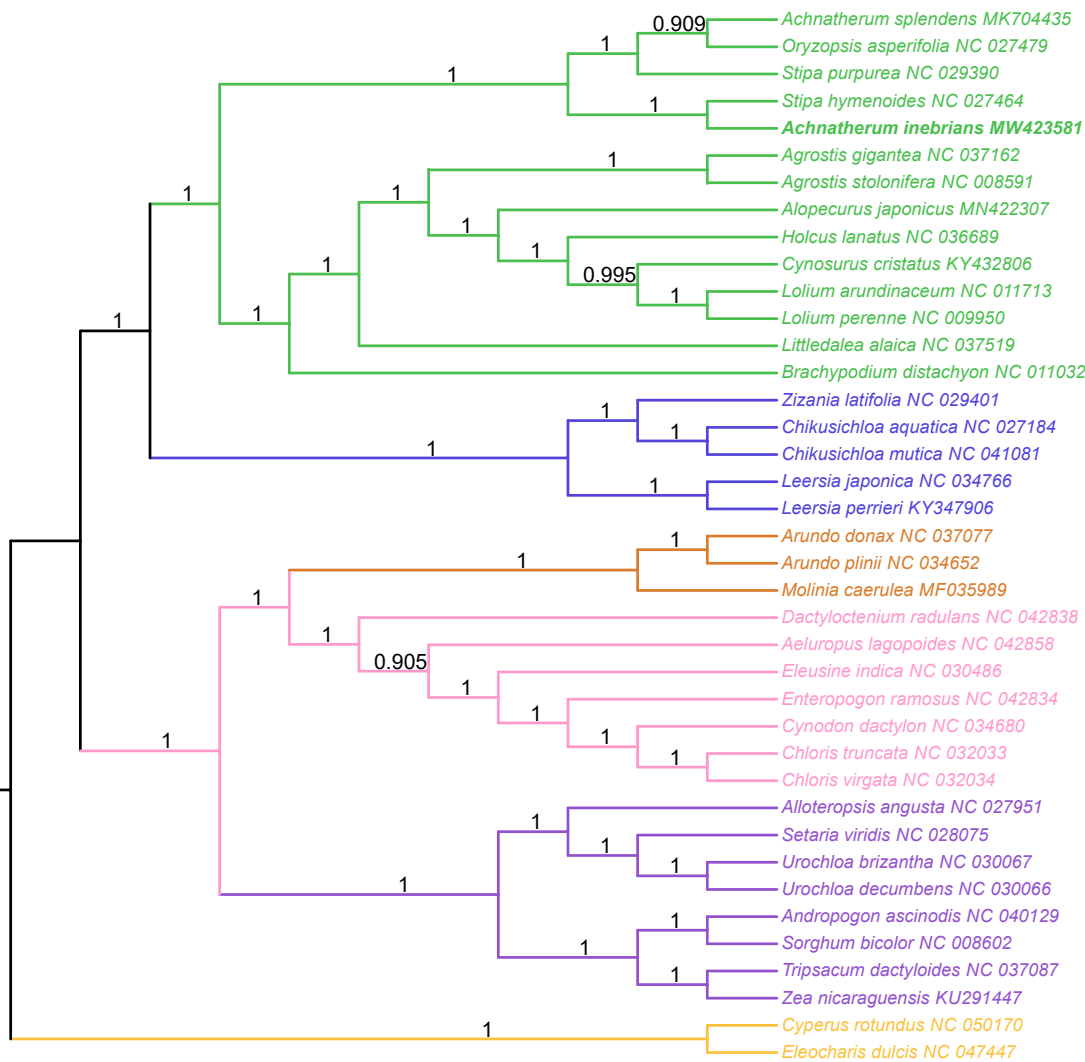

Supplement: Supplementary file 1 — Fig. S1. Phylogenetic tree generated by BI. [file FEB4-11-1704-s001.pdf]
