# Supplementary material for: Complete chloroplast genomes of Achnatherum inebrians and comparative analyses with related species from Poaceae
Source: FEBS Open Bio. 2021 May 10;11(6):1704–18. doi: 10.1002/2211-5463.13170 (PMC8167873; doi:10.1002/2211-5463.13170)
Supplement: Supplementary file 2 — Table S1. All information of species and the accession numbers of their chloroplast genomes in NCBI. [file FEB4-11-1704-s008.docx]

**Table S1** All information of species and the accession numbers of their chloroplast genomes in NCBI

| ID | Organism | Family | Subfamily | Genus | Full length (bp) | A (%) | T (%) | C (%) | G (%) | A+T (%) | G+C (%) | AT skew | GC skew |
| --- | --- | --- | --- | --- | --- | --- | --- | --- | --- | --- | --- | --- | --- |
| KU291447 | *Zea nicaraguensis* | Poaceae | Panicoideae | Zea | 140465 | 30.8 | 30.6 | 19.2 | 19.3 | 61.4 | 38.5 | 0.003 | 0.003 |
| KY347906 | *Leersia perrieri* | Poaceae | Oryzoideae | Leersia | 136196 | 30.7 | 30.4 | 19.4 | 19.5 | 61.1 | 38.9 | 0.005 | 0.004 |
| KY432806 | *Cynosurus cristatus* | Poaceae | Pooideae | Cynosurus | 134799 | 31 | 30.8 | 19.1 | 19.2 | 61.8 | 38.3 | 0.004 | 0.002 |
| MF035989 | *Molinia caerulea* | Poaceae | Arundinoideae | Molinia | 137609 | 30.7 | 30.6 | 19.3 | 19.4 | 61.3 | 38.7 | 0.002 | 0.003 |
| MK704435 | *Achnatherum splendens* | Poaceae | Pooideae | Achnatherum | 136876 | 30.6 | 30.5 | 19.4 | 19.5 | 61.1 | 38.9 | 0.003 | 0.002 |
| MN422307 | *Alopecurus japonicus* | Poaceae | Pooideae | Alopecurus | 136408 | 31 | 30.7 | 19.1 | 19.2 | 61.7 | 38.3 | 0.004 | 0.001 |
| NC_008591 | *Agrostis stolonifera* | Poaceae | Pooideae | Agrostis | 136584 | 30.9 | 30.6 | 19.2 | 19.3 | 61.5 | 38.5 | 0.005 | 0.003 |
| NC_008602 | *Sorghum bicolor* | Poaceae | Panicoideae | Sorghum | 140754 | 30.8 | 30.7 | 19.2 | 19.3 | 61.5 | 38.5 | 0.001 | 0.001 |
| NC_009950 | *Lolium perenne* | Poaceae | Pooideae | Lolium | 135282 | 31 | 30.8 | 19.1 | 19.1 | 61.8 | 38.2 | 0.004 | 0.001 |
| NC_011032 | *Brachypodium distachyon* | Poaceae | Pooideae | Brachypodium | 135199 | 30.8 | 30.6 | 19.2 | 19.3 | 61.4 | 38.5 | 0.004 | 0.003 |
| NC_011713 | *Lolium arundinaceum* | Poaceae | Pooideae | Lolium | 136048 | 31 | 30.6 | 19.2 | 19.2 | 61.6 | 38.4 | 0.007 | 0 |
| NC_027184 | *Chikusichloa aquatica* | Poaceae | Oryzoideae | Chikusichloa | 136563 | 30.6 | 30.3 | 19.4 | 19.6 | 60.9 | 39 | 0.004 | 0.004 |
| NC_027464 | *Stipa hymenoides* | Poaceae | Pooideae | Stipa | 137742 | 30.7 | 30.5 | 19.3 | 19.5 | 61.2 | 38.8 | 0.003 | 0.003 |
| NC_027951 | *Alloteropsis angusta* | Poaceae | Panicoideae | Alloteropsis | 140709 | 30.9 | 30.7 | 19.2 | 19.3 | 61.6 | 38.5 | 0.003 | 0.003 |
| NC_028075 | *Setaria viridis* | Poaceae | Panicoideae | Setaria | 138102 | 30.7 | 30.6 | 19.3 | 19.4 | 61.3 | 38.7 | 0.003 | 0.004 |
| NC_029390 | *Stipa purpurea* | Poaceae | Pooideae | Stipa | 137370 | 30.7 | 30.5 | 19.3 | 19.5 | 61.2 | 38.8 | 0.003 | 0.003 |
| NC_029401 | *Zizania latifolia* | Poaceae | Oryzoideae | Zizania | 136461 | 30.6 | 30.4 | 19.4 | 19.6 | 61 | 39 | 0.005 | 0.004 |
| NC_030066 | *Urochloa decumbens* | Poaceae | Panicoideae | Urochloa | 138945 | 30.8 | 30.6 | 19.2 | 19.4 | 61.4 | 38.6 | 0.003 | 0.004 |
| NC_030067 | *Urochloa brizantha* | Poaceae | Panicoideae | Urochloa | 138946 | 30.8 | 30.6 | 19.2 | 19.4 | 61.4 | 38.6 | 0.002 | 0.004 |
| NC_030486 | *Eleusine indica* | Poaceae | Chloridoideae | Eleusine | 135151 | 31 | 30.8 | 19 | 19.2 | 61.8 | 38.2 | 0.003 | 0.003 |
| NC_032033 | *Chloris truncata* | Poaceae | Chloridoideae | Chloris | 135584 | 31 | 30.8 | 19.1 | 19.1 | 61.8 | 38.2 | 0.004 | 0 |
| NC_032034 | *Chloris virgata* | Poaceae | Chloridoideae | Chloris | 134561 | 31 | 30.7 | 19.1 | 19.2 | 61.7 | 38.3 | 0.004 | 0.002 |
| NC_034652 | *Arundo plinii* | Poaceae | Arundinoideae | Arundo | 137128 | 30.8 | 30.6 | 19.3 | 19.4 | 61.4 | 38.7 | 0.003 | 0.003 |
| NC_034680 | *Cynodon dactylon* | Poaceae | Chloridoideae | Cynodon | 134297 | 30.9 | 30.7 | 19.1 | 19.2 | 61.6 | 38.3 | 0.003 | 0.003 |
| NC_034766 | *Leersia japonica* | Poaceae | Oryzoideae | Leersia | 134074 | 30.7 | 30.4 | 19.4 | 19.5 | 61.1 | 38.9 | 0.005 | 0.004 |
| NC_036689 | *Holcus lanatus* | Poaceae | Pooideae | Holcus | 135668 | 30.9 | 30.7 | 19.2 | 19.2 | 61.6 | 38.4 | 0.003 | 0.001 |
| NC_037077 | *Arundo donax* | Poaceae | Arundinoideae | Arundo | 137093 | 30.8 | 30.6 | 19.3 | 19.4 | 61.4 | 38.7 | 0.003 | 0.002 |
| NC_037087 | *Tripsacum dactyloides* | Poaceae | Panicoideae | Tripsacum | 141050 | 30.8 | 30.7 | 19.2 | 19.3 | 61.5 | 38.5 | 0.002 | 0.003 |
| NC_037162 | *Agrostis gigantea* | Poaceae | Pooideae | Agrostis | 136705 | 30.9 | 30.6 | 19.2 | 19.3 | 61.5 | 38.5 | 0.005 | 0.003 |
| NC_037519 | *Littledalea alaica* | Poaceae | Pooideae | Littledalea | 136840 | 30.8 | 30.7 | 19.2 | 19.3 | 61.5 | 38.5 | 0.003 | 0.002 |
| NC_040129 | *Andropogon ascinodis* | Poaceae | Panicoideae | Andropogon | 138841 | 30.8 | 30.7 | 19.2 | 19.4 | 61.5 | 38.6 | 0.001 | 0.004 |
| NC_041081 | *Chikusichloa mutica* | Poaceae | Oryzoideae | Chikusichloa | 136603 | 30.6 | 30.3 | 19.4 | 19.6 | 60.9 | 39 | 0.005 | 0.004 |
| NC_042834 | *Enteropogon ramosus* | Poaceae | Chloridoideae | Enteropogon | 132690 | 30.9 | 30.7 | 19.1 | 19.2 | 61.6 | 38.3 | 0.004 | 0.003 |
| NC_042838 | *Dactyloctenium radulans* | Poaceae | Chloridoideae | Dactyloctenium | 135050 | 30.9 | 30.7 | 19.1 | 19.3 | 61.6 | 38.4 | 0.004 | 0.003 |
| NC_042858 | *Aeluropus lagopoides* | Poaceae | Chloridoideae | Aeluropus | 135518 | 31 | 30.8 | 19.1 | 19.2 | 61.8 | 38.3 | 0.003 | 0.003 |
| NC_047447 | *Eleocharis dulcis* | Cyperaceae | Cyperoideae | Eleocharis | 199561 | 33.5 | 33.8 | 16.7 | 16 | 67.3 | 32.7 | -0.004 | -0.021 |
| NC_050170 | Cyperus rotundus | Cyperaceae | Cyperoideae | Cyperus | 186119 | 33.2 | 33.6 | 17 | 16.2 | 66.8 | 33.2 | -0.007 | -0.022 |
| MW423581 | Achnatherum inebrians | Poaceae | Pooideae | Achnatherum | 137714 | 30.7 | 30.5 | 19.3 | 19.4 | 61.2 | 38.7 | 0.003 | 0.003 |
