# Supplementary material for: Complete chloroplast genomes of Achnatherum inebrians and comparative analyses with related species from Poaceae
Source: FEBS Open Bio. 2021 May 10;11(6):1704–18. doi: 10.1002/2211-5463.13170 (PMC8167873; doi:10.1002/2211-5463.13170)
Supplement: Supplementary file 3 — Table S2. List of intron‐containing genes in the CP genomes of Achnatherum inebrians. [file FEB4-11-1704-s004.docx]

**Table S2** List of intron-containing genes in the CP genomes of *A. inebrians*

| No. | Gene | Location |  | length |
| --- | --- | --- | --- | --- |
| 1 | atpF | LSC |  | 830 |
| 2 | ndhB | IR |  | 711 |
| 3 | ndhA | SSC |  | 1032 |
| 4 | rpl2 | IR |  | 662 |
| 5 | Rpl16 | LSC |  | 1443 |
| 6 | rps12 | IR |  | 539 |
| 7 | rps16 | LSC |  | 820 |
| 8 | ycf3 | LSC | Intron1 | 701 |
| 9  10 | petD  petB | LSC  LSC | Intron2 | 739  749  820 |
| 11 | trnA-UGC | IR |  | 810 |
| 12 | trnI-GAU | IR |  | 805 |
| 13 | trnK-UUU | LSC |  | 2488 |
| 14 | trnL-UAA | LSC |  | 523 |
| 15 | trnV-UAC | LSC |  | 596 |
