# Supplementary material for: Complete chloroplast genomes of Achnatherum inebrians and comparative analyses with related species from Poaceae
Source: FEBS Open Bio. 2021 May 10;11(6):1704–18. doi: 10.1002/2211-5463.13170 (PMC8167873; doi:10.1002/2211-5463.13170)
Supplement: Supplementary file 4 — Table S3. Nucleotide sequences of protein‐coding genes of Achnatherum inebrians chloroplast genome. [file FEB4-11-1704-s006.docx]

**Table S3** Nucleotide sequences of protein-coding genes of *A. inebrians* chloroplast genome

>rps12

ATGCCAACGGTTAAACAACTTATTAGAAACGCAAGACAGCCAATACGAAATGCTAGAAAATCGGCCGCGCTTAAGGGATGTCCTCAGCGTCGAGGAACATGTGCTAGGGTGTATACTATCAACCCCAAAAAACCCAACTCTGCCTTACGTAAAGTTGCCAGAGTACGATTAACCTCTGGATTTGAAATCACTGCTTATATACCTGGTATTGGCCATAATTTACAAGAACATTCTGTAGTATTAGTAAGAGGAGGAAGGGTTAAGGATTTACCCGGTGTGAGATATCGCATTATTCGAGGAACCCTAGATGCTGTCGCAGTAAAGAATCGTCAACAAGGGCGTTCTAAATATGGGGTCAAAAAGCCAAAAAAATAA

>psbA

ATGACTGCAATTTTAGAGAGACGCGAAAGTACAAGCCTGTGGGGTCGCTTCTGCAACTGGATAACTAGCACCGAAAATCGTCTTTACATCGGATGGTTCGGTGTTTTGATGATCCCTACCTTATTGACCGCAACTTCCGTATTTATTATCGCCTTCATCGCTGCCCCTCCAGTAGATATTGATGGTATTCGTGAGCCTGTTTCTGGTTCTTTACTTTATGGAAACAATATTATCTCTGGTGCCATTATTCCTACTTCTGCGGCGATCGGATTGCACTTTTACCCAATTTGGGAAGCTGCATCTGTTGATGAGTGGTTATACAACGGTGGTCCTTATGAGCTAATTGTTCTACACTTCTTACTTGGTGTAGCTTGTTATATGGGTCGTGAGTGGGAACTTAGTTTCCGTCTGGGTATGCGTCCTTGGATTGCTGTTGCATATTCAGCTCCTGTTGCGGCTGCTACTGCTGTTTTCTTGATTTACCCTATTGGTCAAGGAAGCTTCTCTGATGGTATGCCTTTAGGAATTTCTGGTACTTTCAACTTTATGATTGTATTCCAGGCAGAGCACAACATCCTTATGCATCCATTTCACATGTTAGGTGTAGCTGGTGTTTTCGGCGGTTCCCTATTCAGTGCTATGCATGGTTCCTTGGTAACCTCTAGTTTGATCAGGGAAACTACTGAAAATGAATCTGCTAATGAGGGTTACAGATTTGGTCAAGAGGAAGAGACTTATAATATCGTGGCTGCTCATGGTTATTTTGGCCGATTAATCTTCCAATATGCTAGTTTCAACAACTCTCGTTCTTTACACTTCTTCTTGGCTGCTTGGCCTGTAGTAGGGATCTGGTTCACTGCTTTAGGTATTAGTACTATGGCTTTCAACCTAAATGGGTTCAATTTCAACCAATCTGTAGTTGATAGTCAAGGTCGCGTTATTAATACTTGGGCTGATATCATCAACCGTGCTAATCTTGGTATGGAAGTAATGCACGAACGTAATGCTCACAACTTCCCTCTAGACTTAGCTGCTCTTGAAGTTCCATCTCTTAACGGGTAA

>matK

ATGGAAAAATTCGAAGGGTATTCAGAAAAACTTAAATCTCGTCAACAATACTTCGTCTACCCACTTCTCTTTCAAGAGTATATTTATGCATTTGCCCATGATTATGGATTAAATGGTTCCGAACCTGTGGAAATTGTTAGTTGTAATAACAAGAAATTTAGTTCACTACTTGTGAAACGTTTAATTATTCGAATGTATCAGCAGAATTTTTGGATTAACTCGGTTAATCATCCTAACCAAGATCGATTGTTGGATTACAAAAATTATTTTTATTCTGAGTTTTATTCTCAGATTCTATCTGAGGGGTTTGCGATCGTTGTAGAAATCCCATTCTCGCTACGAGAATTATCTCGTCCGAAAGAAAAAGAAATACAAAAGTTTCAGAATTTACGCTCTATTCATTCAATATTTCCCTTTTTAGAAGACAAATTTTTGCATTTGGATTATCTATCACATATAGAAATACCCTATCCTATCCATTTGGAAATCTTGGTTCAACTCCTTCAATACCATATCCAAGATGTTCCATCTTTGCATTTATTGCGATTCTTTCTCAACTACTATTCGAATTGGAATACTCTTATTACTTCAATGAAATCGATTTTTCTTTTGAAAAAAGAAAATAAAAGACTATTTCGATTCCTATACAACTCTTATGTATCAGAATATGAATTTTTCTTGTTGCTTCTTCGTAAACAATCTTCTTGGTTATCATTAACATCTTCTGGAACCTTTCTAGAACGAATCCACTTTTCTAGGAAGATGGAACATTTTGGGATAATGTACCCAGGTTTTTTTCGGAAAACAGTATGGTTCTTTATGGATCCTCTTATGCATTATGTTCGATATCAAGGAAAGGCAATTCTTGCATCAAAAGGAACTCTTTTTTTGAAGAAGAAATGGAAATGTTACCTTGTCAATTTCTGGCAATATTCTTTCTCTTTTTGGACTCAACCGCGAAGGATCCATCTAAACCAATTAGCAAACTCTTGCTTCGATTTTCTGGGGTACCTTTCAAGTGTACCAAAAATTACTTTGTTAGTAAGGAATCAAATGCTGGAGAATTCTTTTCTAATAGATACTCAAATGAAAAAATTTGATACCACAGTCCCCGCTACTCCCCTCATTGGATCCTTATCAAAAGCTCAATTTTGTACTGGATCGGGGCATCCCATTAGTAAACCCATTTGGACCGATTTATCAGATTTGGATATTCTTGATCGATTTGGTCGGATATGTAGAAATCTTTTTCATTACCATAGTGGATCTTCGAAAAAACGGACTTTGTATCGGCTAAAGTATATACTTCGACTTTCATGCGCTAGAACTTTAGCTCGTAAACATAAAAGCACGGTACGAACTTTTATGCAACGGTTGGGTTCGGTATTTTTAGAAGAATTTTTTACGGAAGAAGAGCAAGTTTTTTCTTTGATGTTCACCAAAACAACCCTTTTTTCTTTCCGCGGATCACACAGTGAGCGTATTTGGTATTTAGATATTATCCGTATCAACGACTTGTTGGACCCTCTTAATTAA

>rps16

ATGGTAAAACTTCGTTTAAAACGATGTGGTAGAAAGCAACAAGCTGTCTATCGAATCGTTGCAATTGATGTTCGATCTCGAAGAGAAGGAAGAGATCTGCGAAAAGTAGGTTTTTATGATCCGATAAAGAATCAAACTTCTTTAAATGTTCCAGCTATTCTATATTTCCTTGAAAAGGGTGCTCAACCTACAAGAACTGTTTATGATATTTTAAGGAAGGTGGAATTCTTTAAAGAAAAAGAAAGAGCTTTGAGTTAA

>psbK

ATGCCTAATATACTTAGTTTAACCTGTATCTGTTTTAATTCTGTTCTTTATCCGACTTGTTTTTTCTTTGCCAAATTGCCCGAAGCTTATGCTATTTTCAACCCAATCGTGGATGTTATGCCTGTCATACCTCTATTCTTTTTTCTATTAGCCTTTGTTTGGCAAGCTGCTGTAAGTTTTCGATGA

>psbI

ATGCTTACTCTCAAACTTTTTGTTTATACAGTAGTGATATTCTTTGTTTCCCTCTTTATCTTTGGATTCTTATCTAATGACCCAGGACGTAATCCTGGGCGCGAGGAGTAA

>psbD

ATGACTATAGCCCTTGGTAGAGTTCCTAAAGAAGAAAATGATCTATTTGATACTATGGATGACTGGTTACGAAGGGACCGTTTCGTTTTTGTAGGATGGTCTGGCCTATTGCTCTTTCCTTGTGCTTATTTCGCTTTAGGGGGTTGGTTTACAGGGACAACTTTTGTAACTTCTTGGTATACCCATGGATTGGCTAGTTCCTATTTGGAAGGTTGTAATTTCTTAACCGCGGCAGTTTCTACCCCCGCCAATAGTTTAGCACACTCTTTGTTGCTACTATGGGGCCCGGAAGCACAAGGAGATTTTACTCGTTGGTGTCAATTAGGCGGTCTATGGACTTTTGTCGCTCTCCACGGGGCTTTTGCACTAATAGGTTTCATGTTACGCCAATTTGAACTTGCTCGGTCTGTTCAATTGCGGCCTTATAATGCAATCTCATTCTCTGCTCCAATCGCTGTTTTTGTTTCTGTATTCCTTATTTATCCACTGGGTCAATCTGGTTGGTTCTTTGCGCCGAGTTTTGGCGTAGCAGCGATATTTCGATTCATCCTCTTCTTCCAAGGATTTCATAATTGGACGTTGAACCCATTTCATATGATGGGAGTTGCCGGAGTATTAGGCGCAGCTCTGCTATGCGCTATTCATGGGGCGACCGTAGAAAACACTCTATTCGAGGACGGTGATGGTGCAAATACCTTCCGCGCTTTTAACCCAACTCAAGCTGAAGAAACTTATTCAATGGTCACTGCTAACCGCTTTTGGTCCCAAATCTTTGGTGTTGCTTTTTCCAATAAACGTTGGTTACATTTCTTTATGCTATTTGTACCCGTTACCGGTTTATGGATGAGTGCTATTGGCGTAGTTGGCCTGGCTCTGAACTTACGTGCCTATGACTTCGTTTCCCAGGAAATCCGTGCAGCAGAAGATCCTGAATTTGAGACTTTCTACACCAAAAATATTCTTTTAAACGAGGGTATTCGTGCGTGGATGGCAGCTCAGGATCAGCCTCATGAAAATCTTATATTCCCTGAGGAGGTTCTACCACGTGGAAACGCTCTTTAA

>psbC

ATGAAAATCTTATATTCCCTGAGGAGGTTCTACCACGTGGAAACGCTCTTTAATGGAACTTTCGTTTTAGCTGGTCGTGACCAAGAAACCACCGGCTTTGCTTGGTGGGCTGGGAATGCCAGACTTATCAATTTGTCCGGTAAACTACTTGGAGCTCACGTAGCCCATGCCGGATTAATCGTATTCTGGGCCGGAGCAATGAACCTATTTGAAGTGGCCCATTTCGTACCAGAAAAGCCCATGTATGAACAAGGGTTGATTTTACTTCCACACTTAGCTACTCTAGGTTGGGGAGTAGGGCCTGGGGGGGAAGTTCTAGATACTTTTCCGTACTTTGTATCTGGAGTACTTCACCTAATTTCCTCCGCAGTCTTAGGCTTCGGTGGCATTTATCACGCGCTTCTGGGACCCGAGACTCTTGAGGAATCTTTTCCATTCTTTGGTTATGTATGGAAAGATAGAAATAAAATGACTACAATTTTGGGTATTCACTTAATTTTGTTAGGTCTAGGTGCTTTTCTTCTAGTACTCAAGGCTCTTTATTTTGGTGGTGTATATGATACCTGGGCCCCTGGGGGGGGAGATGTAAGAAAAATTACCAATTTGACCCTTAGCCCCAGTGTTATATTTGGTTATTTACTAAAATCCCCTTTTGGGGGAGAAGGGTGGATTGTTAGTGTGGATGATTTAGAAGATATAATTGGTGGACATGTATGGTTGGGTTTCATTTGTGTATTTGGTGGAATTTGGCATATCTTAACCAAACCCTTCGCATGGGCTCGCCGTGCATTTGTATGGTCTGGAGAAGCTTACTTGTCTTATAGTTTAGCTGCTTTATCTGTCTTTGGTTTTATCGCTTGTTGTTTTGTCTGGTTCAATAATACGGCTTATCCGAGTGAGTTTTATGGACCCACTGGGCCAGAAGCTTCTCAAGCTCAAGCATTTACTTTTCTAGTTAGAGACCAGCGTCTTGGAGCTAATGTGGGATCTGCTCAAGGACCCACAGGTTTAGGTAAATATTTAATGCGTTCCCCAACAGGGGAGGTTATCTTTGGAGGGGAAACTATGCGTTTTTGGGACCTTCGTGCTCCATGGTTAGAACCTCTAAGGGGCCCCAACGGTTTGGACTTGAGTAGGTTGAAAAAGGACATACAACCTTGGCAAGAACGACGTTCAGCAGAATATATGACCCATGCTCCTTTAGGCTCTTTAAATTCCGTGGGTGGCGTAGCTACCGAGATCAATGCAGTTAATTATGTCTCTCCTAGAAGTTGGTTATCGACCTCTCATTTTGTTCTAGGATTCTTCTTTTTTGTGGGCCATTTGTGGCATGCAGGAAGAGCCCGAGCTGCTGCAGCAGGTTTTGAAAAGGGAATCGATCGTGATTTGGAACCTGTTCTTTACATGAACCCTCTTAACTAA

>psbZ

ATGACTATTGCTTTCCAATTAGCTGTTTTTGCATTAATTGCGACTTCCTCAGTTTTAGTAATTAGTGTACCCCTTGTATTTGCTTCTCCTGATGGTTGGTCAAATAATAAAAACGTTGTATTTTCCGGTACATCATTATGGATTGGACTAGTCTTTCTCGTAGCTATTCTGAATTCTCTCATTTCTTAA

>psbM

ATGGAAGTCAATATTCTCGCATTTATTGCTACTGCACTGTTCATTCTAATTCCTACTGCCTTTTTACTTATTATTTATGTAAAAACAGTCAGCCAAAATAATTAA

>petN

ATGGATATAGTAAGTCTCGCTTGGGCTGCTTTAATGGTAGTCTTTACATTTTCTCTTTCACTAGTAGTATGGGGGAGGAGTGGACTCTAG

>rpoB

ATGCTCCGGAATGGAAATGAGGGAATGTCCACAATACCCGAATTTAGTCAGATCCAATTCGAGGGATTTTGTAGGTTCATTAATCAAGGCTTGGCAGAAGAACTTGAGAAGTTTCCAACAATTAAAGATCCAGATCACGAAATTGCATTTCAATTATTTGCGAAAGGATATCAATTGCTAGAACCCTCGATAAAAGAAAGGGATGCTGTGTATGAATCACTCACCTATTCTTCCGAATTATATGTATCTGCGAGATTAATTTTTGGTTTCGATGTGCAAAAGCAAACCATTTCTATTGGAAACATTCCTATAATGAATTCCTTAGGAACCTTTATAATAAATGGAATATACCGAATTGTGATCAATCAAATATTGCTAAGTCCTGGTATTTACTACCGCTCGGAATTAGACCATAAGGGAATTTCTATCTACACAGGGACTATAATATCAGATTGGGGAGGAAGATCGGAATTAGCAATTGATAAAAAAGAAAGGATATGGGCTCGTGTGAGTAGAAAACAAAAGATATCTATTCTAGTTCTATCATCAGCTATGGGTTCGAATCTAAGAGAAATTCTAGATAATGTTTCCTACCCTGAAATTTTCTTGTCTTTCCCGAATGCTAAGGAGAAGAAGAGGATTGAGTCAAAAGAAAAAGCTATTTTGGAGTTTTATCAACAATTTGCTTGTGTAGGTGGGGACCTGGTATTTTCGGAGTCCTTATGTGAAGAATTACAAAAGAAATTTTTTCAACAAAAATGTGAATTAGGAAGGATTGGTCGACGAAATATGAATCGGAGACTGAATCTTGATATACCTCAGAACAATACATTCTTGTTACCACGAGATGTATTGGCCGCTACGGATCATTTGATTGGAATGAAATTTGGAACGGGTATACTTGACGATGACGATATGAATCACTTGAAAAATAAACGTATTCGTTCCGTTGCGGATCTGTTACAAGATCAATTCGGGCTGGCTCTTGGTCGTTTACAACATGCAGTTCAAAAAACTATCCGTAGAGTATTCATACGTCAATCGAAACCGACTCCCCAAACTTTGGTAACTCCAACTTCAACTTCGATTTTATTAATAACTACTTATGAGACCTTTTTTGGCACATACCCCTTATCTCAAGTTTTTGATCAAACGAATCCATTGACCCAAACTGTTCATGGGCGAAAAGTTAGTTGTTTGGGTCCTGGAGGGTTGACGGGGAGAACTGCAAGTTTTCGGAGCCGAGATATTCATCCGAGTCACTATGGGCGTATTTGTCCAATTGACACGTCCGAAGGAATCAATGTTGGACTTACAGGATCCTTAGCTATTCATGCGAGAATTGATCACTTGTGGGGATCTGTAGAGAGTCCGTTTTATGAAATATCTGCTGAGAAAGCAAAAGAAAAAAAAGAGAGACAGGTGGTTTATCTATCACCAAATAGAGATGAATATTATATGATAGCAGCAGGAAATTCTTTGTCCTTGAATCAGGGTATTCAGGAAGAGCAGGTTGTTCCTGCTAGATACCGTCAAGAATTCCTGACTATTGCATGGGAACAGATTCATGTTAGAAGTATTTTTCCTTTCCAATATTTTTCTATTGGGGGTTCTCTCATTCCTTTTATTGAGCACAATGATGCGAATCGGGCTTTAATGAGTTCTAATATGCAGCGCCAAGCAGTTCCGCTTTCTCGGTCCGAGAAGTGTATTGTTGGAACTGGATTGGAACGCCAAACAGCTCTAGATTCGAGGGTTTCCGTTATAGCCGAACGCGAGGGAAAGATCATTTCTACTGATAGTCATAAGATCCTTTTATCAAGTAGTGGGAAGACTATAAATATTCCTTTAGTTAACCACCGGCGCTCTAACAAAAATACTTGTATGCACCAAAAACCTCGGGTTCCGCGGGGTAAATCTATTAAAAAAGGACAAATTTTAGCAGAGGGAGCTGCTACAGTTGGGGGGGAACTTGCTTTAGGAAAAAATGTATTAGTAGCTTATATGCCATGGGAAGGTTACAATTTTGAAGACGCAGTACTAATTAGCGAACGTTTGGTATATGAGGATATTTATACCTCTTTTCACATCCGTAAATATGAAATTCAGACGGATACGACAAGCCAAGGCTCCGCTGAAAAAATCACTAAAGAAATACCACATCTAGAAGAACATTTACTCCGCAATTTGGACAGAAATGGAGTTGTTAGGTTGGGATCCTGGGTGGAAACTGGCGATATTTTAGTAGGTAAATTAACGCCTCAGATAGCGAGCGAATCGTCGTATATCGCGGAAGCTGGATTATTACGGGCCATATTTGGCCTTGAGGTATCCACTTCAAAAGAAACATCTCTCAAATTACCTATAGGCGGAAGAGGGCGCGTTATCGATGTGAAATGGATCCAGAGGGACCCCCTCGACATAATGGTTCGTGTATATATTTTACAGAAACGTGAAATCAAAGTTGGGGATAAAGTAGCCGGAAGACATGGGAATAAGGGAATCATTTCCAAAATTTTGCCTAGGCAAGATATGCCCTATTTGCAAGATGGAACACCTGTTGATATGGTCTTCAATCCCTTAGGAGTACCCTCCCGAATGAATGTGGGACAAATATTTGAAAGCTCGCTCGGATTAGCGGGGGATCTGCTAAAGAAACATTATAGAATAGCACCCTTTGATGAGAGATATGAGCAAGAGGCTTCAAGAAAACTTGTGTTTTCAGAATTATATGAAGCCAGTAAACAAACAAAAAATCCATGGGTATTTGAACCCGAGTACCCGGGAAAAAGCAGAATATTTGATGGAAGAACAGGAGACCCCTTCGAACAACCTGTTCTAATAGGGAAGTCCTATATCTTAAAATTAATTCATCAAGTTGATGAGAAAATTCATGGGCGTTCTACTGGGCCCTACTCACTTGTTACACAACAACCTGTTAGAGGAAGAGCCAAGCAAGGGGGACAACGAGTAGGAGAAATGGAAGTTTGGGCTTTAGAAGGATTTGGTGTTGCTCATATTTTACAAGAGATACTTACTTATAAATCTGACCATCTTATAGCTCGCCAAGAAATACTTAATGCTACGATCTGGGGAAAAAGAATACCTAATCACGAGGATCCTCCAGAATCTTTTCGAGTGCTCGTGCGAGAACTACGATCTTTGGCTCTAGAACTGAATCATTTCCTTGTATCTGAAAAGAACTTCCAGGTTAATAGGGAGGAAGTTTGA

>rpoC1

ATGATTGACCAATATAAACATCAACAACTTCAAATTGGACTCGTTTCCCCTCAACAAATAAGGGCTTGGGCTAACAAAAACCTACCTAATGGAGAAGTCGTTGGCGAAGTCACAAGGCCCTCTACTTTTCATTATAAAACCGATAAACCAGAAAAAGATGGATTGTTTTGCGAAAGAATCTTTGGACCCATAAAAAGCGGAATTTGCGCTTGTGGAAATTCTCGAGCGAGCGGAGCTGAAAACGAAGAAGAAAGATTTTGCCAAAAATGCGGGGTAGAATTTGTTGATTCTCGGATACGAAGATATCAAATGGGATACATCAAACTCGCATGTCCCGTGACTCATGTGTGGTATTTGAAAGGTCTTCCTAGTTATATCGCGAATCTTTTAGATAAACCTCTTAAGAAGTTGGAGGGCCTAGTATACGGCGACTTCTCTTTTGCTAGGTCCAGCACTAAAAAACCCACTTTCTTACGATTACGAGGTTTATTCGAAGAGGAAATTTCATCCTGTAACCACAGCATTTCTCCCTTTTTTTCTACCCCTGGGTTTGCAACATTTCGAAATCGGGAAATTGCGACAGGAGCAGGTGCTATTAGAGAACAATTAGCAGATTTGGATTTGCGAATTATTATAGAGAATTCCTTGGTCGAATGGAAGGAATTAGAAGATGAGGGTTATAGTGGAGATGAATGGGAAGATAGAAAAAGACGAATAAGAAAAGTTTTTTTGATTAGACGCATGCAATTGGCGAAACATTTTATTCAAACAAATGTAGAACCAGAATGGATGGTTTTGTGCTTATTACCGGTTCTTCCTCCCGAATTGAGACCCATTGTTTATAGGTCTGGGGATAAAGTAGTGACTTCGGACATTAATGAACTTTATAAGAGAGTTATCCGTCGGAACAACAACCTTGCCTATCTATTAAAAAGAAGTGAATTAGCGCCAGCAGATTTAGTAATGTGCCAGGAAAAATTGGTACAAGAAGCCGTGGATACACTTCTTGATAGTGGGTCTCGCGGGCAACCAACGAGGGATGGTCACAATAAAGTATACAAATCACTTTCAGATGTAATTGAAGGTAAAGAGGGAAGGTTTCGCGAGACTCTGCTTGGGAAACGGGTCGATTACTCTGGGCGTTCTGTCATTGTTGTGGGTCCTTCGCTTTCATTACATCAATGTGGATTACCTCTAGAGATAGCAATAAAGCTTTTTCAGCTATTTGTAATTCGCGATTTAATCACGAAACGCGCTACTTCTAATGTCAGGATTGCTAAAAGGAAAATTTGGGAAAAGGAACCCATTGTATGGGAAATACTTCAAGAAGTTATGCGGGGACACCCTGTACTGTTAAATAGAGCACCTACCCTGCATAGATTAGGCATACAGGCCTTCCAACCCACTTTAGTAGAGGGGCGTACTATTTCTTTACACCCATTAGTGTGTAAGGGTTTCAATGCGGACTTTGATGGGGATCAAATGGCTGTTCATCTACCTTTATCCTTGGAAGCTCAGGCGGAAGCCCGTTTACTTATGTTTTCTCATATGAATCTCTTATCTCCCGCTATTGGAGATCCGATTTGCGTACCAACCCAAGACATGCTTATCGGACTTTATGTATTAACGATTGGAAACCGTCGAGGTATTTGTGCAAATAGATATAATAGTTGCGGAAACTATCCAAATCAAAAAGTAAATTACAATAATAATAATTATAAGTATAATAAGTATACGAAAGATAAAGAACCCTATTTTTCTAGTTCTTATGATGCACTGGGAGCTTATAGACAGAAACTAATCAGTTTAGACAGTCCCTTGTGGCTACGTTGGAAACTAGATCAACGCGTCATTGGGTCAAGAGAAGTTCCGGTTGAAGTTCAATATGAATCTTTGGGGACTTATCATGAGATTTATGCCCACTATTTAATAGTGGGAAATAGAAAAAAAGAAATCCGTTCTATATACATTCGAACCACTCTTGGTCATATTTCTTTTTATAGAGAAATAGAGGAAGCCATACAAGGATTTAGTCAGGCCTATTCATACACTATCTAA

>rpoC2

ATGGGTGTTTTACGTAGAAATACTATTTTTGCTTATTTTGACGATCCACGATACAGAAAAGATAAAAAGGGTTCAGGAATTGTTAAATTTAGATACAGGACCCTAGAGGACGAATATAGGACTCTAGAGGAAGACTCAGAGGACAAATATGAGACCCTAGAAGACGAATATAGGACCCGAGAGGACGAATATGAAACCCTAGAAGAAGATAAATATGGGATCCTAGAGGACGAATATGAAACCCTAGAAGACGAATATGGGAGCCCAGAGAACGAATATGGGAGCCCAGAGAACGAATATAGGATTTTAGAGAAAGACTCAGAAGACGAATATGGGAGCCCGGAGAGCAAATATAGGACCCGAGAGGACGAATATGGAACGCTAGAGGAAGACTCAGAAGACGAATATGGGAGCCCGGGGGAAGGCTCAGAGGACAAATACGGGACTTTAGAGGAAGACTCCGAAGAAGACTCAGAGGACGAATACGAGAGCCCAGAGGAAGATTCCATCTTAAAAAAAGAGGGTTTGATTGAGCATCGAGGAACAAAAGAATTTAGTATAAAATACCAAAAAGAAGTAGATCGGTTTTTTTTCATTCTTCAAGAACTGCATATCTTGCCGAGATCTTCATCCTTAAAAGTACTTGACAATAGTATTATTGGGGTGGATACACAACTCACAAAAAATACAAGAAGTCGACTAGGTGGACTGGTCCGAGTGAAGAGAAAAAAAAGCCATACGGAACTCAAAATAGTTTCCGGAGATATTCATTTTCCTGAAGAGGCGGATACGATATTAGGTGGCTGTTTGGTACCACCAGAAAGACAAAAAAAAGATTCTAAGGAATCAAAAAAAAGGAAAAATTGGGTCTATGTTCAACGGAAAAAAATTCTCAAGAGCAAAGAAAAGTATTTTGTTTCGGTTCGCCCTGCAGTCGCATATGAAATGGACGAAGGGAGAAATTTAGCAACACTTTTCCCGCAGGATCTCTTGCAAGAAGAAGATAATCTCCAACTTCGACTTGTCAATTTTATTTCTCATGAAAATAGCAAGTTAACTCAAAGAATTTATCACACGAATAGTCAATTTGTTAGAACTTGCTTAGTAGTGAATTGGGAACAAGAAGAAAAGGAGGAGGCTCGTGCTTCCCTTGTTGAGGTAAGAGCAAATGATCTTATTCGCGATTTCCTAAGAATTGAGTTAGTCAAGTCCACTATTTCGTATACACGAAGAAGGTATGATAAGACAAGTGCAGGACCGATTCCCCATAATAGGTTAGATCGCACCAATAGCAATTCCTTTTATTCCAAGGCGAAGATTGAATCACTTAGCCAACATCAAGAAGCTATTGGCACCTTGTTGAATCGAAATAAAGAATACCAATCTTTGATGATTTTGTCGGCATCCAACTGTTCTCGAATTGGTTTATTCAAGAATTCAAAACATCCCAATGCCATAAAAGAATCGACTCCTAGAATTCCTATTCGAGAGATTTTTGGGCCTTTAGGCGCTATTGTACCTAGTATATCGAATTTTTCTTCATCTTACTATTTACTAACGCATAATCAGATCCTGTTAAAAAAATATTTGTTCCTTGACAATTTGAAACAAACCTTCCAAGTACTTCAAGGACTTAAATACTCTTTAATAGATGAAAATCAAAGGATTTCAAATTTCGATAGTAACATCATGTTGGATCCATTCTATTTGAATTGCCACTTTGTCCATCATGATTCTTGGGAGGAGACATCGGCAATAATTCACCTTGGACAATTTATTTGTGAAAATGTATGTCTATTTAAATCGCACATAAAAAAATCTGGTCAAATTTTCATTGTTAATATGGATTCCTTTGTTATAAGAGCAGCTAAGCCTTATTTGGCCACTACAGGAGCAACTGTTAATGGTCATTATGGAGAAATCCTTTACAAAGGAGATAGGTTAGTTACGTTTATATATGAAAAATCGAGATCTAGTGACATAACGCAAGGTCTTCCAAAAGTGGAACAAATCTTTGAAGCGCGTTCAATTGATTCACTATCCCCGAATCTCGAAAGGAGAATTGAGGATTGGAATGAACGTATACCAAGAATTCTTGGGGTACCTTGGGGATTCTTGATTGGAGCTGAGCTAACCATAGCCCAAAGTCGTATCTCTTTGGTTAATAAAATCCAAAAGGTTTATCGATCCCAAGGGGTACAGATCCATAATAGGCATATAGAGATTATTATACGCCAAGTAACATCAAAAGTGCGGGTTTCCGAAGATGGAATGTCTAATGTTTTTTCACCTGGGGAATTAATCGGACTATTGCGAGCGGAACGAGCAGGGCGAGCTTTGGATGAATCGATCTATTATCGGGCAATCTTATTGGGAATAACAAGAGCTTCCCTGAATACCCAAAGTTTCATATCTGAAGCAAGTTTTCAAGAAACTGCTCGAGTTTTAGCAAAAGCTGCCCTACGAGGTCGCATTGATTGGTTGAAAGGCTTGAAAGAAAACGTAGTTCTGGGGGGGATTATACCTGTTGGTACCGGATTCCAAAAATTTGTGCATCGTTCCCCACAAGACAAGAACCTTTATTTCGAAATTCAAAAAAAAAATCTATTCGCGTCGGAAATGAGAGATTTTTTGTTTCTCCATACAGAATTAGTTTCTTCTGATTCTGACGTAACAAACAATTTATATGAGACATCAGAACCCCCATTTACCCCCATTTATACGATTTAA

>rps2

ATGACAAGAAGATATTGGAACATCAATTTGAAAGAGATGATAGAAGCGGGAGTTCATTTTGGTCATGGTATTAAGAAATGGAATCCTAAAATGGCCCCTTACATTTCGGCAAAGCGTAAAGGTACTCATATTACAAATCTCGCTAGAACGGCTCGTTTTTTATCAGAAGCTTGTGATTTAGTTTTTGATGCAGCAAGTCAGGGAAAAAGTTTCTTAATTGTTGGTACCAAAAAAAGAGCAGCGGATTTAGTAGCATCAGCTGCAATAAGGGCTCGTTGTCATTATGTTAATAAAAAGTGGTTCAGTGGTATGTTAACGAATTGGTCGATTACGAAAACTAGACTTTCTCAATTTAGAGACTTAAGAGCAGAAGAAAAGATGGGAAAATTCCAGCATCTCCCAAAAAGAGATGTGGCAATCTTGAAGAGAAAATTATCTACCTTGCAAAGATATCTCGGCGGGATCAAATATATGACGAGGTTGCCAGACATTGTGATCGTCCTTGATCAGCAAAAAGAGTATATAGCTCTTCGGGAATGTGCCATTTTGGGGATTCCTACTATTTCTTTAGTCGATACAAATTGTGACCCAGATCTCGCGAATATATCGATTCCAGCCAATGATGACACTATGACTTCAATTCGATTGATTCTTAACAAATTAGTATTTGCAATTTGTGAGGGCCGTTCTCTCTCTATAAGAAATCGTTGA

>atpI

ATGAATATTATACCTTGTTCCATTAAAACACTCAAGGGGTTATACGATATATCGGGTGTAGAAGTAGGCCAACACTTATATTGGCAAATAGGAGGTTTCCAAATTCATGCCCAAGTACTCATCACTTCTTGGGTCGTAATTGCTATCTTGCTAGGTTCAGTTGTCATAGCTGTTCGGAATCCACAAACCATCCCGACCGGCGGTCAGAATTTCTTTGAATATGTCCTTGAGTTTATTCGAGATTTGAGCAAAACTCAGATTGGAGAAGAATACGGTCCCTGGGTTCCCTTTATTGGAACTATGTTCCTTTTTATTTTTGTTTCTAATTGGTCGGGTGCTCTTTTACCTTGGAAAATTATAGAGTTACCTCATGGGGAATTAGCAGCGCCCACGAATGATATAAATACTACTGTTGCTTTAGCTTTACTCACGTCAGCGGCATATTTTTATGCGGGTCTTAGCAAAAAAGGATTGAGTTATTTCGAGAAATATATTAAACCAACTCCAATCCTTTTACCAATTAACATCCTAGAAGATTTCACAAAACCATTATCGCTTAGCTTTCGACTTTTCGGGAATATATTGGCGGATGAATTAGTCGTTGTTGTTCTTGTTTCTTTAGTCCCCTTAGTAGTCCCTATACCGGTCATGTTTCTTGGATTATTTACAAGCGGTATTCAAGCTCTTATTTTTGCAACATTAGCCGCAGCCTATATAGGTGAATCCATGGAGGGTCATCATTGA

>atpH

ATGAATCCACTAATTGCTGCTGCTTCTGTTATTGCTGCTGGATTGGCCGTAGGGCTTGCTTCTATTGGGCCTGGAGTTGGCCAAGGTACTGCTGCAGGACAAGCTGTAGAAGGTATTGCGAGACAGCCAGAAGCAGAAGGTAAAATACGAGGTACTTTATTGCTTAGTCTAGCTTTTATGGAAGCTTTAACAATTTATGGACTAGTTGTGGCACTAGCGCTTTTATTTGCGAACCCTTTTGTTTAA

>atpF

ATGAAAAATGTAACCCATTCTTTCGTTTTTTTAGCTCACTGGCCATCCGCTGGCAGTTTCGGGCTTAATACCGATATTTTAGCAACAAATCTAATAAATCTAACTGTAGTGGTTGGTGTTTTGATTTTTTTTGGAAAGGGAGTGTTAAAAGATTTATTAGATAATCGAAAACAGAGGATCTTGAGTACTATTCGAAATTCGGAAGAACTGCGTAGAGGGACCATGGAGCAGCTCGAAAAAGCTCGGATTCGATTACAGAAAGTCGAACTAGAAGCGGATGAGTATCGAATGAATGGATACTCTGAGATAGAACGAGAAAAAGCAAATTTGATTAATGCCACTTCTATTAGTTTGGAACAATTAGAAAAGTCTAAAAACGAAACCCTTTATTTTGAAAAACAAAGGGCAATGAATCAGGTGCGACAACGGGTTTTCCAACAGGCCGTACAAGGAGCTCTAGGAACTCTGAATAGTTGTTTGAATACCGAGTTACATTTCCGTACGATTCGTGCTAATATTGGCATTCTCGGGTCCATGGAATGGAAGAGATAA

>atpA

ATGGCAACCCTTCGAGTCGACGAAATTCATAAAATTCTTCGCGAACGTATTGAACAATATAATAGGAAAGTAGGGATTGAGAATATTGGTCGCGTAGTTCAAGTGGGGGATGGGATTGCTCGTATTATAGGTCTTGGTGAAATAATGTCAGGTGAATTAGTTGAATTTGCGGAAGGGACTAGGGGTATTGCTCTGAATTTGGAATCCAAAAATGTTGGGATTGTATTAATGGGCGATGGGTTGATGATACAAGAGGGAAGTTTTGTAAAAGCAACAGGAAGAATTGCTCAGATACCCGTGAGCGAGGCTTACTTGGGTCGTGTTATAAATGCTCTGGCTAAACCTATTGATGGGAGAGGCGAAATTATAGCTTCGGAATCTCGCTTAATTGAATCTCCTGCTCCAAGTATAATTTCCAGGCGTTCCGTATATGAACCCCTTCAAACAGGGCTTATTGCTATCGATTCGATGATCCCTATAGGGCGCGGTCAGCGAGAGTTAATTATTGGGGACAGACAGACTGGCAAAACAGCAGTAGCTACAGATACAATTCTCAATCAAAAAGGGCAAGATGTAATATGTGTTTATGTAGCTATCGGTCAAAGAGCATCCTCCGTGGCTCAAGTAGTTACTACTTTCCAAGAGGAGGGGGCCATGGAATACACTATTGTAGTAGCTGAAATGGCGGATTCACCTGCTACATTACAATACCTCGCTCCTTATACGGGAGCAGCCCTGGCTGAGTTTTTTATGTACCGGGAACGGCATACTTTAATAATTTATGATGATCTCTCCAAACAGGCACAAGCTTATCGCCAAATGTCCCTTCTATTAAGAAGACCTCCCGGCCGTGAAGCTTATCCAGGGGATGTTTTTTATTTGCATTCACGCCTTTTAGAAAGAGCCGCTAAATTAAATTCTCTTTTAGGCGAAGGAAGTATGACCGCTTTACCAATAGTTGAGACTCAATCTGGAGACGTTTCTGCCTATATTCCTACTAATGTAATCTCCATTACAGATGGACAAATATTCTTATCCGCGGATCTATTCAATGCCGGAATTCGACCTGCTATTAATGTGGGTATTTCTGTTTCCAGAGTAGGATCTGCGGCTCAAATTAAAGCCATGAAACAAGTAGCTGGCAAATCAAAATTGGAACTAGCTCAATTCGCAGAGTTACAAGCCTTTGCACAATTCGCCTCTGCTCTCGATAAAACAAGTCAGAATCAATTGGCAAGGGGTCGACGATTACGGGAATTGCTTAAACAATCCCAGGCAAACCCTCTCCCAGTAGAAGAGCAGATAGCTACTATTTATACCGGAACGAGAGGATATCTTGATTCGTTAGAAATTGAACAGGTAAATAAATTTCTGGATGAGTTACGTAAACACCTAAAAGATACTAAACCTCAATTCCAAGAAATTATATCTTCTAGCAAGACATTCACCGAGCAAGCGGAAATCCTTTTGAAGGAAGCTATTCAGGAACAGCTCGAGCGGTTTTCCCTTCAGCAACAAACATAA

>rps14

ATGGCAAAAAAAAGTTTGATTCAGAGGGAAAAGAAGCGACAGAAATTAGAACAGAAATATCATTTGATTCGTCAATCTTTAAAAAAAAAGATAAGAAGCAAAGTTTCTCCCTTGAGTTTGAGTGAAAAAACGAAAATGCGAGAAAAATTGCAATCCCTACCACGTAACAGTGCACCTACACGCCTTCATCGACGTTGTTTTTTGACCGGAAGACCTAGAGCTAACTATCGAGACTTTGGGCTATCAGGACACGTACTTCGAGAAATGGTTTATGAATGTTTGTTACCGGGTGCAACAAGATCCAGTTGGTAA

>psaB

ATGGAATTAAGATTTCCCAGGTTTAGCCAAGGCTTAGCTCAGGACCCCACTACTCGTCGTATTTGGTTTGGTATTGCTACCGCACATGATTTCGAAAGTCATGATGATATTACTGAGGAACGTCTTTATCAGAACATTTTTGCTTCTCACTTTGGGCAGTTAGCAATAATCTTTCTATGGACGTCCGGAAATCTGTTTCATGTAGCTTGGCAAGGAAATTTTGAATCATGGATACAGGATCCTTTACACGTAAGACCTATTGCTCATGCGATTTGGGATCCTCATTTTGGTCAACCCGCTGTGGAAGCCTTTACTCGAGGAGGTGCTGCTGGTCCAGTGAATATCGCCTATTCTGGGGTTTATCAGTGGTGGTATACAATAGGATTACGCACCAATGAAGATCTTTATACTGGAGCTCTTTTTCTATTATTTCTTTCTACGCTATCCTTAATAGCGGGTTGGTTACATCTACAACCCAAATGGAAACCAAGCCTTTCGTGGTTCAAAAACGCGGAATCTCGTCTCAATCATCATTTGTCAGGACTTTTCGGGGTAAGTTCTTTGGCTTGGACAGGGCATTTAGTTCATGTTGCTATTCCCGCATCCAGGGGGGAGTACGTTCGATGGAATAATTTCTTAGATGTATTACCCTATCCCCAGGGGTTGGGACCCCTTTTGACGGGTCAGTGGAATCTTTATGCCCAAAACCCCGATTCGAGTAATCATTTATTTGGTACCGCTCAAGGAGCGGGAACTGCCATTCTAACTCTTCTTGGGGGATTCCATCCACAAACACAAAGTTTGTGGCTGACCGATATTGCTCACCATCATTTAGCTATTGCATTCATTTTTCTCATTGCCGGTCACATGTATCGAACTAACTTCGGAATTGGGCACAGTATCAAAGATCTTTTAGAAGCGCATACTCCTCCGGGGGGTCGATTAGGGCGTGGGCATAAGGGCCTTTACGACACAATCAATAATTCGATTCATTTTCAATTAGGTCTTGCTCTAGCTTCTTTAGGGGTTATTACTTCCTTAGTAGCTCAACATATGTACTCTTTACCTGCTTATGCATTCATAGCACAAGACTTTACTACTCAAGCTGCTTTATATACTCATCACCAATATATTGCAGGGTTCATCATGACAGGGGCTTTTGCTCATGGAGCTATTTTTTTCATTAGGGATTACAATCCGGAACAGAATGAGGATAATGTATTGGCAAGAATGTTAGACCATAAAGAAGCTATCATATCTCATTTAAGTTGGGCTAGTCTCTTTCTAGGATTCCATACCTTGGGCCTTTATGTTCATAACGATGTCATGCTTGCTTTTGGTACTCCAGAAAAGCAAATCTTGATTGAACCTATATTTGCCCAATGGATACAATCTGCTCATGGCAAGACGACATATGGGTTCGATATACTCTTATCTTCAACGAATGGCCCCGCTTTCAATGCAGGTCGAAGCCTATGGTTGCCCGGATGGTTGAATGCTGTTAATGAGAATAGTAATTCGCTTTTCTTAACAATAGGACCTGGGGATTTCTTGGTTCATCATGCTATTGCTCTAGGTTTGCATACAACTACATTGATTTTAGTAAAGGGCGCTTTAGATGCACGTGGTTCCAAATTAATGCCGGATAAAAAAGATTTTGGGTATAGTTTTCCTTGTGACGGCCCAGGGCGCGGCGGTACTTGTGATATTTCTGCTTGGGACGCGTTTTATTTGGCGGTTTTCTGGATGTTAAATACCATTGGATGGGTTACTTTTTATTGGCATTGGAAACATATCACATTATGGCAGGGCAACGTTTCACAATTTAATGAATCCTCCACTTATTTGATGGGATGGTTAAGAGATTACCTATGGTTAAACTCTTCACAACTTATCAATGGATATAATCCTTTTGGGATGAACAGTTTATCGGTATGGGCGTGGATGTTCTTATTTGGACATCTTGTTTGGGCTACTGGATTTATGTTCTTAATTTCCTGGCGTGGATATTGGCAGGAATTAATTGAGACTTTAGCATGGGCTCATGAACGCACACCTTTGGCTAATTTAATTCGCTGGAGAGATAAGCCCGTGGCCCTTTCCATTGTGCAAGCAAGATTGGTTGGATTAGCTCACTTTTCCGTGGGTTATATATTCACTTATGCAGCTTTCTTGATTGCCTCAACATCAGGCAAGTTTGGTTAA

>psaA

ATGATTATTCGTTCGCCGGAACCAGAAGTAAAAATTGTTGTGGATAGGGATCCTGTAAAAACATCTTTTGAGGAATGGGCCAGACCCGGCCATTTCTCAAGAACAATAGCTAAGGGCCCTGATACTACCACTTGGATCTGGAACCTACATGCTGATGCTCACGATTTCGATAGTCATACCGGTGATTTGGAGGAGATCTCTCGAAAAGTCTTTAGTGCTCATTTTGGGCAACTCTCCATTATCTTTCTTTGGTTGAGTGGCATGTACTTCCATGGTGCCCGTTTTTCCAATTATGAAGCATGGCTAAGTGATCCTACTCACATTGGACCCAGTGCTCAGGTAGTTTGGCCAATAGTAGGGCAAGAAATATTGAATGGTGATGTAGGCGGGGGTTTCCGAGGAATCCAAATAACCTCTGGGTTTTTTCAGATTTGGCGAGCATCTGGAATAACTAGTGAATTACAACTCTATTGTACCGCAATTGGTGCACTGATTTTTGCATCGTTAATGCTTTTTGCTGGTTGGTTCCATTATCACAAAGCCGCTCCAAAATTGGCCTGGTTCCAAGATGTAGAATCCATGTTGAATCACCACTTAGCGGGGTTATTAGGACTTGGGTCTCTTTCTTGGGCGGGACACCAAATCCATGTATCTTTACCGATTAACCAATTTCTTGACGCTGGGGTTGATCCTAAAGAGATACCACTTCCTCATGAATTTATCTTGAATCGCGACCTTTTGGCTCAACTTTATCCTAGTTTTGCCGAAGGAGCAACCCCCTTTTTCACCTTGAATTGGTCCAAATACGCAGAATTTCTGACTTTTCGCGGAGGACTAGATCCAGTAACCGGTGGTCTATGGCTGACCGATATTGCGCACCATCATTTAGCTATTGCTATTCTTTTCCTGATCGCAGGTCATATGTATAGGACCAACTGGGGTATTGGCCATGGACTTAAAGATATTTTGGAGGCTCACAAGGGGCCATTTACAGGACAAGGCCATAAGGGTCTCTATGAAATCTTAACAACGTCATGGCATGCTCAATTATCTCTTAACCTAGCTATGCTAGGCTCTACAACCATTGTTGTAGCTCATCATATGTATTCTATGCCCCCCTATCCATACCTAGCTACTGACTATGGTACACAACTTTCCTTGTTCACACACCACATGTGGATTGGCGGATTTCTAATAGTCGGTGCTGCTGCACATGCAGCCATTTTTATGGTAAGAGACTATGATCCAACTACTCGATACAACGATCTATTAGATCGCGTCCTTAGACACCGCGATGCAATCATATCCCACCTTAACTGGGTATGTATATTTCTAGGTTTTCACAGTTTTGGCTTGTACATTCATAATGATACCATGAGTGCTTTAGGCCGTCCGCAAGATATGTTTTCCGATACCGCCATACAATTACAACCTATCTTTGCTCAATGGGTACAAAATATCCATGCTAATGCGCCTGGCGTAACAGCTCCCGGTGCAACAACAAGTACTAGCTTAACGTGGGGAGGTGGCGAGTTAGTAGCAGTAGGCGGCAAAGTCGCTTTGTTACCTATTCCATTAGGAACCGCAGATTTTTTAGTCCATCACATTCACGCATTTACCATCCATGTGACTGTATTAATACTTTTGAAAGGTGTTTTATTTGCTCGCAGTTCCCGTTTGATACCCGATAAAGCAAATCTTGGTTTTCGCTTCCCTTGCGATGGGCCTGGGCGAGGGGGAACATGTCAAGTATCTGCCTGGGATCATGTTTTCTTAGGTCTATTCTGGATGTACAATGCAATTTCGGTAGTCATTTTCCATTTCAGTTGGAAAATGCAATCAGATGTTTGGGGTACTATAAGTGATCAAGGGATGGTAACTCATATCACAGGGGGAAACTTTGCACAGAGTTCCATTACGATTAATGGGTGGCTACGAGATTTCTTGTGGGCACAGGCATCGCAAGTAATTCAGTCTTATGGTTCTTCATTATCTGCATATGGTCTTTTTTTCTTAGGTGCTCATTTTGTCTGGGCCTTCAGTTTAATGTTTTTATTCAGCGGCCGTGGTTATTGGCAAGAACTCATTGAATCTATCGTTTGGGCTCATAACAAATTAAAAGTTGCTCCTGCTACTCAGCCTAGAGCCTTGAGCATTATACAAGGACGTGCTGTAGGAGTAACCCATTACCTTCTGGGTGGAATTGCCACAACATGGGCATTCTTCTTAGCGAGAATTATTGCAGTAGGATAG

>ycf3

ATGCCTAGATCCCGCGTAAATGGAAATTTCATTGATAAGACCTCCTCAATTGTAGCCAATATTTTATTGCGAATAATTCCGACAACCTCCGCGGAAAAAAGGGCATTTACTTATTATAGAGATGGTATGTTGGCTCAATCCGAAGGAAATTATGCGGAAGCTTTGCAGAATTATTATGAAGCTACGCGACTAGAAATCGATCCCTATGATCGAAGTTATATACTCTATAACATAGGCCTTATACACACAAGCAATGGAGAGCATACAAAGGCTTTGGAATATTATTTCCGGGCACTAGAACGAAACCCCTTCTTACCGCAAGCTTTTAATAATATGGCCGTGATCTGTCATTACCGAGGAGAACAGGCCATTCTACAGGGTGATTCGGAAATTGCGGAAGCTTGGTTTGATCAAGCTGCTGAGTATTGGAAACAAGCTATAGCGCTTACTCCAGGAAATTATATCGAAGCACAGAACTGGTTGAAGATTACGAAGCGCTTTGAATTTGAATAA

>rps4

ATGTCCCGTTATCGAGGACCTCGTTTAAAAAAAATACGCCGTCTGGGAGCTTTACCAGGACTCACTAGAAAAACACCTAAATCGGGAAGTAATCTGAAAAAGAAATTCCATTCTGGGAAAAAGGAGCAATATCGTATTCGTCTTCAAGAAAAACAGAAATTGCGTTTTCATTATGGTCTGACAGAACGACAATTACTTAGATATGTACATATCGCTGGAAAAGCAAAAAGGTCAACAGGTCAGGTTTTACTACAATTGCTTGAAATGCGTTTAGATAATATCCTTTTTCGATTGGGTATGGCTTCAACCATTCCTGGGGCCCGCCAATTAGTCAACCATAGACATATTTTAGTTAATGGTCGTATAGTGAATATACCAAGTTTTCGTTGCAAACCCCGAGATATTATTACTACGAAAGATAACCAAAGATCAAAAGGTCTGGTTCAAAATTCTATTGCTTCATCCGACCCGGGGAAATTGCCAAAGCATTTGACGATTGACACAGTGGAATATAAAGGACTAGTAAATAAAATCCTAGATAGGAAGTGGGTTGGTCTCAAAATAAATGAGTTGTTAGTTGTAGAATATTACTCTCGTCAGACTTGA

>ndhJ

ATGCAGCAGGGTTGGTTATCTAATTGGTTAGTCAAACATGAAGTGGTTCATAGATCTTTGGGCTTTGATCACCGAGGAATAGAGACTTTACAAATAAAAGCAGGGGATTGGGATTCCATTGCTGTCATTTTATATGTATATGGTTACAATTATTTACGCTCCCAATGTGCTTATGATGTAGCACCCGGTGGATCTTTAGCTAGCGTGTATCATCTTACGAGAATACAGTATGGCATAGATAACCCAGAAGAAGTATGCATAAAAGTCTTTGCCCAAAAGGATAATCCTAGAATCCCGTCTGTCTTCTGGATTTGGAGAAGTGCCGATTTTCAAGAACGCGAATCTTATGATATGGTGGGAATCTCTTATGATAATCATCCACGCCTTAAACGTATCCTAATGCCTGAAAGTTGGATAGGCTGGCCCTTACGTAAGGACTATATAACCCCCAATTTCTATGAAATACAAGATGCTCATTGA

>ndhK

ATGGTCTTAACTGAATATTTAGACAAAAAAAAAAAAGAAGAAAAAGATTCCATTGAGACAGTTATGAATTTGATTGAGTTTCCCTTACTTGACCAAACAAGTTCCAATTCTGTTATTTCAACTACACCAAATGATCTTTCAAATTGGTCAAGACTCTCCAGTTTATGGCCCCTTCTATACGGTACCAGTTGCTGTTTCATTGAATTTGCTTCATTAATAGGCTCACGATTCGACTTTGATCGTTATGGCTTGGTACCAAGATCCAGTCCTAGGCAAGCGGACCTAATTTTAACAGCCGGTACGGTAACAATGAAAATGGCTCCCTCTTTAGTGAGGTTATACGAGCAAATGCCTGAACCAAAATACGTCATTGCTATGGGAGCCTGTACTATTACAGGGGGTATGTTCAGTACGGATTCCTATAGTACTGTTCGGGGAGTTGATAAGTTAATTCCTGTGGATGTCTACTTGCCGGGCTGCCCACCTAAACCGGAGGCAGTTATAGATGCCCTAACAAAACTTCGTAAGAAGATATCGCGAGAAATAGTTGAGGATCGAACTCTATCTCAAAATAAAAATAGATGTTTTACTACCAGTCACAAGCTTTATGTTAGGCGCAGTACTCATACTGGAACTTACGAGCAAGAATTGCTCTATCAATCACCATCTACTTTAGACATATCTTCGGAAACTTTTTTCAAATCCAAAAGTTCAGTACCTTCCTACAAATTAGTGAATTAG

>ndhC

ATGTTTCTGCTTCACGAATATGATATTTTTTGGACATTTTTAATAATAGCAAGCCTTATTCCTATTTTGGCATTTTGGATTTCAGGACTTTTAGCCCCGGTTAGTGAAGGACCAGAGAAGCTTTCTAGTTATGAATCGGGTATAGAACCCATGGGAGGGGCTTGGCTACAATTCCGAATACGCTATTACATGTTTGCGCTAGTTTTTGTTGTTTTTGATGTGGAAACGGTCTTTCTCTACCCTTGGGCAATGAGTTTCGACGTATTGGGTGTATCCGTTTTTATCGAAGCTTTCATTTTCGTGCTTATCCTAGTTGTTGGTTTAGTTTATGCATGGCGAAAAGGAGCTTTGGAATGGTCTTAA

>atpE

ATGAAATTAAATCTTTATGTACTGACTCCTAAGCGAATTATTTGGGATTGTGAAGTGAAAGAAATCATTTTATCCACTAATAGTGGCCAAATTGGCGTATTACCAAACCATGCCCCCATTAACACAGCAGTAGATATGGGTCCTTTGAGAATACGCCTCCTTAACGACCAATGGTTAACGGCGGTTCTGTGGAGCGGTTTTGCGAGAATAGTTAATAATGAGATCATCATTTTAGGAAATGATGCGGAACTGGGTAGTGACATTGATCCGGAAGAAGCTCAACAGGCACTTGAAATAGCCGAAGCTAACTTGAGTAAAGCTGAGGGTACGAAAGAATTGGTTGAAGCGAAGCTAGCTCTCAGACGAGCTAGGATACGAATCGAGGCTGTCAATTGGATTCCCCCATCCAATTGA

>atpB

ATGAGAACCAATCCTACTACGTCTCGTCCCGGGGTTTCCACAATTGAAGAAAAAAGCACAGGTCGTATCGATCAAATTATTGGACCCGTGCTGGATGTCACTTTTCCCCCGGGCAAGTTACCTTATATTTATAACGCTTTGGTAGTCAAGAGTAGAGACACTGCCGATAAGCAAATTAATGTGACTTGTGAGGTACAACAATTATTAGGAAATAATCGAGTTAGAGCTGTAGCTATGAGTGCTACAGACGGGTTGATGAGAGGAATGGAAGTGATTGACACGGGAGCTCCTCTCAGTGTTCCGGTCGGTGGAGCTACTCTCGGACGAATTTTCAACGTTCTTGGGGAGCCTGTTGACAATTTGGGTCCTGTAGATAGTAGTGCAACGTTCCCTATTCATAGATCTGCGCCTGCCTTTATCGAGTTAGATACGAAATTATCTATCTTTGAAACAGGTATTAAGGTGGTCGATCTTTTAGCTCCTTATCGACGTGGAGGAAAAATAGGACTATTTGGGGGGGCTGGAGTAGGTAAAACAGTACTCATTATGGAATTAATCAACAACATAGCTAAAGCTCATGGGGGCGTATCCGTATTTGGTGGAGTAGGGGAACGGACTCGTGAAGGAAATGATCTTTATATGGAAATGAAGGAATCCGGAGTAATTAATGAAAAAAATATTGAGGAATCAAAGGTAGCTCTAGTCTATGGTCAAATGAATGAACCACCGGGAGCTCGTATGAGAGTTGGTTTAACTGCCCTAACTATGGCAGAATATTTCCGAGATGTTAATAAGCAAGATGTGCTTCTATTCATCGATAATATCTTTCGTTTTGTTCAAGCAGGATCGGAGGTCTCTGCTTTATTAGGGAGAATGCCCTCTGCAGTGGGTTATCAACCTACTCTTAGTACAGAAATGGGTTCTTTGCAAGAAAGAATTGCTTCTACTAAAAAGGGATCTATAACCTCGATCCAAGCAGTTTATGTACCCGCGGATGATTTGACCGACCCTGCTCCCGCCACAACATTTGCACATTTGGATGCTACTACCGTACTTTCCAGAGGATTAGCTTCCAAGGGTATTTATCCAGCAGTAGATCCTTTAGATTCAACTTCAACTATGTTACAGCCTCGGATCGTTGGCAACGAACATTATGAAACTGCGCAAAGAGTTAAGGAAACTTTACAACGTTACAAAGAACTTCAGGACATTATCGCAATTCTTGGGTTGGATGAATTATCGGAGGAGGATCGTTTAACTGTAGCAAGAGCACGAAAAATTGAGCGTTTCTTATCACAACCGTTCTTTGTGGCAGAAGTTTTTACCGGTTCTCCAGGAAAGTATGTTGGTCTTGCAGAAACAATTAGGGGATTTCAACTAATCCTTTCCGGAGAATTAGACGGCCTACCCGAACAGGCTTTTTATTTGGTGGGTAACATCGATGAAGCTAGCACGAAAGCTATAACCTTAGAAGAGGAGAACAAATCGAAGAAATGA

>rbcL

ATGTCACCACAAACAGAAACTAAAGCAGGTGTTGGATTTCAAGCTGGTGTTAAAGATTATAAATTGACTTACTACACCCCGGAGTATGAAACCAAGGATACTGATATCTTGGCAGCATTCCGAGTAACTCCTCAGCCCGGGGTTCCGCCCGAAGAAGCGGGGGCTGCAGTAGCTGCCGAATCTTCTACTGGTACATGGACTACTGTTTGGACTGATGGACTTACCAGTCTTGATCGTTACAAAGGACGATGCTATCACATCGAGCCTGTTCCTGGGGAAGAGGATCAATTTATCGCTTATGTAGCTTATCCATTAGACCTATTTGAAGAGGGTTCCGTTACTAACATGTTTACTTCCATTGTGGGTAACGTATTTGGTTTCAAAGCCCTACGTGCTCTACGTCTGGAGGATCTACGAATTCCCCCTACTTATTCAAAAACTTTCCAAGGCCCGCCTCATGGTATCCAAGTTGAAAGAGATAAGTTGAACAAGTATGGTCGTCCTTTATTGGGATGTACTATTAAACCAAAATTGGGATTATCCGCAAAAAATTACGGTAGAGCGTGTTATGAGTGTCTACGTGGTGGACTTGATTTTACCAAAGATGATGAAAACGTAAACTCACAACCATTTATGCGCTGGAGGGACCGTTTTGTCTTTTGTGCCGAAGCTATTTATAAAGCACAGGCCGAAACCGGTGAAATCAAGGGGCATTACTTGAATGCGACTGCAGGTACAGTCGACGAGATGATGAAGAGAGCTGTATTTGCGAGGGAATTAGGGGTTCCTATTGTAATGCATGACTACTTAACAGGGGGATTCACCGCAAATACTACTTTGGCTCATTATTGCCGCGACAACGGCCTACTTCTTCACATTCACCGTGCAATGCATGCAGTTATTGATAGACAGAAAAATCACGGTATGCATTTCCGTGTATTAGCTAAAGCATTGCGTATGTCTGGGGGAGATCATATCCACGCCGGTACAGTAGTAGGTAAGTTAGAAGGGGAACGCGAAATGACTTTAGGTTTTGTTGATTTATTGCGCGATGATTTTATTGAAAAAGATCGTGCTCGCGGTATCTTTTTCACCCAGGACTGGGTATCCATGCCAGGTGTTATACCGGTGGCTTCTGGTGGTATTCATGTTTGGCATATGCCAGCTCTGACCGAAATCTTTGGGGATGATTCCGTATTACAATTTGGTGGAGGAACTTTAGGACATCCTTGGGGAAATGCACCAGGTGCAGCAGCTAATCGAGTGGCTTTAGAAGCTTGTGTACAAGCTCGTAACGAAGGGCGCGATCTTGCTCGTGAAGGTAATGAAATTATCCGAGCAGCTTGCAAATGGAGTCCTGAACTAGCCGCAGCTTGTGAAGTATGGAAGGCGATCAAATTCGAGTTCGCGCCGGTAGATACTATCGATCCGGATATAAAGTCGGATATAAAGAGGATCTAA

>ndhB

ATGGAATCAAACACGCAGTATTTACAGAAAAAAGTCTTCGTTTATTGGGAAAGAATCAATATACTTTTAATGTCGAATCGGAATTCACTAAGACAGAAATAA

>psaI

ATGACGGATTTAAACTTACCTTCTATTTTCGTGCCTTTTGTAGGCTTAGTGTTTCCGGCAATTGCAATGACTTCTTTATTTCTTTATGTGCAGAAAAATAAGATTGTCTAG

>ycf4

ATGAATTGGCGATCAGAACACATATGGGTAGAACTTCTAAAAGGTTCTCGAAAAAGAGGTAATTTTTTCTGGGCCTGTACTCTTTTTCTAGGTTCACTAGGATTCTTATCGGTTGGGGCTTCCAGTTATCTTGGTAAGAATATGATATCTATACTTCCATCTCAACAAATTCTTTTTTTTCCACAGGGGATCGTGATGTCTTTCTACGGAATCGCGGGCCTATTCATTAGCTCCTACTTGTGGTGCACTATTTTGTGGAATGTAGGTAGTGGTTATGACCGCTTCGATAGAAAAGAAGGAATAGTGTGCATTTTTCGTTGGGGATTCCCTGGAATAAAACGTCGCGTCTTCCTTCGATTCCTTATGCGAGATATCCAATCAATTAGAATTCAGGTTAAAGAGGGTCTTTATCCTCGTCGTATCCTTTACATGGAAATCCGGGGCCAGGGGGTCATTCCCTTGACTCGTACTGATGAGAAGTTTTTTACTCCACGAGAAATTGAACAAAAAGCTGCCGAATTGGCCTATTTCTTGCGCGTACCAATGGATGTATTTTGA

>cemA

ATGAAAAAAAAGAAAGCATTGCCTTCTTTACTATATCTTGTATTTATGGTACTTTTGCCTTGGGGGGTCTCTTTCTCTTTTAACAAATGTCTGGAACTTTGGGTTAAGAATTGGTGGAATACCAGGCAATCCGAAACTCTCTTAACTGATATTCAAGAGAAAAGGATTCTAGAAAGATTCATAGAATTAGAAGAACTTTTTCTCTTGGACGAAATGATAAAAGAGAAACCGAAGACACATGTACAAAAACCTCCTATAGGAATACACAAGGAAATAATACAATTGTTCAAAATAGATAATGAGGATCATCTCCATATCATTTTGCATTTCTCGACAAATATAATCTGTTTGGCTATTCTAAGTGGTTCTTTTTTTCTGGGTAAAGAGGAACTTGTCATTTTGAATTCTTGGGTTCAGGAATTCTTCTATAACTTAAATGACTCAATAAAAGCTTTTTTTATTCTTTTAGTTACTGATTTTTTTGTTGGATTTCACTCCACCCGCGGTTGGGAACTAGTAATTCGTTGGGTCTACAACGATCTTGGATGGGCTCCTAATGAGCTAATTTTCACTATTTTTGTTTGTAGTTTTCCAGTAATTCTAGATACATGTTTGAAATTTTGGGTCTTTTTTTGTTTAAACCGTCTATCTCCTTCGCTTGTAGTCATTTATCATTCAATTAGTGAAGCATAA

>petA

ATGGAAAATAGAAATACTTTTTCTTGGGTAAAGGAACAGATGACTCGATCGATTTCTGTATCGATCATAATATACGTAATAACTCGGACATCCATTTCAAATGCATATCCCATTTTTGCGCAGCAGGGTTATGAAAACCCACGAGAAGCTACTGGACGAATTGTATGTGCCAATTGCCATTTAGCTAACAAGCCCGTGGATATTGAAGTTCCCCAAGCTGTGCTTCCCGATACTGTATTTGAAGCAGTTCTTCGAATTCCTTATGATATGCAACTGAAACAAGTTCTTGCTAATGGGAAAAAGGGAGGGTTGAATGTGGGTGCTGTTCTTATTTTGCCCGAGGGATTCGAATTAGCGCCGCCCGACCGTATTTCTCCTGAGTTGAAAGAAAAGATAGGAAATCTCTCTTTTCAGAGTTATCGTCCCGATAAAAAAAATATTCTTGTGATAGGCCCTGTTCCCGGTAAGAAATATAGTGAAATCGTCTTTCCCATTCTTTCCCCTGACCCTGCTACCAATAAAGATGCTCATTTCTTAAAATATCCCATATATGTAGGGGGAAACCGAGGAAGAGGACAGATCTATCCTGATGGTAGCAAGAGTAACAATACGGTCTATAATGCTACGTCAACAGGTATAGTAAGAAAAATACTACGTAAAGAAAAGGGGGGATATGAAATATCCATACTCGATGCATCGGATGGGCGCGAAGTGATCGATATTATACCTCCCGGGCCAGAACTTCTGGTTTCAGAGGGGGAATCCATCAAGCTTGATCAACCGTTAACAAGCAATCCTAATGTGGGAGGGTTTGGTCAGGGGGATGCAGAAATAGTGCTTCAGGATCCATTACGCGTCCAAGGCCTTTTGTTCTTCTTCGCATCCGTTATTTTGGCACAAGTTTTTTTGGTTCTCAAAAAGAAACAGTTTGAAAAGGTTCAATTGTACGAAATGAATTTCTAG

>psbJ

ATGGCCGATACTACTGGAAGAATTCCTCTTTGGCTGATAGGTACTGTAGCTGGTATTCCTGTGATTGGTTTAATAGGTGTTTTCTTTTACGGTTCATATTCTGGATTGGGTTCATCTCTATAG

>psbL

ATGACACAATCAAACCCGAATGAACAAAATGTTGAATTGAATCGTACCAGTCTATACTGGGGTTTATTACTCATTTTTGTACTTGCTGTTTTATTTTCCAATTACTTCTTCAATTGA

>psbF

ATGACCATAGATCGAACCTATCCTATTTTTACAGTGCGATGGCTGGCTATTCACGGACTAGCTGTACCTACTGTTTTTTTCTTGGGATCAATATCAGCAATGCAGTTCATCCAACGATAA

>psbE

ATGTCTGGAAGCACGGGAGAACGTTCTTTTGCTGATATTATTACCAGTATTCGATACTGGGTTATTCATAGCATTACTATACCTTCCCTATTCATTGCGGGTTGGTTATTTGTCAGTACGGGTTTAGCTTATGACGTGTTTGGAAGTCCTCGGCCAAACGAGTATTTCACGGAAAGCCGACAAGGAATTCCGTTAATAACCGACCGTTTTGATTCTTTAGAACAACTCGATGAATTTAGTAGATCCTTTTAG

>petL

ATGCTTACTATAACTAGTTATTTCGGTTTTCTACTGGCTGCTTTAACTATAACCCCAGCTCTATTTATTGGCTTGAACAAGATACGTCTTATTTGA

>petG

ATGATTGAAGTTTTTCTATTTGGAATCGTCTTAGGCCTAATTCCTATTACTTTAGCCGGATTATTCGTGACTGCGTATTTGCAATACAGGCGTGGGGATCAGTTGGATCTTTGA

>psaJ

ATGCGGGATATAAAAACATATCTCTCTGTAGCACCCGTGCTAAGTACTCTATGGTTTGGTGTTTTAGCAGGTTTATTGATAGAAATTAATCGTTTATTCCCAGATGCTTTGTCATTCCCTTTTTTTTAA

>rpl33

ATGGCTAAGGGGAAAGATGTTAGAATCAGAGTTATTTTGGAATGTATCAGTTGTGTTCGAAAAGGTGCCAATGAGGAATCGAGGGGGATTTCTAGATATAGTACTCAAAAGAACCGTCACAATACACCCGGACAATTAGAATTCAAAAAATTTTGTCGTTATTGTCGCAAGCATACGACTCATCACGAAATAAAGAAATAG

>rps18

ATGTATACATCTAAACAACCTTTTCATAAATCTAAGCAACCCTTTCGTAAATCCAAGCAAACTTTTCATAAATCCAAGCAAACCTTTCGTAAATCCAAGCAAACTTTTCGTAAATTCAAACAAACTTTTCGTAAATCCAAACAACCTTTTCGTAGGCGTCCTCGGATTGGCCCGGGAGATCGAATTGATTATAGAAACATGAGTTTAATTAATCGATTTATTAGTGAACAAGGAAAAATATTATCGAGACGAATAAATAGATTAACCTTGAAACAACAACGATTAATTACTCTTGCTATAAAACAGGCTCGTATTTTATCTTTCTTACCATTTCGTAACTATGAGAACGAAAAGCAATTTCAAGCCCAGTCAATTTCAATAATTACAGGTTCTAGACCCAGAAAAAATAGACACATTCCTCAATTAACGCAAAAGTACAATTCCAATCGAAACTTAAGAAACTACAACCAGAATTTAAGAAACAACAATCGGAACTTAAGTTCCGATTGTTGA

>rpl20

ATGACCAGAGTTCCGCGAGGATATATAGCCCGGAGACGACGAACAAAAAAGCGTTCATTTGCCTCAAACTTTAGAGGGGCTCATTTAAGACTTAATCGAATGATTACTCAACAGGTAAAAAGAGCTTTTGTTTCCTCTCATCGAGATAGAGGTAGGCAAAAGAGGGATTTTCGTCGTTTGTGGATCACTCGGATAAACGCAGCAACACGGACATATAAAGTATTCGATAGTTATAGTAAATTAATACACAACCTCTACAAGAAGAAATTGATTCTTAATCGTAAAATGCTTGCACAAGTAGCTGTATCAAATCCAAATAATCTTTACACGATTTCCAATAAAATAAAGATCATCAATTAA

>rps12

ATGCCAACGGTTAAACAACTTATTAGAAACGCAAGACAGCCAATACGAAATGCTAGAAAATCGGCCGCGCTTAAGGGATGTCCTCAGCGTCGAGGAACATGTGCTAGGGTGTATACTATCAACCCCAAAAAACCCAACTCTGCCTTACGTAAAGTTGCCAGAGTACGATTAACCTCTGGATTTGAAATCACTGCTTATATACCTGGTATTGGCCATAATTTACAAGAACATTCTGTAGTATTAGTAAGAGGAGGAAGGGTTAAGGATTTACCCGGTGTGAGATATCGCATTATTCGAGGAACCCTAGATGCTGTCGCAGTAAAGAATCGTCAACAAGGGCGTTCTAAATATGGGGTCAAAAAGCCAAAAAAATAA

>clpP

ATGCCCATTGGTGTTCCAAAAGTACCTTACCGGATTCCCGGAGATGAAGAAGCGACTTGGGTTGACTTATACAATGTTATGTATCGAGAAAGGACACTTTTTTTAGGTCAAGAGATTCGTTGCGAGATCACGAATCATATTACAGGTCTCATGGTATATCTCAGTATAGAAGATGGAATTAGTGATATTTTTTTGTTTATAAACTCCCCAGGCGGGTGGCTAATCTCGGGAATGGCGATTTTTGATACGATGCAAACGGTGACACCAGATATATATACAATATGCCTCGGAATAGCCGCGTCCATGGCGTCCTTCATTCTGCTTGGAGGAGAACCCACCAAGCGTATAGCATTCCCTCACGCGAGGATTATGCTTCACCAACCTGCTAGTGCTTATTATCGGGCAAGGACACCAGAATTTTTACTAGAAGTAGAAGAGTTACACAAAGTTCGCGAAATGATCACAAGGGTTTATGCACTAAGAACAGGCAAGCCTTTTTGGGTTGTATCCGAAGACATGGAAAGGGATGTTTTTATGTCAGCAGACGAAGCCAAAGCTTATGGACTTGTTGATATTGTAGGGGATGAAATGATTGACGAGCACTGCGATACTGATCCAGTGTGGTTTCCAGAAATGTTTAAGGATTGGTAG

>psbB

ATGGGTTTGCCTTGGTATCGTGTTCATACTGTCGTATTGAATGATCCGGGTCGATTGCTTGCGGTGCACATAATGCACACAGCTCTAGTTTCTGGTTGGGCTGGCTCGATGGCTTTATACGAATTAGCGGTTTTTGATCCCTCTGATCCTGTTCTGGATCCAATGTGGAGACAAGGTATGTTCGTCATTCCCTTCATGACTCGTTTAGGAATAACCGATTCGTGGGGTGGTTGGAGTATTTCAGGAGGAACTGTAACGAATCCGGGTATTTGGAGTTATGAAGGTGTGGCAGGTGCGCATATTGTGTTTTCTGGCTTGTGTTTCTTGGCAGCTATCTGGCATTGGGTATATTGGGACCTAGAAATATTCTCTGATGAGCGGACGGGAAAACCCTCTTTGGATTTGCCCAAGATCTTTGGAATTCATTTATTTCTTGCAGGGGTGGCCTGCTTTGGTTTTGGCGCATTCCATGTAACGGGTTTGTATGGTCCTGGGATATGGGTGTCCGATCCTTATGGACTAACTGGAAAAGTACAAGCTGTAAATCCGGCGTGGGGTGCAGAAGGTTTTGATCCTTTTGTTCCGGGGGGAATAGCTTCTCATCATATTGCTGCGGGTACATTGGGCATATTAGCGGGTCTATTCCATCTTAGTGTCCGTCCGCCTCAACGTCTATACAAAGGATTACGTATGGGCAATATTGAAACTGTACTTTCCAGTAGTATCGCTGCTGTTTTTTTTGCAGCTTTCGTAGTTGCTGGAACTATGTGGTATGGGTCAGCAACGACCCCAATCGAATTATTTGGGCCTACTCGTTATCAGTGGGATCAGGGATACTTTCAGCAAGAAATATATCGAAGAGTTAGCAATGGGTTAGCCGAAAATCTTAGTTTATCAGAAGCTTGGTCTAAAATTCCCGAAAAATTAGCCTTTTATGATTATATTGGTAATAATCCGGCAAAAGGGGGATTATTCAGAGCAGGCTCAATGGACAATGGGGATGGAATAGCTGTTGGATGGTTAGGACATCCCGTCTTTAGAGATAAAGAAGGACGCGAGCTTTTTGTACGCCGTATGCCTACTTTTTTTGAAACATTTCCGGTTGTTTTGGTAGATGAAGAGGGAATTGTGAGAGCGGACGTTCCTTTTAGAAGAGCAGAATCCAAATATAGTGTTGAACAAGTAGGCGTAACGGTGGAGTTCTATGGTGGCGAACTTAATGGAGTAAGTTATTCTGATCCTGCTACCGTAAAAAAATATGCGAGGCGTTCTCAATTAGGGGAAATTTTTGAATTAGATCGGGCTACTTTGAAATCAGATGGTGTTTTTCGCAGCAGTCCAAGGGGTTGGTTCACTTTTGGTCATGCTACCTTTGCTTTGCTCTTCTTTTTCGGGCACATTTGGCATGGCGCTAGAACCTTGTTCCGAGATGTTTTTGCTGGTATTGATCCAGATTTGGATGCTCAAGTGGAATTTGGAACATTCCAAAAAGTTGGAGATCCAACTACAAGGAAACAGGCAGTCTGA

>psbT

ATGGAAGCATTGGTTTATACGTTCCTTTTAGTTTCGACTTTAGGGATAATTTTTTTCGCTATCTTCTTCCGAGAACCGCCAAAGGTTCCGACTAAAAGAACAAAATAA

>psbN

ATGGAAACAGCAACTTTAGTCGCCATCTCCATATCTGGTTTACTTGTAAGCTTTACTGGGTATGCCTTATATACCGCGTTTGGGCAACCCTCTCAACAATTAAGAGATCCATTCGAAGAACACGGAGACTAA

>psbH

ATGGCTACACAAACCGTTGAAGATAGTTCTAAACCTAGGCCAAAACGAACTGGTGCAGGTAGTTTATTGAAACCCTTGAATTCGGAATATGGGAAAGTCGCTCCGGGTTGGGGGACTACTCCTTTTATGGGGGTCGCTATGGCTTTATTCGCGATATTCCTATCTATCATTTTAGAAATTTATAATTCTTCTGTTTTACTGGACGGAATTTTAACGAATTAG

>petB

ATGAGTAAAGTATATGATTGGTTTGAGGAACGTCTTGAGATTCAAGCAATTGCAGATGATATAACTAGTAAATATGTTCCTCCTCATGTCAACATATTTTATTGTTTAGGGGGAATTACACTTACTTGTTTTCTAGTACAAGTCGCTACCGGTTTTGCTATGACTTTTTACTACCGCCCAACCGTTACAGAAGCTTTTTCCTCGGTTCAATACATAATGACCGAGGCCAACTTTGGTTGGTTAATTCGATCAGTTCATCGATGGTCAGCAAGTATGATGGTTCTAATGATGATCCTGCATGTATTTCGTGTGTATCTCACAGGTGGATTTAAAAAACCCCGCGAATTAACTTGGGTCACAGGTGTGGTTTTGGCTGTATTGACTGCATCGTTTGGTGTAACTGGTTATTCTTTGCCTTGGGATCAAATTGGTTATTGGGCAGTCAAAATTGTGACAGGCGTACCTGACGCGATTCCGGTAATAGGATCGCCTTTAGTGGAGTTATTACGTGGAAGTGCTAGTGTGGGCCAATCCACTTTGACTCGTTTTTATAGTTTACATACCTTTGTACTGCCTCTGCTTACTGCCGTATTTATGTTAATGCACTTTCCAATGATACGTAAGCAAGGTATTTCGGGTCCTTTATAG

>petD

ATGGGAGTAACAAAGAAACCTGACTTAAATGATCCTGTATTAAGAGCAAAATTAGCTAAAGGGATGGGACATAATTATTACGGGGAACCCGCGTGGCCCAACGATCTTTTATATATTTTTCCAGTAGTAATTCTAGGTACTATTGCATGTAATGTAGGTTTAGCGGTTCTCGAGCCGTCAATGATCGGTGAACCGGCGGATCCGTTTGCAACTCCTCTGGAAATATTACCCGAGTGGTACTTCTTTCCCGTGTTTCAAATACTTCGTACGGTACCCAATAAGTTATTGGGCGTTCTCTTAATGGTTTCTGTGCCAACAGGCTTATTGACAGTCCCTTTTCTAGAGAATGTCAATAAATTCCAAAATCCATTTCGTCGCCCAGTAGCTACGACCGTTTTTTTAATTGGTACTGTAGTAGCTCTTTGGTTAGGTATTGGAGCAACATTACCCATTGATAAATCCTTAACTTTAGGTCTTTTTTAG

>rpoA

ATGGTTCGAGAGGAGGTAGCAGGATCCACTCAAACACTACAGTGGAAGTGTGTTGAATCAAGAGTAGATAGTAAGCGTCTTTATTATGGCCGTTTCATTCTGTCCCCGCTTAGAAAAGGTCAAGCGGACACCGTTGGTATTGCCTTGCGAAGAGCTTTACTTGGAGAAATAGAAGGAACATGTATCACACGTGCAAAATTTGGGAGCTTGCCACACGAATATTCTACAATAGCAGGTATTGAAGAATCCGTACAAGAAATTTTACTAAATTTGAAAGAAATTGTATTGAGAAGTAATCTCTATGGAGTTAGAGACGCATCAATTTGGGTCAAAGGTCCTAGATACATAACTGCTCAAGATATCATCTTACCACCTTCCGTAGAAATCGTTGATACGGCACAACCTATAGCTAACTTGACAGAGCCCATTGATTTCTGTATTGAGTTACAGATCAAGAGAGATCGTGGATATCAGACGGAACTCAGAAATAACTATCAAGATGGAAGTTATCCTATAGATGCTGTATCCATGCCTGTTCGAAATGTGAATTATAGTATTTTTTCTTGTGGGAATGGAAATGAAAAACACGAGATACTTTTTCTAGAAATATGGACTAATGGAAGTTTAACCCCTAAGGAAGCGCTTTATGAGGCTTCTCGTAATTTGATTGATTTATTTCTTCCTTTTCTACACGCGGAGGAAGAGGGCGCTAGTTTCGAAGAAAATAAAAACAGGTTTACTCCACCCCTTTTTACTTTTCAAAAAAAATTAACTAATCTAAAGAAAAACAAAAAAGGAATTCCATTGAATTGTATTTTTATTGATCAATTAGAATTGCCTTCTAGAACGTATAATTGTCTCAAAAGGGCCAATATACATACACTATTGGACCTTTTGAGTAAGACTGAAGAAGATCTTATGAGAATTGACAGTTTTCGTATGGAAGATGGAAAACAGATATGGGACACTCTAGAGAAGCATCTGCCAATTGATTTACTTAAGAATAAGCTCTCGTTTTAA

>rps11

ATGGCAAAAGCTATACCAAAAATAGGTTCACGTAAGAAAGTGCGTATTGGTTTACGTAGGAATGCACGTTTTAGTTTACGGAAGAGTGCACGTAGAATAACAAAAGGGGTTATTCATGTTCAAGCTAGTTTCAACAATACCATTATAACTGTTACAGACCCACAAGGTCGGGTGGTTTTCTGGTCCTCCGCGGGTACTTGTGGATTCAAAAGCTCACGAAAAGCATCACCCTATGCTGGCCAAAGAACAGCAGTAGATGCTATTCGTACAGTAGGTTTGCAACGAGCAGAAGTTATGGTAAAGGGCGCTGGTAGTGGAAGAGATGCCGCATTACGAGCCATTGCTAAAAGTGGTGTACGATTAAGTTGTATACGCGATGTAACACCTATGCCGCATAATGGATGTCGACCGCCTAAAAAAAGACGTCTGTAA

>rpl36

ATGAAAATAAGAGCTTCCGTTCGTAAAATTTGTACAAAATGTCGACTGATTCGTAGGCGTGGACGAATTAGAGTCATTTGTTCCAATCCGAAGCATAAACAAAGACAGGGGTAA

>infA

ATGACAGAAAAAAAAAATAGGAGAGAAAAAAAAAACCCGAGAGAAGCAAAAGTCACTTTCGAAGGTTTAGTTACGGAAGCTCTACCCAACGGAATGTTCCGCGTTCGCCTAGAGAATGACACCATCATCCTGGGCTATATTTCAGGAAAGATCCGGTCTAGTTCTATACGAATACTGATGGGGGATAGGGTAAAAATTGAAGTAAGTCGTTATGATTCAAGCAAGGGACGTATAATTTATAGACTTCCACATAAGGATTCGAAGCGTACCGAAGACTCAAAGGATATCGAAGATTTGAAGGATAGCGAAGATTTGAAAGAGACCAAAGATTCAAAGGATTAG

>rps8

ATGGGCAAGGACACTATTGCTGATTTACTAACCTCTATAAGAAATGCGGACATGAATAAAAAAGGAACAGTTCGAGTAGTATCGACAAATATTACCGAAAACATTGTTAAAATACTTCTACGAGAGGGTTTTATTGAAAGTGTTCGGAAACATCAGGAAAGTAACAGATATTTCTTGGTTTCAACTTTGCGACATCAAAAGAGAAAGACTAGAAAAGGAATATATAGAACAAGAACCTTTTTAAAGCGTATCAGCCGACCCGGCTTACGAATTTATACCAACTATCAAGGAATTCCTAAAGTTTTGGGTGGAATGGGAATTGCTATTCTTTCTACTTCTCGAGGGATAATGACAGATCGAGAGGCTCGACTAAACAAAATTGGGGGAGAAGTCTTATGTTATATATGGTAA

>rpl14

ATGATTCAACCCCAGACCCTTTTAAATGTAGCAGATAATAGTGGAGCGCGAAAATTGATGTGTATTCGAGTCATAGGAGCTGCTGGTAATCAGCGATATGCTCGTATTGGTGATGTTATTGTTGCTGTAATCAAAGACGCAGTGCCCCAAATGCCTCTAGAAAGATCCGAAGTAATTCGAGCTGTAATTGTACGTACATGTAAAGAGTTCAAATGCGAAGACGGTATAATAATCCGCTATGATGACAACGCGGCGGTTATAATTGATCAAAAAGGAAACCCAAAAGGAACTCGAGTTTTTGGCGCGATCGCCGAGGAATTGAGAGAATTGAATTTTACTAAAATAGTTTCATTAGCTCCTGAAGTATTATAA

>rpl16

ATGCTTAGTCCCAAAAGAACCAGATTTCGCAAACAACATAGAGGAAGAATGAAGGGAAAATCCTGCCGAGGCAATCGTATTTGTTTTGGTAGATATGCTCTTCAAGCACTTGAACCCGCTTGGATCACGGCGAGACAGATAGAAGCGGGACGAAGAGGAATAACACGATATGCACGTCGTGGTGGAAAAATATGGGTACGTATATTTCCCGACAAACCGGTTACACTAAGACCCACGGAAACACGTATGGGCTCAGGAAAGGGGTCCCCCGAATATTGGGTAGCCATTGTTAAACCAGGTCGAATACTTTATGAAATGGGCGGAGTATCCGAAACTGTAGCTAGAGCAGCTATCTCCATAGCTGCCAGTAAAATGCCCATACGAAGTCAATTTATTCGATTAGAGATATAG

>rps3

ATGGGACAAAAAATAAATCCACTCGGTTTCAGACTTGGTACAACCCAAAAACACCATTCCTTTTGGTTCGCACAACCAAAAAATTATTCTGAAGGTATACAGGAAGATAAAAAAATAAGGAATTGTATCAAGAACTATATACAAAAGAATAGGAAAAAGGGCTCGAATAGAAAAATAGAATCAGACTCAAGTTCCGAAGTAATTACACATAATAGAAAAACGGACTCAGGCTCAAGTTCTGAAGTAATTACACATATAGAAATTCAAAAAGAAATCGATACGATCCACGTCATAATCCATATAGGATTCCCTAATTTATTAAAGAAAAAAGGAGCAATCGAAGAATTAGAGAAAGATCTACAAAAGGAAGTTAACTCTGTAAACCAGAGACTTAATATTGCTATCGAAAAAGTGAAAGAACCTTATAGACAACCTAACATTCTTGCAGAATATATAGCATTCCAATTAAAAAATAGAGTTTCATTCCGAAAAGCAATGAAAAAAGCCATTGAATTAACTAAAAAAGCAGATATAAGGGGAGTAAAAGTAAAAATTGCGGGCCGTCTCGGAGGAAAAGAAATTGCACGTGCGGAATGCATCAAAAAGGGTAGACTTCCCCTCCAAACAATTCGCGCTAAAATTGATTATTGCTGCTATCCAATTCGAACTATCTATGGAGTATTAGGTGTAAAAATTTGGATATTCGTAGACGAAGAATAA

>rpl22

ATGACAAGTTTCAAACTAGTAAAGTATATCCCTAGGATAAAGAAAAAGAAAAGTGGGCTAAGGAAACTCGCAAGGAAAGTTCCAACCGATCGTCTACTTAAGTTCGAGCGAGTTTTCAAAGCACAAAAACGTATCCATATGTCTGTTTTCAAAGCACAAAGAGTTCTTGATGAGATTCGCTGGCGTTACTACGAGGAAACTGTTATGATACTGAACCTCATGCCTTATCGAGCATCTTATCCCATTTTAAAGTTGGTTTATTCGGCAGCAGCAAATGCTACTCATTATAGGGATTTCGACAAAGCTAATTTATTCATCACTAAAGCCGAAGTCAGTAGGAGTACTATTATGAAAAAATTAAGACCCCGGGCTCGAGGACGTAGTTTTCCCATAAAAAAAACCATGTGTCATATAACAATTGTACTAAATATAGTAAAGAAATCTAAATAA

>rps19

ATGAAGGAGGAGAAAGAAACAATAGTAACGTGGTCCCGGGCATCTAGCATTCTACCCACAATGGTTGGCCATACAATCGCGATTCATAATGGAAAGGAACATATACCTATTTACATAACAAATCCTATGGTAGGTCGCAAATTGGGGGAATTCGTGCCTACTCGGCATTTCACGAGTTATGAAAATGCAAGAAAGGATACTAAATCTCGTCGTTAA

>rpl2

ATACTTAATAATACGGCGAAACATTTATACAAAACACCTATCCCGAGCACACGCAAGGGAACCGTAGACAGGCAAGTGAAATCCAATCCACGAAATAATTTGATCCATGGACGGCACCGTTGTGGTAAAGGTCGTAATTCCAGAGGAATCATTACCGCAAGGCATAGAGGGGGAGGTCATAAGCGCCTATACCGTAAAATCGATTTTCGACGGAATCAAAAAGACATATCTGGTAGAATCGTAACCATAGAATACGACCCTAATCGAAATGCATACATTTGTCTCATACACTATGGGGATGGTGAGAAGAGATATATTTTACATCCCAGAGGGGCTATAATTGGAGATACTATTGTTTCTGGTACAAAAGTTCCTATATCAATGGGAAATGCCCTACCTTTGACCGATATGCCCTTAGGCACGGCCATGCATAACATAGAAATCACACGTGGAAGGGGTGGGCAATTAGCTAGAGCAGCAGGTGCTGTAGCGAAACTGATTGCAAAAGAGGGTAAATCGGCCACTTTAAGATTACCATCTGGGGAGGTCCGTTTAGTATCCCAAAACTGCTTAGCAACAGTCGGACAAGTGGGTAATGTTGGGGTGAACCAAAAAAGTTTGGGTAGAGCCGGATCTAAGTGTTGGCTAGGTAAACGCCCCGTAGTAAGGGGGGTAGTTATGAACCCTGTGGACCACCCCCATGGGGGCGGTGAAGGGAAAGCCCCTATTGGTAGAAAAAAACCCACAACCCCTTGGGGTTATCCTGCGCTTGGAAGAAGAACTAGGAAAAGGAAAAAATATAGCGATAGTTTTATTCTTCGTCGCCGTAAGTAA

>rpl23

ATGGATGGAATCAAACACGCAGTATTTACAGAAAAAAGTCTTCGTTTATTGGGAAAGAATCAATATACTTTTAATGTCGAATCGGGATTCACTAAGACAGAAATAAAGCATTGGGTCGAACTCTTCTTTGGTGTTAAGGTAGTAGCTGTGAATAGCCATCGACTACCCGGAAAGGGTAGAAGAATAGGACCTATTCTGGGACATACAATGCATTACAGACGTATGATCATTACCCTTCAACCGGGTTATTCTATTCCACTTCTAGATAGAGAAAAAAACTAA

>ndhB

ATGATCTGGCATGTACAGAATGAAAACTTCATTCTCGATTCTACGAGAATTTTTATGAAAGCGTTTCATTTGCTTCTCTTCCATGGAAGTTTCATTTTCCCAGAATGTATCCTAATTTTTGGCCTAATTCTTCTTCTGATGATCGATTCAACCTCTGATCAAAAAGATAGACCTTGGTTCTATTTCATCTCTTCAACAAGTTTAGTAATAAGCATAACGGCCCTATTGTTCCGATGGAGAGAAGAACCTATAATTAGCTTTTCGGGAAATTTCCAAACGAACAATTTCAACGAAATCTTTCAATTTCTCATTTTATTATGTTCAACTTTATGTATTCCTCTATCCGTAGAGTACATTGAATGTACAGAAATGGCTATAACAGAGTTTCTGTTATTCGTATTAACAGCTACTCTAGGGGGAATGTTTTTATGTGGTGCTAACGATTTAATAACTATCTTTGTAGCTCCAGAATGTTTCAGTTTATGTTCCTACCTATTGTCTGGATATACCAAGAGAGATCTACGCTCTAATGAGGCTACTATGAAATATTTACTCATGGGTGGGGCAAGCTCTTCTATTCTGGTTCATGGTTTCTCTTGGCTATATGGTTCATCTGGGGGGGAGATCGAGCTTCAAGAAATAGTGAATGGTCTTATCAATACACAAATGTATAACTCCCCAGGAATTTCAATTGCGCTTATATCCATCACTGTAGGACTTGGGTTCAAGCTTTCCCCAGCCCCTTTTCATCAATGGACTCCTGACGTCTACGAAGACTCCCCCACTCCAGTCGTTGCTTTTCTTTCTGTTACTTCGAAAGTAGCTGCTTCAGCTTCAGCCACGCGAATTCTCGATATTCCTTTTTATTTCTCATCAAACGAATGGCATCTTCTTCTGGAAATCCTAGCTATTCTTAGCATGATATTGGGGAATCTCCTTGCTATTACTCAAACAAGCATGAAACGTATGCTTGCATATTCGTCCATAGGGCAAATCGGATATGTAATTATTGGAATAATTGTTGGAGACTCAAATGATGGATATGCAAGCATGATAACTTATATGCTGTTCTATATCTCCATGAATCTAGGAACTTTTGCTTGCATTGTATTATTTGGTCTACGTACCGGAACTGATAACATTCGAGATTATGCAGGATTATACACGAAAGATCCTTTTTTGGCTCTCTCTTTAGCCCTATGTCTCTTATCCCTAGGAGGCCTTCCTCCACTAGCAGGTTTCTTCGGAAAACTCTATCTATTCTGGTGTGGATGGCAAGCAGGCCTATATTTCTTGGTTTCAATAGGACTCCTTACGAGCGTTCTTTCTATCTACTATTATCTAAAAATAATCAAGTTATTAATGACTGGACGAAACCAAGAAATAACCCCTTATGTGCGAAATTATAGAAGATCCCCTTTAAGATCAAACAATTCCATCGAATTGAGTATGACTGTATGTGTGATAGCATCTACTATACCAGGAATATCAATGAACCCCATTCTTGCAATTGCTCAGGATACCCTCTTTTAG

>rps7

ATGTCACGTCGAGGTACTGCAGAAAAAAGAACTGCAAAATCCGATCCAATTTTTCGTAATCGATTAGTTAACATGGTGGTTAACCGTATTATGAAAGACGGAAAAAAATCATTGGCTTATCAAATTCTCTATCGAGCCGTGAAAAAGATTCAACAAAAGACAGAAACAAATCCACTATTGGTTTTACGTCAAGCAATACGTAGAGTAACTCCCAATATAGGAGTAAAAACAAGACGTAATAAAAAAGGATCGACGCGGAAAGTTCCGATTGAAATAGGATCTAAACAAGGAAGAGCACTTGCCATTCGTTGGTTATTAGAAGCATCCCAAAAGCGTCCGGGTCGAAATATGGCTTTCAAATTAAGTTCCGAATTAGTAGATGCTGCCAAAGGGAGTGGGGGTGCCATACGCAAAAAGGAAGCGACTCATAGAATGGCAGAGGCAAATAGAGCTCTTGCACATTTTCGTTAA

>rps15

ATGAAAAAGAAAGGAGGTAGAAAAATTTTGGGATTTATGGTTAAAGAAGAAAAAGAAGAAAACAGGGGTTCTGTTGAATTTCAAGTATTCAGTTTCACCAATAAGATACGGAGACTTGCTTCACATTTGGAATTACACAAAAAAGATTTTTCATCGGAAAGAGGTCTACGAAGACTTTTGGGAAAACGTCGACGTTTGCTGGCTTATTTGGCAAAGAAAAATAGAGTACGTTATAAGAAATTAATCAGTCAGTTGAATATCCGGGAGCAGTAA

>ndhH

ATGAGTCTACCGCTTACAAGAAAAGATCTCATGATAGTCAATATGGGCCCTCAACACCCATCAATGCATGGTGTTCTTCGACTGATCGTTACTCTCGATGGTGAAGATGTTATTGATTGTGAACCCATATTAGGCTATTTACACAGAGGAATGGAAAAAATCGCAGAAAACCGAACTATTAGGAGAGGGGGGGTAGGAAAGTCATTTAGGTAG

>ndhF

ATGGAACATACATATCAATATGCATGGGTAATCCCTCTTCTCCCACTTCCAGTTATTATGTCAATGGGGTTTGGCCTTATTCTTATTCCGACCACAACAAAAAATCTTCGTCGTATATGGGCTTTTCCTAGTGTTTTACTCTTAAGTGTAGCTATGGTATTCTCAGTTCAACTGTCTATTCAACAAATAAATGGAAGTTCTATCTATCAATATCTATGGTCTTGGACCGTCAATAATGATTTTTCCTTAGAATTTGGATACTTGATTGACCCCCTTACTTCTATTATGTTAATACTAATTACTACTGTAGGAATCCTGGTTCTTATTTATAGTGACGGTTATATGTCTCACGATGAAGGATATTTGAGATTTTTTGTTTATATAAGTTTTTTCAATACTTCCATGTTGGGATTGGTTACTAGCTCCAATTTGATACAAATTTATTTTTTTTGGGAGCTTGTGGGAATGTGTTCCTATTTATTGATAGGCTTTTGGTTTACACGACCAATCGCAGCGAGTGCTTGTCAAAAAGCTTTTGTAACTAATCGTGTAGGGGATTTTGGTCTGTTATTGGGAATTTTAGGTTTTTTTTGGGTAACAGGTAGTTTAGAGTTTCGGGATTTGTTCCAAATAGCTAATAATTGGATTCCTAATAATGGGATTAATTCTTTACTTACTACTTTGTGTGCTTTTTTATTATTCCTTGGTGCAGTTGCGAAATCTGCACAATTCCCTCTTCACGTATGGTTACCCGATGCTATGGAAGGACCCACTCCCATTTCGGCTCTTATACACGCAGCAACTATGGTTGCTGCGGGGATTTTTCTTCTAGCTCGACTTCTTCCTCTTTTCATATCTTTACCATTTATAATGAGTTTCATTTCTTTAGTAGGTACAATAACACTCTTCTTAGGGGCTACTTTAGCTCTTGCTCAGAGAGATATTAAAAAAAGCTTAGCCTATTCTACAATGTCTCAATTGGGTTATATGATGTTAGCTCTAGGTATAGGTTCTTATCAAGCTGCTTTATTCCATTTGATCACTCATGCTTATTCAAAAGCTTTATTATTCTTGGGATCCGGATCCGTTATTCATTCAATGGAACCTATTGTTGGATATTCACCAGATAAAAGTCAGAATATGGTTCTTATGGGCGGTTTAAGAAAATACATTCCAATTACAAGAACTACTTTTTTATGTGGTACACTTTCTCTTTGTGGTATTCCACCTCTTGCTTGCTTCTGGTCCAAAGATGAAATCCTTAGTAATAGTTGGTTGTATTCGCCCTTTTTTGGAATAATAGCCTCCTTTACTGCAGGATTAACTGCGTTTTATATGTTTCGGATATATTTACTTACGTTTGATGGGTATTTGCGTGTTCATTTTCAAAATTACAGTAGCACTAAAAAGGGTTCGTTGTATTCAATATCCTTATGGGGAAAAAGGATAACCAAAGGAGTGAATAGGAATTTCGTTTTACCAACAACGAAGAGTAGAGTTTCTTTTTTTTCACAAAATTTATCCAAAATTCATGGTAATACAAGAAATAGGATAGGATCCTTTAGTACCTCCTTTGGAGCTAAAAAGACTTTAGCCTATCCGCATGAAACGGGAAATACTATGTTATTTCCTCTTCTTATATTGCTGCTTTTTACTTTGTTCATTGGATTCATAGGAATCTCTTTTGATAATGGAGCAATAGATAATGGAATAGCAGAGTTAACCATATTATCAAAGTGGTTAACTCCCTCAATAAACTTTATCCAGGAAAGTTCTAATTCTTTTATAAATTCATATGAATTTATCACTAATGCAATTTCTTCTGTAAGTATAGCTATCTTCGGTTTATTCATAGCATATAGCTTCTATGGATCCGCTTATTGTTTTTTTCATAATTTGTATTTAATAAATTTCTTTGTAAAAGGGAGTCCGAAAAAGTCCTTTTTCGATCAAGTAAAAAAAAAGATATACAGTTGGTCATATAATCGTGGTTATATAGATATTTTCTATACTAGGGTCTTTACCTTCGGTATAAGAGGATTAACCGAACTAACTGAGTTTTTCGATAAGGGTGTTATTGATGGAATTACCAATGGAGTGGGTCTTGCTAGTTTTTGTATAGGAGAAGAAATTAAATATGTAGGGGGAGGGCGAATCTCGTCTTATTTATTCTTTTTTTTATGTTATCTATCCGTGTTTTTATTATTTTTTCTTTCTTAA

>rpl32

ATGGCAGTTCCAAAAAAACGTACTTCAATGTCAAAAAAGCGTATTCGTAAAAATCTTTGGAAGAAAAAGACTTATTTTTCCATAGTACAATCTTATTCTTTAGCAAAATCAAGATCATTTTCTAGCGGCAACGAGCATCCAAAACCAAAGGGTTTTTCTGGGCAACAAACAAATAATCAGGTTTTGGAATAA

>ccsA

ATGCTATTTGCAACTTTAGAACATATACTAACTCACATCTCTTTCTCAACAATTTCAATTGTGATTACGATTCATTTGATAACCTTATTAGTTCGTGAACTTGGGGGATTACGTGATTCGTTAGAAAAAGGAATGATAGCTACTTTTTTTTCTATAACAGGATTCCTAGTTTCTCGTTGGGCTTCTTCGGGACATTTTCCATTAAGTAATTTATATGAGTCATTGATCTTCCTTTCATGGGCTCTGTATATTCTTCATATGATTCCTAAGATACAGAACTCTAAAAATGATTTAAGCAGAATAACAACGCCAAGTACTATTTTAACGCAAGGCTTTGCCACGTCGAGTCTTTTAACTGAAATGCATCAATCCACAATACTAGTACCTGCTTTACAATCTCAGTGGTTAATGATGCATGTCAGTATGATGTTACTAAGCTATGCAACTCTTTTGTGCGGATCCTTATTATCCGCCGCTCTTCTAATCATTAGATTTCGAAATAATTTCCAGTTCTTTTCTAAAAAGAAAAAAAATGTTTTATTTAACACATTTTTCTTTAGTGAGATTGAAAATTTCTATGCAAAAAGAAGTGCTTTAAAAAGCACCTCTTTTCCTTCATTTACAAATTATTACAAATATCAATTAACTGAGCGTTTGGATTCTTGGAGTTATCGTGTCATTAGCCTAGGATTTACCCTTTTAACCATAGGTATTCTTTGTGGAGCAGTATGGGCTAATGAGGCGTGGGGATCCTATTGGAATTGGGATCCTAAGGAAACTTGGGCATTTATTACTTGGACTATATTCGCAATTTATTTACATAGTAGAACAAATCCAAATTGGAAGGGTACGAATTCCGCACTTGTAGCTTCGATAGGATTTCTTATAATTTGGATCTGCTATTTTGGTATCAATCTATTAGGAATAGGTTTACATAGTTATGGTTCGTTTACATTAACACCTAAATGA

>ndhD

ATGAGTTATTTTCCTTGGTTAACAATACTTGTTGTTTTGCCGATATTTGCAGGTTCATTAATTTTCTTTTTACCTCATAGGGGAAACAAAATCGTTAGGTGGTATACTATATCTATTTGTTTATTAGAATTCCTTCTAATGACTTATGCATTCTGTTACCATTTCCAACTGGAGGATCCCTTAATCCAATTAAAGGAGGATTCTAAATGGATAGATGTCTTCGATTTCCACTGGAGATTGGGAATCGATGGACTTTCATTAGGATCTATTTTATTGACAGGATTTATCACTACTTTAGCTACTTTAGCAGCTTGGCCAGTTACCCGGAATTCGCGATTATTCTATTTCCTGATGCTAGCAATGTATAGTGGTCAAATAGGATTATTTTCTTCGCGAGACCTTTTACTTTTTTTTATCATGTGGGAGTTAGAATTAATTCCTGTTTACTTACTTTTATCCATGTGGGGGGGAAAGAGGCGTCTATATTCAGCTACAAAGTTTATTTTGTATACTGCGGGCGGTTCCTTTTTTTTCTTAATCGGAGTTCTGGGTATGGGCTTATATGGTTCCAACGAACCAGGGTTAGATTTGGAAAGATTAATTAATCAATCATACCCTGCAACTTTGGAAATACTTTTCTATTTTGGCTTCCTTATTGCTTATGCTGTCAAATTGCCGATTATACCCCTACATACGTGGTTACCAGATACCCATGGGGAAGCGCATTATAGTACATGTATGCTTTTAGCGGGAATCCTATTAAAGATGGGAGCATATGGATTGATTCGGATCAACATGGAATTGTTACCGCATGCTCATTATCTATTTTCGCCCTGGTTGGTAATAATAGGAGCGATCCAAATAATCTATGCAGCTTCAACTTCTCTTGGTCAACGAAATTTCAAAAAAAGAATAGCCTATTCCTCTGTATCTCACATGGGTTTCATAATTATAGGAATTGGTTCCATAACCAACATTGGACTCAATGGAGCTATTTTACAAATATTATCCCATGGATTTATTGGTGCTACACTTTTTTTCTTGGCAGGAACGGCTTGTGATAGAATGCGTCTTGTTTATCTCGAAGAACTGGGGGGGATATCTATCCCAATGCCGAAAATTTTTACCATGTTTAGCAGCTTTTCAATGGCTTCTCTTGCCTTACCAGGAATGAGCGGTTTTGTCGCAGAATTAGTAGTATTTTTTGGGCTAATTACTAGTCCAAAATTTCTGTTAATGCCAAAAACCCTAATTACTTTTGTAATGGCAATTGGAATGATATTAACTCCTATTTATTTATTATCTATGTTACGCCAGATGTTCTACGGATACAAGCTATTTTATGTTCCAAATGCAAATTTTGTGGATTCTGGACCACGAGAACTCTTTCTTTTAATCTGTATCTTTTTACCAGTAATAGGAATTGGTATTTATCCAGATTTTGTTCTCTCCCTATCCGTTGACAGGGTAGAGGCTCTCTTATCCAATTTTTATCCTAAATAG

>psaC

ATGTCACATTCCGTAAAAATTTATGATACATGTATAGGATGCACTCAATGTGTACGAGCTTGTCCAACAGATGTATTAGAAATGATACCCTGGGATGGATGTAAAGCCAAGCAAATTGCTTCCGCGCCGAGAACCGAAGATTGTGTGGGTTGTAAGAGATGCGAATCTGCCTGCCCAACAGATTTTTTAAGTGTCCGCGTTTATTTAGGACCTGAAACAACCCGTAGCATGGCTCTATCTTATTGA

>ndhE

ATGATGTTTGAGCATGTACTTTTTTTGAGTGTCTTTTTATTTTCGATTGGTATCTATGGATTGATCACAAGCCGAAACATGGTTAGAGCTCTAATATGTCTTGAACTTATACTGAATTCAATTAATCTAAATCTCGTAACATTTTCTGATCTATTTGATAGTCGCCAATTAAAAGGAGACATTTTCGCAATTTTTGTTATAGCCCTTGCGGCTGCTGAAGCCGCTATTGGACTATCCATTCTTTCTTCCATCCATCGTAACAGGAAATCCACTCGTATCAATCAATCTAATTTTTTGAATAATTAG

>ndhG

ATGGATTTACCTGGACCAATACATGAGATTCTTGTGCTATTTGGGGGATTTGTTCTTCTACTAGGGGGTCTAGGAGTAGTATTACTTACCAACCCAATTTATTCTGCTTTTTCGCTGGGATTAGTTCTTGTTTGTATATCCTTATTCTATTTTTTATTGAATTCCTACTTTGTAGCTGTCGCACAACTTCTTATTTATGTGGGAGCCATAAATGTCTTGATCATATTCGCTGTAATGTTCATAAATGGCTCAGAATGGTCTAAAGATAAGAATTATTGGACTATTGGAGACGGGTTCACTTCACTCGTTTGTATAACTATTGTTTTTTCACTAATGACTACTATCCCAGATACGTCATGGTATGGAATTCTTTGGACTACAAGATCAAACCAAATAGTAGAACAGGGTCTCATAAATAATGTTCAACAAATTGGGATTCATTTAGCAACCGATTTTTATCTTCCGTTTGAACTCATTTCCATAATTCTTCTAGTTTCTTTAATAGGTGCAATTACTATGGCTCGGCAATAA

>ndhI

ATGTTCCCTATGGTAACTGGGTTCATGAGTTATGGTCAACAAACAATACGCGCTACAAGGTACATTGGTCAAAGTTTCATAACTACCTTATCCCACACAAATCGTTTACCTATAACGATTCACTACCCTTACGAAAAATCAATTACACCGGAGCGTTTCCGAGGGCGAATCCACTTTGAATTTGATAAATGTATTGCTTGTGAAGTCTGTGTTCGCGTATGTCCAATAGATCTACCCCTTGTGGATTGGAGATTTGAAAAGGATATTAAAAGGAAACAATTGCTTAATTATAGTATTGATTTCGGAGTTTGTATATTTTGTGGTAATTGTGTTGAGTACTGTCCAACAAGCTGTTTATCAATGACGGAAGAATATGAACTTTCTACTTATGATCGTCATGAATTGAATTACAATCAAATTGCTTTGAGTCGGTTACCAATCTCCATAATGGGAGATTACACAATTCAAACAATTAGGAATTCGACTGAAAGTAAAATAAACGAAGATAAATCTTCGAATTCAAGAACGATTACTGATTACTAG

>ndhA

ATGATAATAGATAGGGTAGAGGTAGAAACTATCAATTCTTTTTCGAAATCGGAATTATTAAAAGAAGTCTATGGACTAATATGGATTCTACCCATTTTGATCCTCCTTTTGGGAATCACAATAGAGGTACTCGTAATTGTGTGGTTAGAAAGAGAAATATCTGCATCGATACAACAACGTATTGGTCCTGAATATGCTGGCCCCCTGGGCCTGCTTCAAGCTATAGCGGATGGGACTAAGCTACTTTTTAAAGAGGATATCCTGCCATCCCGAGGAGATATTCCTTTATTTAGCATTGGACCCTCTATAGCAGTCATATCCATTTTATTAAGTTTTTTAGTTATCCCTTTGGGATATCGTTTTGTTTTAGCCGATCTTAGTATTGGTGTTTTTTTATGGATTGCCATTTCAAGTATTGCTCCTATTGGTCTTCTTATGGCAGGATATAGCTCAAATAATAAATATTCTTTTTCAGGCGGTCTACGAGCTGCTGCTCAATCCATTAGTTATGAAATACCATTAACTTTTTGTGTGCTAGCAATATCTCTACTATCTAACAGTTCAAGTACAGTTGATATAGTTGAAGCACAGTCAAAATATGGTTTTTTTGGATGGAATCTTTGGCGTCAGCCTATAGGTTTTCTGGTTTTTCTAATTTCTTCTTTGGCAGAATGTGAAAGATTGCCCTTTGATTTACCAGAAGCGGAGGAAGAATTAGTAGCAGGTTATCAAACAGAATATTCCGGTATCAAATATGGTTTATTTTATCTTGTTTCTTACCTAAATTTATTAGTTTCCTCTTTATTTGTAACAGTTCTTTACTTAGGCGGGTGGAATTTCTCTATTCCCTTTATATCCTTTTTTGAATTTTTTCAAATGAATAAAGCAGTTGGAATTTTGGAAATGACAATGGGTATCTTTATTACATTAACTAAAGCTTATTTATTTCTCTTCATTTCTATCACAATAAGATGGACTTTACCCAGGATGAGAATGGACCAGTTATTAAATCTTGGGTGGAAATTTCTTTTACCTATTTCCCTGGGCAATCTCTTATTAACAACTTCTTCCCAACTTGTTTCACTATAA

>ndhH

ATGAGTCTACCGCTTACAAGAAAAGATCTCATGATAGTCAATATGGGCCCTCAACACCCATCAATGCATGGTGTTCTTCGACTGATCGTTACTCTCGATGGTGAAGATGTTATTGATTGTGAACCCATATTAGGCTATTTACACAGAGGAATGGAAAAAATCGCAGAAAACCGAACTATTATACAATACTTACCCTATGTAACACGGTGGGATTATTTAGCTACTATGTTTACAGAAGCAATAACGGTAAATGCACCTGAATTCTTGGAGAATATTCAAATACCCCAAAGAGCCAGTTATATTAGGGTAATTATGTTAGAGTTGAGCCGTATAGCCTCTCACTTGTTATGGCTTGGGCCCTTTATGGCGGATCTCGGCGCACAGACTCCTTTTTTCTATATTTTTAGAGAGAGAGAATTAATATATGATCTATTTGAAGCTGCTACAGGTATGCGAATGATGCACAATTACTTTCGCATCGGAGGAGTAGCCACCGATCTACCTTATGGATGGATCGATAAATGTTTAGATTTCTGTGATTATTTTTTACGCGGAGTTGTTGAATATCAACAACTTATTACAGAGAATCCTATTTTTTTAGAACGAGTTGAAGGAGTAGGTTTTATTATCGGAGAAGAAGCAGTAAATTGGGGCTTATCGGGACCAATGTTACGGGCTTCTGGAATACAATGGGATCTTCGTAAAGTAGATCCTTATGAGTCTTACAACCAATTCGATTGGAAAGTCCAATGGCAAAAAGAAGGGGATTCGTTAGCTCGCTATTTAGTACGAGTTGGTGAAATGAGGGAATCCATCAAAATTATTCAACAGGCAGTAGAGAAAATTCCTGGAGGACCTTACGAGAATTTAGAAGTCCGACGCTTTAAGAAAGCAAAGAATTCCGAATGGAATGATTTTGAATATAGATTTCTTGGTAAAAAACCTTCGCCAAATTTTGAATTGTCAAAGCAAGAGCTTTATGTAAGAGTGGAAGCTCCAAAAGGTGAATTAGGGATTTATCTAGTAGGAGATGATAGTCTTTTCCCCTGGAGATGGAAAATTAGGCCACCCGGTTTTATTAATTTGCAAATTCTTCCTCAGCTAGTTAAAAAAATGAAATTGGCTGATATCATGACGATATTAGGTAGTATAGATATCATTATGGGGGAAGTTGATCGTTGA

>rps15

ATGAAAAAGAAAGGAGGTAGAAAAATTTTGGGATTTATGGTTAAAGAAGAAAAAGAAGAAAACAGGGGTTCTGTTGAATTTCAAGTATTCAGTTTCACCAATAAGATACGGAGACTTGCTTCACATTTGGAATTACACAAAAAAGATTTTTCATCGGAAAGAGGTCTACGAAGACTTTTGGGAAAACGTCGACGTTTGCTGGCTTATTTGGCAAAGAAAAATAGAGTACGTTATAAGAAATTAATCAGTCAGTTGAATATCCGGGAGCAGTAA

>rps7

ATGTCACGTCGAGGTACTGCAGAAAAAAGAACTGCAAAATCCGATCCAATTTTTCGTAATCGATTAGTTAACATGGTGGTTAACCGTATTATGAAAGACGGAAAAAAATCATTGGCTTATCAAATTCTCTATCGAGCCGTGAAAAAGATTCAACAAAAGACAGAAACAAATCCACTATTGGTTTTACGTCAAGCAATACGTAGAGTAACTCCCAATATAGGAGTAAAAACAAGACGTAATAAAAAAGGATCGACGCGGAAAGTTCCGATTGAAATAGGATCTAAACAAGGAAGAGCACTTGCCATTCGTTGGTTATTAGAAGCATCCCAAAAGCGTCCGGGTCGAAATATGGCTTTCAAATTAAGTTCCGAATTAGTAGATGCTGCCAAAGGGAGTGGGGGTGCCATACGCAAAAAGGAAGCGACTCATAGAATGGCAGAGGCAAATAGAGCTCTTGCACATTTTCGTTAA

>ndhB

ATGATCTGGCATGTACAGAATGAAAACTTCATTCTCGATTCTACGAGAATTTTTATGAAAGCGTTTCATTTGCTTCTCTTCCATGGAAGTTTCATTTTCCCAGAATGTATCCTAATTTTTGGCCTAATTCTTCTTCTGATGATCGATTCAACCTCTGATCAAAAAGATAGACCTTGGTTCTATTTCATCTCTTCAACAAGTTTAGTAATAAGCATAACGGCCCTATTGTTCCGATGGAGAGAAGAACCTATAATTAGCTTTTCGGGAAATTTCCAAACGAACAATTTCAACGAAATCTTTCAATTTCTCATTTTATTATGTTCAACTTTATGTATTCCTCTATCCGTAGAGTACATTGAATGTACAGAAATGGCTATAACAGAGTTTCTGTTATTCGTATTAACAGCTACTCTAGGGGGAATGTTTTTATGTGGTGCTAACGATTTAATAACTATCTTTGTAGCTCCAGAATGTTTCAGTTTATGTTCCTACCTATTGTCTGGATATACCAAGAGAGATCTACGCTCTAATGAGGCTACTATGAAATATTTACTCATGGGTGGGGCAAGCTCTTCTATTCTGGTTCATGGTTTCTCTTGGCTATATGGTTCATCTGGGGGGGAGATCGAGCTTCAAGAAATAGTGAATGGTCTTATCAATACACAAATGTATAACTCCCCAGGAATTTCAATTGCGCTTATATCCATCACTGTAGGACTTGGGTTCAAGCTTTCCCCAGCCCCTTTTCATCAATGGACTCCTGACGTCTACGAAGACTCCCCCACTCCAGTCGTTGCTTTTCTTTCTGTTACTTCGAAAGTAGCTGCTTCAGCTTCAGCCACGCGAATTCTCGATATTCCTTTTTATTTCTCATCAAACGAATGGCATCTTCTTCTGGAAATCCTAGCTATTCTTAGCATGATATTGGGGAATCTCCTTGCTATTACTCAAACAAGCATGAAACGTATGCTTGCATATTCGTCCATAGGGCAAATCGGATATGTAATTATTGGAATAATTGTTGGAGACTCAAATGATGGATATGCAAGCATGATAACTTATATGCTGTTCTATATCTCCATGAATCTAGGAACTTTTGCTTGCATTGTATTATTTGGTCTACGTACCGGAACTGATAACATTCGAGATTATGCAGGATTATACACGAAAGATCCTTTTTTGGCTCTCTCTTTAGCCCTATGTCTCTTATCCCTAGGAGGCCTTCCTCCACTAGCAGGTTTCTTCGGAAAACTCTATCTATTCTGGTGTGGATGGCAAGCAGGCCTATATTTCTTGGTTTCAATAGGACTCCTTACGAGCGTTCTTTCTATCTACTATTATCTAAAAATAATCAAGTTATTAATGACTGGACGAAACCAAGAAATAACCCCTTATGTGCGAAATTATAGAAGATCCCCTTTAAGATCAAACAATTCCATCGAATTGAGTATGACTGTATGTGTGATAGCATCTACTATACCAGGAATATCAATGAACCCCATTCTTGCAATTGCTCAGGATACCCTCTTTTAG

>rpl23

ATGGATGGAATCAAACACGCAGTATTTACAGAAAAAAGTCTTCGTTTATTGGGAAAGAATCAATATACTTTTAATGTCGAATCGGGATTCACTAAGACAGAAATAAAGCATTGGGTCGAACTCTTCTTTGGTGTTAAGGTAGTAGCTGTGAATAGCCATCGACTACCCGGAAAGGGTAGAAGAATAGGACCTATTCTGGGACATACAATGCATTACAGACGTATGATCATTACCCTTCAACCGGGTTATTCTATTCCACTTCTAGATAGAGAAAAAAACTAA

>rpl2

ATACTTAATAATACGGCGAAACATTTATACAAAACACCTATCCCGAGCACACGCAAGGGAACCGTAGACAGGCAAGTGAAATCCAATCCACGAAATAATTTGATCCATGGACGGCACCGTTGTGGTAAAGGTCGTAATTCCAGAGGAATCATTACCGCAAGGCATAGAGGGGGAGGTCATAAGCGCCTATACCGTAAAATCGATTTTCGACGGAATCAAAAAGACATATCTGGTAGAATCGTAACCATAGAATACGACCCTAATCGAAATGCATACATTTGTCTCATACACTATGGGGATGGTGAGAAGAGATATATTTTACATCCCAGAGGGGCTATAATTGGAGATACTATTGTTTCTGGTACAAAAGTTCCTATATCAATGGGAAATGCCCTACCTTTGACCGATATGCCCTTAGGCACGGCCATGCATAACATAGAAATCACACGTGGAAGGGGTGGGCAATTAGCTAGAGCAGCAGGTGCTGTAGCGAAACTGATTGCAAAAGAGGGTAAATCGGCCACTTTAAGATTACCATCTGGGGAGGTCCGTTTAGTATCCCAAAACTGCTTAGCAACAGTCGGACAAGTGGGTAATGTTGGGGTGAACCAAAAAAGTTTGGGTAGAGCCGGATCTAAGTGTTGGCTAGGTAAACGCCCCGTAGTAAGGGGGGTAGTTATGAACCCTGTGGACCACCCCCATGGGGGCGGTGAAGGGAAAGCCCCTATTGGTAGAAAAAAACCCACAACCCCTTGGGGTTATCCTGCGCTTGGAAGAAGAACTAGGAAAAGGAAAAAATATAGCGATAGTTTTATTCTTCGTCGCCGTAAGTAA

>rps19

ATGAAGGAGGAGAAAGAAACAATAGTAACGTGGTCCCGGGCATCTAGCATTCTACCCACAATGGTTGGCCATACAATCGCGATTCATAATGGAAAGGAACATATACCTATTTACATAACAAATCCTATGGTAGGTCGCAAATTGGGGGAATTCGTGCCTACTCGGCATTTCACGAGTTATGAAAATGCAAGAAAGGATACTAAATCTCGTCGTTAA
