# Supplementary material for: Complete chloroplast genomes of Achnatherum inebrians and comparative analyses with related species from Poaceae
Source: FEBS Open Bio. 2021 May 10;11(6):1704–18. doi: 10.1002/2211-5463.13170 (PMC8167873; doi:10.1002/2211-5463.13170)
Supplement: Supplementary file 5 — Table S4. Frequency of classified repeat types (considering sequence complementary). [file FEB4-11-1704-s002.docx]

| **Repeats** | **3** | **4** | **5** | **6** | **7** | **8** | **9** | **10** | **11** | **12** | **total** |
| --- | --- | --- | --- | --- | --- | --- | --- | --- | --- | --- | --- |
| A/T | - | - | - | - | - | - | - | 12 | 5 | 3 | 20 |
| C/G | - | - | - | - | - | - | - | 2 |  |  | 2 |
| AG/CT | - | - | 2 |  |  |  |  |  |  |  | 2 |
| AT/AT | - | - | 4 | 1 |  |  |  |  |  |  | 5 |
| AAG/CTT | - | 1 |  |  |  |  |  |  |  |  | 1 |
| AAT/ATT | - | 1 |  |  |  |  |  |  |  |  | 1 |
| AAAC/GTTT | 1 |  |  |  |  |  |  |  |  |  | 1 |
| AAAG/CTTT | 2 |  |  |  |  |  |  |  |  |  | 2 |
| AAAT/ATTT | 3 |  |  |  |  |  |  |  |  |  | 3 |
| AACG/CGTT | 2 |  |  |  |  |  |  |  |  |  | 2 |
| AAGG/CCTT | 1 |  |  |  |  |  |  |  |  |  | 1 |
| AATG/ATTC | 1 |  |  |  |  |  |  |  |  |  | 1 |
| ACAT/ATGT | 1 |  |  |  |  |  |  |  |  |  | 1 |
| ACCT/AGGT |  | 1 |  |  |  |  |  |  |  |  | 1 |

**Table S4** Frequency of classified repeat types (considering sequence complementary)
