# Supplementary material for: Complete chloroplast genomes of Achnatherum inebrians and comparative analyses with related species from Poaceae
Source: FEBS Open Bio. 2021 May 10;11(6):1704–18. doi: 10.1002/2211-5463.13170 (PMC8167873; doi:10.1002/2211-5463.13170)
Supplement: Supplementary file 6 — Table S5. The relevant data of phylogenetic tree generated by BI. [file FEB4-11-1704-s005.docx]

**Table S5** The relevant data of phylogenetic tree generated by Bayesian Inference

IQ-TREE 1.6.12 built Aug 15 2019

Input file name: concatenation.fas

Type of analysis: ModelFinder + tree reconstruction

Random seed number: 546103

REFERENCES

----------

To cite ModelFinder please use:

Subha Kalyaanamoorthy, Bui Quang Minh, Thomas KF Wong, Arndt von Haeseler,

and Lars S Jermiin (2017) ModelFinder: Fast model selection for

accurate phylogenetic estimates. Nature Methods, 14:587–589.

https://doi.org/10.1038/nmeth.4285

SEQUENCE ALIGNMENT

------------------

Input data: 39 sequences with 56770 nucleotide sites

Number of constant sites: 32587 (= 57.4018% of all sites)

Number of invariant (constant or ambiguous constant) sites: 32587 (= 57.4018% of all sites)

Number of parsimony informative sites: 15057

Number of distinct site patterns: 5530

ModelFinder

-----------

Best-fit model according to AICc: GTR+F+I+G4

List of models sorted by AICc scores:

Model LogL AIC w-AIC AICc w-AICc BIC w-BIC

GTR+F+I+G4 -256385.8584 512941.7168 + 0.6188 512941.9747 + 0.6181 513702.1917 - 0.0182

GTR+F+G4 -256387.3428 512942.6856 + 0.3812 512942.9375 + 0.3819 513694.2137 + 0.9818

HKY+F+I+G4 -256586.8041 513335.6082 - 0.0000 513335.8425 - 0.0000 514060.2960 - 0.0000

HKY+F+G4 -256589.0869 513338.1738 - 0.0000 513338.4024 - 0.0000 514053.9149 - 0.0000

GTR+F+I -257325.4655 514818.9310 - 0.0000 514819.1829 - 0.0000 515570.4591 - 0.0000

HKY+F+I -257518.2460 515196.4920 - 0.0000 515196.7206 - 0.0000 515912.2331 - 0.0000

SYM+I+G4 -259411.3659 518986.7318 - 0.0000 518986.9719 - 0.0000 519720.3664 - 0.0000

SYM+G4 -259422.9118 519007.8236 - 0.0000 519008.0579 - 0.0000 519732.5114 - 0.0000

K2P+I+G4 -259495.6194 519147.2388 - 0.0000 519147.4562 - 0.0000 519845.0863 - 0.0000

K2P+G4 -259505.2856 519164.5712 - 0.0000 519164.7831 - 0.0000 519853.4720 - 0.0000

GTR+F -259909.8482 519985.6964 - 0.0000 519985.9424 - 0.0000 520728.2778 - 0.0000

HKY+F -260113.9665 520385.9330 - 0.0000 520386.1560 - 0.0000 521092.7273 - 0.0000

SYM+I -260354.2554 520870.5108 - 0.0000 520870.7451 - 0.0000 521595.1986 - 0.0000

K2P+I -260435.3927 521024.7854 - 0.0000 521024.9973 - 0.0000 521713.6862 - 0.0000

F81+F+G4 -261622.7401 523403.4802 - 0.0000 523403.7032 - 0.0000 524110.2745 - 0.0000

F81+F+I+G4 -261623.2393 523406.4786 - 0.0000 523406.7072 - 0.0000 524122.2197 - 0.0000

F81+F+I -262536.1031 525230.2062 - 0.0000 525230.4292 - 0.0000 525937.0005 - 0.0000

SYM -263189.6055 526539.2110 - 0.0000 526539.4396 - 0.0000 527254.9521 - 0.0000

K2P -263233.3125 526618.6250 - 0.0000 526618.8314 - 0.0000 527298.5790 - 0.0000

JC+I+G4 -264098.7475 528351.4950 - 0.0000 528351.7069 - 0.0000 529040.3958 - 0.0000

JC+G4 -264100.1691 528352.3382 - 0.0000 528352.5446 - 0.0000 529032.2922 - 0.0000

JC+I -265025.2381 530202.4762 - 0.0000 530202.6826 - 0.0000 530882.4302 - 0.0000

F81+F -265028.1651 530212.3302 - 0.0000 530212.5476 - 0.0000 530910.1777 - 0.0000

JC -267633.9146 535417.8292 - 0.0000 535418.0303 - 0.0000 536088.8364 - 0.0000

AIC, w-AIC : Akaike information criterion scores and weights.

AICc, w-AICc : Corrected AIC scores and weights.

BIC, w-BIC : Bayesian information criterion scores and weights.

Plus signs denote the 95% confidence sets.

Minus signs denote significant exclusion.

SUBSTITUTION PROCESS

--------------------

Model of substitution: GTR+F+I+G4

Rate parameter R:

A-C: 0.9710

A-G: 2.8380

A-T: 0.5992

C-G: 0.9063

C-T: 2.9504

G-T: 1.0000

State frequencies: (empirical counts from alignment)

pi(A) = 0.3019

pi(C) = 0.1923

pi(G) = 0.1998

pi(T) = 0.306

Rate matrix Q:

A -0.8481 0.169 0.5133 0.1659

C 0.2653 -1.246 0.1639 0.817

G 0.7753 0.1577 -1.21 0.2769

T 0.1637 0.5134 0.1808 -0.858

Model of rate heterogeneity: Invar+Gamma with 4 categories

Proportion of invariable sites: 0.06349

Gamma shape alpha: 0.7598

Category Relative_rate Proportion

0 0 0.06349

1 0.09287 0.2341

2 0.4169 0.2341

3 1.009 0.2341

4 2.752 0.2341

Relative rates are computed as MEAN of the portion of the Gamma distribution falling in the category.

TREE USED FOR ModelFinder

-------------------------

Log-likelihood of the tree: -256380.9093 (s.e. 1104.2409)

Unconstrained log-likelihood (without tree): -226122.6736

Number of free parameters (#branches + #model parameters): 85

Akaike information criterion (AIC) score: 512931.8186

Corrected Akaike information criterion (AICc) score: 512932.0765

Bayesian information criterion (BIC) score: 513692.2934

Total tree length (sum of branch lengths): 0.9146

Sum of internal branch lengths: 0.5555 (60.7389% of tree length)

NOTE: Tree is UNROOTED although outgroup taxon 'Achnatherum_splendens_MK704435' is drawn at root

+--Achnatherum_splendens_MK704435

|

| +--Aeluropus_lagopoides_NC_042858

| +--|

| | | +--Chloris_truncata_NC_032033

| | | +--|

| | | | +--Chloris_virgata_NC_032034

| | | +--|

| | | | +--Cynodon_dactylon_NC_034680

| | | +--|

| | | | +--Enteropogon_ramosus_NC_042834

| | +--|

| | +--Eleusine_indica_NC_030486

| +--|

| | +--Dactyloctenium_radulans_NC_042838

| +--|

| | | +--Arundo_donax_NC_037077

| | | +--|

| | | | +--Arundo_plinii_NC_034652

| | +--|

| | +--Molinia_caerulea_MF035989

| +--|

| | | +--Alloteropsis_angusta_NC_027951

| | | +--|

| | | | | +--Setaria_viridis_NC_028075

| | | | +--|

| | | | | +--Urochloa_brizantha_NC_030067

| | | | +--|

| | | | +--Urochloa_decumbens_NC_030066

| | +--|

| | | +--Andropogon_ascinodis_NC_040129

| | | +--|

| | | | +--Sorghum_bicolor_NC_008602

| | +--|

| | | +--Tripsacum_dactyloides_NC_037087

| | +--|

| | +--Zea_nicaraguensis_KU291447

| +--|

| | | +----------------Cyperus_rotundus_NC_050170

| | +----------------------------------------|

| | +--Eleocharis_dulcis_NC_047447

| +--|

| | | +--Chikusichloa_aquatica_NC_027184

| | | +--|

| | | | +--Chikusichloa_mutica_NC_041081

| | | +--|

| | | | +--Zizania_latifolia_NC_029401

| | +--|

| | | +--Leersia_japonica_NC_034766

| | +--|

| | +--Leersia_perrieri_KY347906

| +--|

| | | +--Agrostis_gigantea_NC_037162

| | | +--|

| | | | +--Agrostis_stolonifera_NC_008591

| | | +--|

| | | | | +--Alopecurus_japonicus_MN422307

| | | | +--|

| | | | | +--Cynosurus_cristatus_KY432806

| | | | | +--|

| | | | | | | +--Lolium_arundinaceum_NC_011713

| | | | | | +--|

| | | | | | +--Lolium_perenne_NC_009950

| | | | +--|

| | | | +--Holcus_lanatus_NC_036689

| | | +--|

| | | | +--Littledalea_alaica_NC_037519

| | +--|

| | +--Brachypodium_distachyon_NC_011032

+--|

| | +--Stipa_hymenoides_NC_027464

| +--|

| +--ZMC_ZMC

|

| +--Oryzopsis_asperifolia_NC_027479

+--|

+--Stipa_purpurea_NC_029390

Tree in newick format:

(Achnatherum_splendens_MK704435:0.0021074372,((((((((Aeluropus_lagopoides_NC_042858:0.0055018143,((((Chloris_truncata_NC_032033:0.0020660778,Chloris_virgata_NC_032034:0.0031023530):0.0026793553,Cynodon_dactylon_NC_034680:0.0051705173):0.0001372245,Enteropogon_ramosus_NC_042834:0.0026458984):0.0011745648,Eleusine_indica_NC_030486:0.0053523217):0.0019114461):0.0002191139,Dactyloctenium_radulans_NC_042838:0.0068818249):0.0091503098,((Arundo_donax_NC_037077:0.0000983597,Arundo_plinii_NC_034652:0.0003450808):0.0056499007,Molinia_caerulea_MF035989:0.0046018149):0.0009749630):0.0006267916,((Alloteropsis_angusta_NC_027951:0.0112544429,(Setaria_viridis_NC_028075:0.0039574765,(Urochloa_brizantha_NC_030067:0.0002836049,Urochloa_decumbens_NC_030066:0.0003924407):0.0069800600):0.0018627128):0.0020891484,((Andropogon_ascinodis_NC_040129:0.0034663803,Sorghum_bicolor_NC_008602:0.0019785851):0.0004603403,(Tripsacum_dactyloides_NC_037087:0.0005443093,Zea_nicaraguensis_KU291447:0.0017391375):0.0012945618):0.0062611197):0.0048239471):0.0062471805,(Cyperus_rotundus_NC_050170:0.1806009674,Eleocharis_dulcis_NC_047447:0.0364099259):0.4317519073):0.0025750334,(((Chikusichloa_aquatica_NC_027184:0.0001826724,Chikusichloa_mutica_NC_041081:0.0003139412):0.0028237166,Zizania_latifolia_NC_029401:0.0061368101):0.0018588946,(Leersia_japonica_NC_034766:0.0004074242,Leersia_perrieri_KY347906:0.0006421462):0.0121383162):0.0085304539):0.0084529145,((((Agrostis_gigantea_NC_037162:0.0016484982,Agrostis_stolonifera_NC_008591:0.0022086287):0.0075382906,(Alopecurus_japonicus_MN422307:0.0068551477,((Cynosurus_cristatus_KY432806:0.0101069183,(Lolium_arundinaceum_NC_011713:0.0113872629,Lolium_perenne_NC_009950:0.0022252166):0.0062812711):0.0004171957,Holcus_lanatus_NC_036689:0.0060191806):0.0008501659):0.0036655172):0.0038770904,Littledalea_alaica_NC_037519:0.0060381118):0.0024580021,Brachypodium_distachyon_NC_011032:0.0182355251):0.0027413228):0.0026210578,(Stipa_hymenoides_NC_027464:0.0016529069,ZMC_ZMC:0.0023875695):0.0036551677):0.0006756020,(Oryzopsis_asperifolia_NC_027479:0.0019344795,Stipa_purpurea_NC_029390:0.0021920528):0.0000531133);

TIME STAMP

----------

Date and time: Mon Nov 30 01:23:46 2020

Total CPU time used: 501.984 seconds (0h:8m:21s)

Total wall-clock time used: 353.523 seconds (0h:5m:53s)
