# Supplementary material for: Complete chloroplast genomes of Achnatherum inebrians and comparative analyses with related species from Poaceae
Source: FEBS Open Bio. 2021 May 10;11(6):1704–18. doi: 10.1002/2211-5463.13170 (PMC8167873; doi:10.1002/2211-5463.13170)
Supplement: Supplementary file 7 — Table S6. The relevant data of phylogenetic tree generated by maximum likelihood. [file FEB4-11-1704-s007.docx]

**Table S6** The relevant data of phylogenetic tree generated by Maximum likelihood

Input file name: F:/Bioinformation_software/PhyloSuite/myWorkPlace/GenBank_File/ZMC\IQtree_results\2020_11_30-01_13_26\concatenation.fas

Type of analysis: tree reconstruction + ultrafast bootstrap (5000 replicates)

Random seed number: 917383

REFERENCES

----------

To cite IQ-TREE please use:

Lam-Tung Nguyen, Heiko A. Schmidt, Arndt von Haeseler, and Bui Quang Minh

(2015) IQ-TREE: A fast and effective stochastic algorithm for estimating

maximum likelihood phylogenies. Mol Biol Evol, 32:268-274.

https://doi.org/10.1093/molbev/msu300

Since you used ultrafast bootstrap (UFBoot) please also cite:

Diep Thi Hoang, Olga Chernomor, Arndt von Haeseler, Bui Quang Minh,

and Le Sy Vinh (2017) UFBoot2: Improving the ultrafast bootstrap

approximation. Mol Biol Evol, in press.

https://doi.org/10.1093/molbev/msx281

SEQUENCE ALIGNMENT

------------------

Input data: 39 sequences with 56770 nucleotide sites

Number of constant sites: 32587 (= 57.4018% of all sites)

Number of invariant (constant or ambiguous constant) sites: 32587 (= 57.4018% of all sites)

Number of parsimony informative sites: 15057

Number of distinct site patterns: 5530

SUBSTITUTION PROCESS

--------------------

Model of substitution: GTR+F+R3

Rate parameter R:

A-C: 0.9838

A-G: 2.8388

A-T: 0.6359

C-G: 0.9060

C-T: 2.9739

G-T: 1.0000

State frequencies: (empirical counts from alignment)

pi(A) = 0.3019

pi(C) = 0.1923

pi(G) = 0.1998

pi(T) = 0.306

Rate matrix Q:

A -0.8521 0.1695 0.5083 0.1743

C 0.2661 -1.244 0.1622 0.8153

G 0.7679 0.1561 -1.198 0.2742

T 0.172 0.5124 0.1791 -0.8634

Model of rate heterogeneity: FreeRate with 3 categories

Site proportion and rates: (0.7711,0.3793) (0.2135,2.306) (0.01537,13.99)

Category Relative_rate Proportion

1 0.3793 0.7711

2 2.306 0.2135

3 13.99 0.01537

MAXIMUM LIKELIHOOD TREE

-----------------------

Log-likelihood of the tree: -255430.6416 (s.e. 1089.3704)

Unconstrained log-likelihood (without tree): -226122.6736

Number of free parameters (#branches + #model parameters): 87

Akaike information criterion (AIC) score: 511035.2831

Corrected Akaike information criterion (AICc) score: 511035.5532

Bayesian information criterion (BIC) score: 511813.6515

Total tree length (sum of branch lengths): 0.9803

Sum of internal branch lengths: 0.5892 (60.1053% of tree length)

NOTE: Tree is UNROOTED although outgroup taxon 'Cyperus_rotundus_NC_050170' is drawn at root

Numbers in parentheses are SH-aLRT support (%) / aBayes support / ultrafast bootstrap support (%)

+--Achnatherum_splendens_MK704435

+--| (74.6/0.865/79)

| +--Oryzopsis_asperifolia_NC_027479

+--| (100/1/100)

| +--Stipa_purpurea_NC_029390

+--| (100/1/100)

| | +--Stipa_hymenoides_NC_027464

| +--| (100/1/100)

| +--ZMC_ZMC

+--| (100/1/100)

| | +--Agrostis_gigantea_NC_037162

| | +--| (100/1/100)

| | | +--Agrostis_stolonifera_NC_008591

| | +--| (100/1/100)

| | | | +--Alopecurus_japonicus_MN422307

| | | +--| (100/1/100)

| | | | +--Cynosurus_cristatus_KY432806

| | | | +--| (83.7/1/84)

| | | | | | +--Lolium_arundinaceum_NC_011713

| | | | | +--| (100/1/100)

| | | | | +--Lolium_perenne_NC_009950

| | | +--| (100/1/100)

| | | +--Holcus_lanatus_NC_036689

| | +--| (100/1/100)

| | | +--Littledalea_alaica_NC_037519

| +--| (100/1/100)

| +--Brachypodium_distachyon_NC_011032

+--| (99.8/1/100)

| | +--Chikusichloa_aquatica_NC_027184

| | +--| (100/1/100)

| | | +--Chikusichloa_mutica_NC_041081

| | +--| (100/1/100)

| | | +--Zizania_latifolia_NC_029401

| +--| (100/1/100)

| | +--Leersia_japonica_NC_034766

| +--| (100/1/100)

| +--Leersia_perrieri_KY347906

+-----------------------------------------------------| (100/1/100)

| | +--Aeluropus_lagopoides_NC_042858

| | +--| (100/1/100)

| | | | +--Chloris_truncata_NC_032033

| | | | +--| (100/1/100)

| | | | | +--Chloris_virgata_NC_032034

| | | | +--| (91.8/0.999/93)

| | | | | +--Cynodon_dactylon_NC_034680

| | | | +--| (100/1/100)

| | | | | +--Enteropogon_ramosus_NC_042834

| | | | +--| (100/1/100)

| | | | | +--Eleusine_indica_NC_030486

| | | +--| (50.8/0.878/68)

| | | +--Dactyloctenium_radulans_NC_042838

| | +--| (98.6/1/97)

| | | | +--Arundo_donax_NC_037077

| | | | +--| (100/1/100)

| | | | | +--Arundo_plinii_NC_034652

| | | +--| (100/1/100)

| | | +--Molinia_caerulea_MF035989

| +--| (100/1/100)

| | +--Alloteropsis_angusta_NC_027951

| | +--| (100/1/100)

| | | | +--Setaria_viridis_NC_028075

| | | +--| (100/1/100)

| | | | +--Urochloa_brizantha_NC_030067

| | | +--| (100/1/100)

| | | +--Urochloa_decumbens_NC_030066

| +--| (100/1/100)

| | +--Andropogon_ascinodis_NC_040129

| | +--| (100/1/100)

| | | +--Sorghum_bicolor_NC_008602

| +--| (100/1/100)

| | +--Tripsacum_dactyloides_NC_037087

| +--| (100/1/100)

| +--Zea_nicaraguensis_KU291447

|

+----------------------Cyperus_rotundus_NC_050170

|

+----Eleocharis_dulcis_NC_047447

Tree in newick format:

(((((((Achnatherum_splendens_MK704435:0.0020528146,Oryzopsis_asperifolia_NC_027479:0.0019038864)74.6/0.865/79:0.0000469392,Stipa_purpurea_NC_029390:0.0021618326)100/1/100:0.0006264468,(Stipa_hymenoides_NC_027464:0.0016168156,ZMC_ZMC:0.0023468574)100/1/100:0.0036307682)100/1/100:0.0025861686,((((Agrostis_gigantea_NC_037162:0.0016174059,Agrostis_stolonifera_NC_008591:0.0021824083)100/1/100:0.0075589618,(Alopecurus_japonicus_MN422307:0.0068376672,((Cynosurus_cristatus_KY432806:0.0101343330,(Lolium_arundinaceum_NC_011713:0.0114112524,Lolium_perenne_NC_009950:0.0021690396)100/1/100:0.0063139546)83.7/1/84:0.0003576034,Holcus_lanatus_NC_036689:0.0059478546)100/1/100:0.0008110167)100/1/100:0.0036424493)100/1/100:0.0038513055,Littledalea_alaica_NC_037519:0.0059976767)100/1/100:0.0024102856,Brachypodium_distachyon_NC_011032:0.0184589494)100/1/100:0.0026889881)100/1/100:0.0084811560,(((Chikusichloa_aquatica_NC_027184:0.0001766673,Chikusichloa_mutica_NC_041081:0.0003078209)100/1/100:0.0027641238,Zizania_latifolia_NC_029401:0.0060763183)100/1/100:0.0017725929,(Leersia_japonica_NC_034766:0.0003958234,Leersia_perrieri_KY347906:0.0006303286)100/1/100:0.0121888569)100/1/100:0.0085957353)99.8/1/100:0.0029588614,(((Aeluropus_lagopoides_NC_042858:0.0053765196,(((((Chloris_truncata_NC_032033:0.0020353843,Chloris_virgata_NC_032034:0.0030586599)100/1/100:0.0026590082,Cynodon_dactylon_NC_034680:0.0051179555)91.8/0.999/93:0.0001173417,Enteropogon_ramosus_NC_042834:0.0026018027)100/1/100:0.0011419112,Eleusine_indica_NC_030486:0.0053016037)100/1/100:0.0018767944,Dactyloctenium_radulans_NC_042838:0.0069660548)50.8/0.878/68:0.0001341843)100/1/100:0.0092141761,((Arundo_donax_NC_037077:0.0000947096,Arundo_plinii_NC_034652:0.0003384644)100/1/100:0.0056036247,Molinia_caerulea_MF035989:0.0045529338)100/1/100:0.0009209741)98.6/1/97:0.0005277167,((Alloteropsis_angusta_NC_027951:0.0112491747,(Setaria_viridis_NC_028075:0.0038901649,(Urochloa_brizantha_NC_030067:0.0002770100,Urochloa_decumbens_NC_030066:0.0003835251)100/1/100:0.0069333704)100/1/100:0.0018377820)100/1/100:0.0020392769,((Andropogon_ascinodis_NC_040129:0.0034157975,Sorghum_bicolor_NC_008602:0.0019459587)100/1/100:0.0004367908,(Tripsacum_dactyloides_NC_037087:0.0005313810,Zea_nicaraguensis_KU291447:0.0017045935)100/1/100:0.0012776797)100/1/100:0.0062322901)100/1/100:0.0048447155)100/1/100:0.0059183966)100/1/100:0.4662166435,Cyperus_rotundus_NC_050170:0.1991248709,Eleocharis_dulcis_NC_047447:0.0506997906);

CONSENSUS TREE

--------------

Consensus tree is constructed from 5000bootstrap trees

Log-likelihood of consensus tree: -255430.641582

Robinson-Foulds distance between ML tree and consensus tree: 0

Branches with support >0.000000% are kept (extended consensus)

Branch lengths are optimized by maximum likelihood on original alignment

Numbers in parentheses are bootstrap supports (%)

+--Achnatherum_splendens_MK704435

+--| (79)

| +--Oryzopsis_asperifolia_NC_027479

+--| (100)

| +--Stipa_purpurea_NC_029390

+--| (100)

| | +--Stipa_hymenoides_NC_027464

| +--| (100)

| +--ZMC_ZMC

+--| (100)

| | +--Agrostis_gigantea_NC_037162

| | +--| (100)

| | | +--Agrostis_stolonifera_NC_008591

| | +--| (100)

| | | | +--Alopecurus_japonicus_MN422307

| | | +--| (100)

| | | | +--Cynosurus_cristatus_KY432806

| | | | +--| (84)

| | | | | | +--Lolium_arundinaceum_NC_011713

| | | | | +--| (100)

| | | | | +--Lolium_perenne_NC_009950

| | | +--| (100)

| | | +--Holcus_lanatus_NC_036689

| | +--| (100)

| | | +--Littledalea_alaica_NC_037519

| +--| (100)

| +--Brachypodium_distachyon_NC_011032

+--| (100)

| | +--Chikusichloa_aquatica_NC_027184

| | +--| (100)

| | | +--Chikusichloa_mutica_NC_041081

| | +--| (100)

| | | +--Zizania_latifolia_NC_029401

| +--| (100)

| | +--Leersia_japonica_NC_034766

| +--| (100)

| +--Leersia_perrieri_KY347906

+-----------------------------------------------------|

| | +--Aeluropus_lagopoides_NC_042858

| | +--| (100)

| | | | +--Chloris_truncata_NC_032033

| | | | +--| (100)

| | | | | +--Chloris_virgata_NC_032034

| | | | +--| (93)

| | | | | +--Cynodon_dactylon_NC_034680

| | | | +--| (100)

| | | | | +--Enteropogon_ramosus_NC_042834

| | | | +--| (100)

| | | | | +--Eleusine_indica_NC_030486

| | | +--| (68)

| | | +--Dactyloctenium_radulans_NC_042838

| | +--| (97)

| | | | +--Arundo_donax_NC_037077

| | | | +--| (100)

| | | | | +--Arundo_plinii_NC_034652

| | | +--| (100)

| | | +--Molinia_caerulea_MF035989

| +--| (100)

| | +--Alloteropsis_angusta_NC_027951

| | +--| (100)

| | | | +--Setaria_viridis_NC_028075

| | | +--| (100)

| | | | +--Urochloa_brizantha_NC_030067

| | | +--| (100)

| | | +--Urochloa_decumbens_NC_030066

| +--| (100)

| | +--Andropogon_ascinodis_NC_040129

| | +--| (100)

| | | +--Sorghum_bicolor_NC_008602

| +--| (100)

| | +--Tripsacum_dactyloides_NC_037087

| +--| (100)

| +--Zea_nicaraguensis_KU291447

| (100)

+----------------------Cyperus_rotundus_NC_050170

|

+----Eleocharis_dulcis_NC_047447

Consensus tree in newick format:

(((((((Achnatherum_splendens_MK704435:0.0020528792,Oryzopsis_asperifolia_NC_027479:0.0019039153)79:0.0000471864,Stipa_purpurea_NC_029390:0.0021618499)100:0.0006265917,(Stipa_hymenoides_NC_027464:0.0016177327,ZMC_ZMC:0.0023469220)100:0.0036309222)100:0.0025869879,((((Agrostis_gigantea_NC_037162:0.0016174685,Agrostis_stolonifera_NC_008591:0.0021824700)100:0.0075605874,(Alopecurus_japonicus_MN422307:0.0068391946,((Cynosurus_cristatus_KY432806:0.0101365862,(Lolium_arundinaceum_NC_011713:0.0114139755,Lolium_perenne_NC_009950:0.0021691473)100:0.0063153810)84:0.0003579902,Holcus_lanatus_NC_036689:0.0059491673)100:0.0008115103)100:0.0036425673)100:0.0038514627,Littledalea_alaica_NC_037519:0.0059988682)100:0.0024105322,Brachypodium_distachyon_NC_011032:0.0184625806)100:0.0026894407)100:0.0084832939,(((Chikusichloa_aquatica_NC_027184:0.0001765484,Chikusichloa_mutica_NC_041081:0.0003077830)100:0.0027641695,Zizania_latifolia_NC_029401:0.0060765431)100:0.0017738236,(Leersia_japonica_NC_034766:0.0003956300,Leersia_perrieri_KY347906:0.0006307311)100:0.0121909434)100:0.0085983758)100:0.0029440346,(((Aeluropus_lagopoides_NC_042858:0.0053765137,(((((Chloris_truncata_NC_032033:0.0020348837,Chloris_virgata_NC_032034:0.0030578967)100:0.0026583583,Cynodon_dactylon_NC_034680:0.0051179119)93:0.0001172731,Enteropogon_ramosus_NC_042834:0.0026011366)100:0.0011416224,Eleusine_indica_NC_030486:0.0053015443)100:0.0018763007,Dactyloctenium_radulans_NC_042838:0.0069660241)68:0.0001341802)100:0.0092141006,((Arundo_donax_NC_037077:0.0000948181,Arundo_plinii_NC_034652:0.0003381564)100:0.0056036591,Molinia_caerulea_MF035989:0.0045529848)100:0.0009207313)97:0.0005285725,((Alloteropsis_angusta_NC_027951:0.0112493815,(Setaria_viridis_NC_028075:0.0038902306,(Urochloa_brizantha_NC_030067:0.0002769813,Urochloa_decumbens_NC_030066:0.0003831884)100:0.0069334275)100:0.0018372222)100:0.0020388812,((Andropogon_ascinodis_NC_040129:0.0034158755,Sorghum_bicolor_NC_008602:0.0019453698)100:0.0004366294,(Tripsacum_dactyloides_NC_037087:0.0005305733,Zea_nicaraguensis_KU291447:0.0017049452)100:0.0012779724)100:0.0062323560)100:0.0048445935)100:0.0059305273):0.4662570359,Cyperus_rotundus_NC_050170:0.1991196780,Eleocharis_dulcis_NC_047447:0.0507086227)100;

TIME STAMP

----------

Date and time: Mon Nov 30 01:18:46 2020

Total CPU time used: 529.25 seconds (0h:8m:49s)

Total wall-clock time used: 319.0677365 seconds (0h:5m:19s)
