# Supplementary material for: Complete chloroplast genomes of Achnatherum inebrians and comparative analyses with related species from Poaceae
Source: FEBS Open Bio. 2021 May 10;11(6):1704–18. doi: 10.1002/2211-5463.13170 (PMC8167873; doi:10.1002/2211-5463.13170)
Supplement: Supplementary file 8 — Table S7. The morphological characteristics of Achnatherum inebrians, genus Achnatherum, genus Stipa. [file FEB4-11-1704-s003.docx]

**Table S7** The morphological characteristics of *A. inebrians，*genus *Achnatherum*，genus *Stipa* [3-4,80]

|  | *Achnatherum* | *Stipa* | *A. inebrians* |
| --- | --- | --- | --- |
| glume | most 5 veins, apex acute or acuminate, thinly obtuse rounded | 3-5 veins, apex acuminate, acute, some with filiform tail tip | 3 veins，membranous, apex acute , apical tip often fractured, slightly coarse |
| lemma | 3 veins or 5 veins, 3 veins confluence, fusiform, apical two deep lobes, back with scattered pubescent hairs, margin not overlapping | 5 veins confluent, mostly terete, mostly apically unlobed, dorsal hairs scattered or in rows, margin overlapping, tightly enclosing lemma | 3 veins, back with densely pubescent hairs, about 4 mm long |
| awn | straight, slightly curved or geniculate torsion, coarse and glabrous, without articular | geniculate torsion, all glabrous or all with pinnate hairs or only awn columns or awn needles with pinnate hairs, with articular | 10-13 mm long, geniculate torsion, micro short hairs |
| basal disc | short or long | long, sharp | obtuse, with short hairs |
| anther | with hairs on top | without hairs on top | with shorter hairs on top |
| fruit | fusiform | long cylindrical | cylindrical |
